# Evaluating Minimal Residual Disease Negativity as a Surrogate Endpoint for Treatment Efficacy in Multiple Myeloma: A Meta‐Analysis of Randomized Controlled Trials

**DOI:** 10.1002/ajh.27582

**Published:** 2025-01-09

**Authors:** Ioannis Ntanasis‐Stathopoulos, Charalampos Filippatos, Anastasios Ntanasis‐Stathopoulos, Panagiotis Malandrakis, Efstathios Kastritis, Ourania E. Tsitsilonis, Meletios A. Dimopoulos, Evangelos Terpos, Maria Gavriatopoulou

**Affiliations:** ^1^ Department of Clinical Therapeutics, School of Medicine National and Kapodistrian University of Athens Athens Greece; ^2^ Department of Biology, School of Sciences National and Kapodistrian University of Athens Athens Greece

**Keywords:** endpoint, minimal residual disease, multiple myeloma, overall survival, progression‐free survival

## Abstract

This meta‐analysis examined the association between minimal residual disease (MRD) negativity and survival outcomes in 15 304 patients with multiple myeloma (MM) enrolled in randomized controlled trials published until June 2, 2024. Overall, there was a significant, negative and strong association between MRD negativity odds ratios and survival hazard ratios (β_PFS = ‐0.20, *p* < 0.001, β_OS = ‐0.12, *p* = 0.023). These associations remained significant for newly diagnosed patients (β_PFS = ‐0.35, *p* < 0.001), and they were consistent but not significant for relapsed/refractory patients (β_PFS = ‐0.06, *p* = 0.635). Sustained MRD negativity at 1 year was strongly correlated with prolonged PFS (β_PFS = ‐0.30, *p* < 0.001). In conclusion, this comprehensive meta‐analysis supports MRD as a surrogate for survival in MM.

## 1 Background

Multiple myeloma (MM) remains an incurable plasma cell dyscrasia; however, evaluating survival endpoints in clinical trials is challenging due to improved patient outcomes from novel therapies [1]. This is particularly relevant for the assessment of overall survival (OS) and progression‐free survival (PFS) in patients with newly diagnosed MM (NDMM). The recently published phase 3 trials PERSEUS and IMROZ investigated the addition of an anti‐CD38 monoclonal antibody (daratumumab and isatuximab, respectively) to the standard backbone regimen of bortezomib, lenalidomide, and dexamethasone (VRd) in transplant‐eligible and transplant‐ineligible patients with NDMM, respectively [2, 3]. Both studies reported an estimated median PFS beyond 4 years for the patient groups receiving VRd, whereas the estimated 4‐year PFS was 84.3% for the patients who received DaraVRd in the PERSEUS trial and the estimated 5‐year PFS was 63.2% for the patients who received IsaVRd in the IMROZ trial.

Although PFS has been widely accepted as a surrogate for OS in MM and a PFS benefit is sufficient to support a regulatory approval of a new drug indication, it may take several years to achieve data maturity with the novel quadruplet standards of care. Timely approval of breakthrough treatments is important for newly diagnosed patients in the light of further improving the long‐term disease control, whereas it is crucial for patients requiring salvage therapy [4]. Therefore, identifying early surrogate endpoints predictive of long‐term efficacy is essential.

The assessment of bone marrow minimal residual disease (MRD) has been endorsed by the International Myeloma Working Group (IWMG) since 2016 and it has been incorporated in the response assessment of patients with MM in the clinical practice [5]. The aim of MRD evaluation is to examine for the presence of at least 1 myeloma cell among 100 000 (sensitivity of 10^−5^) or 1 000 000 (sensitivity of 10^−6^) enucleated cells of the bone marrow with validated techniques including next generation flow cytometry or next generation sequencing [6]. MRD negativity has shown prognostic value in MM trials and previous meta‐analyses [7, 8, 9, 10, 11, 12]. Importantly, the U.S. Food and Drug Administration (FDA) Oncologic Drugs Advisory Committee (ODAC) has recently voted in favor of using MRD negativity as an early endpoint in MM clinical trials aiming at accelerated drug approvals, based on consistent independent analyses by the collaborative research group International Independent Team for Endpoint Approval of Myeloma MRD (i2TEAMM), the EVIDENCE meta‐analysis and the FDA [12, 13].

Taking all the above into consideration, this meta‐analysis assesses the association between MRD negativity and survival outcomes in MM patients enrolled in Randomized Controlled Trials (RCTs) encompassing data from major phase 2 and phase 3 studies that were published recently and were not included in previous meta‐analyses in the field.

## 2 Materials and Methods

### 2.1 Search Strategy and Eligibility of Studies

The present meta‐analysis was performed following the Preferred Reporting Items for Systematic Reviews and Meta‐Analyses (PRISMA) guidelines [14]. The study protocol was discussed and agreed upon in advance by all authors.

A systematic search was conducted in the PubMed database from conception until June 2, 2024, using the following algorithm: (MRD OR “minimal residual disease”) AND (myeloma OR “multiple myeloma”) AND (trial OR “clinical trial”).

Eligible articles included randomized controlled trials on anti‐myeloma therapies vs. standard of care or placebo controls (no multiarm) which reported MRD negativity status and Progression‐Free Survival (PFS) between arms. These were the main outcomes for this study. Secondary outcomes included reported Overall Survival (OS), Time to Next Treatment (TTNT), PFS for MRD negative versus MRD positive patients and response status. Case–control, cohort and cross‐sectional studies, case series and case reports, reviews, in vitro and animal studies were not included in this meta‐analysis. The selection of studies was conducted initially by two co‐authors (ANS and CF) by independent work and any disagreements were resolved following consultation with a senior author (INS) and team consensus.

### 2.2 Data Abstraction and Effect Estimates

The data abstraction encompassed: general information (first author's name, publication year, PubMed, and CT database ID), study characteristics (phase, blinding, follow‐up, geographic region, control, myeloma setting, participant numbers, percentage of females, age, percentage of patients with high‐risk cytogenetics in each arm), intervention characteristics (experimental and control arm treatment, time to randomization, previous lines of anti‐myeloma therapy), and outcomes (MRD negativity between arms, MRD measurement method and threshold, complete response percentage between arms, PFS, OS, TTNT with effect estimates or fourfolds with plain data, adjustment details).

If one of the above was not found in the main article, the Supporting Information was thoroughly screened. There was no shortage of required data for the purposes of the meta‐analysis. Data were independently extracted, analyzed and recorded in separate data extraction sheets by two authors (CF and ANS). The finalized data form was reached after consultation with a senior author (INS) and team consensus.

Extracted effect estimates included odds rations and hazard ratios alongside their 95% CIs (per outcome) or any other form that could be mathematically transformed or translated to these.

In case the aforementioned information was not available, crude effect estimates and 95% CIs were calculated by means of fourfolds from plain data extracted from the articles.

It was decided to not reconstruct individual patient data via published Kaplan–Meier curves or other plots in order to avoid approximation errors and thus proceed with a treatment arm vs. reference arm conclusive comparison.

### 2.3 Statistical Analyses

Statistical analyses included pooling of studies as well as post hoc univariate regressions and meta‐regressions. Common (Mantel–Haenszel with continuity correction 0.5 for zero cell frequencies) and Random‐effects (Inverse variance) models were appropriately used to calculate the pooled effect estimates (risk differences, odds ratios, hazard rations). Between‐study heterogeneity was assessed by Q‐test and I^2^ estimations. When heterogeneity was not low (I^2^ > 40%), random‐effect models results were deemed appropriate. Subgroup analyses were performed based on adjustment, myeloma setting, anti‐myeloma therapy type, follow‐up period, and geographic region.

Post hoc univariate regressions in a meta‐analytic framework and multivariate meta‐regression analyses were performed in order to assess whether MRD negativity ratio and MRD positivity risk between arms was significantly associated with lower PFS, OS, and TTNT rates and whether moderators within the study sample modified this relationship. Variables included were key study aspects that introduce heterogeneity and had 10 or more entries.

Specifically, weighted univariate regressions of outcome effect estimates on MRD negativity ratios in a meta‐analytic framework allow to test whether the effect of MRD negativity on patient outcomes varies across studies based on the MRD negativity OR itself. This approach provides insights into potential interactions or differences in the MRD‐PFS relationship across different contexts or study characteristics, enhancing the depth of analysis compared to standard univariate weighted regression.

All statistical analyses were performed using R/R‐Studio version 2024.04.2 + 764 (Posit Software, PBC).

### 2.4 Assessment of Study Quality and Risk of Bias

All records included randomized clinical trials, either blinded or open label. Risk was assessed with the implementation of the RoB:2 algorithm by Cochrane to our analysis tools [14]. Specifically, two authors carried out the assessment procedure independently (ANS and CF) and, thereafter, they reached consensus with the help of a third author (INS) in cases of disagreement.

Publication bias was evaluated in the analyses that included 10 or more study arms [15]. For this purpose, Egger's statistical test (statistical significance *p* < 0.1) [16] was implemented as well as the funnel plot inspection [17]. The evaluation of publication bias was performed using R/R‐Studio version 2024.04.2 + 764 (Posit Software, PBC).

## 3 Results

**FIGURE 1:**
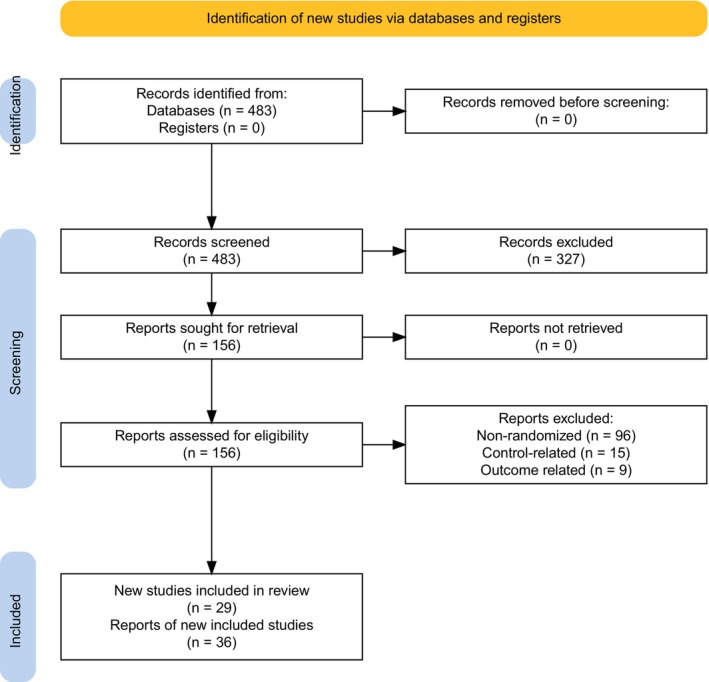
Study selection flowchart. [Color figure can be viewed at wileyonlinelibrary.com]

A total of 483 records were identified from PubMed using the search algorithm (*Search strategy and eligibility of studies*) and were assessed for eligibility. The following flowchart, created with the PRISMA 2020 Flow Diagram tool [18] portrays the successive steps in the selection of eligible studies (Figure 1).

In total, 36 articles with results from 29 randomized controlled trials were included [2, 3, 19, 20, 21, 22, 23, 24, 25, 26, 27, 28, 29, 30, 31, 32, 33, 34, 35, 36, 37, 38, 39, 40, 41, 42, 43, 44, 45, 46, 47, 48, 49, 50, 51, 52]. All articles reported crude MRD negativity numbers and PFS hazard ratios whereas secondary outcomes were reported in a subset of them. Subgroup analyses were conducted to adjust for heterogeneity in the dataset and to adjust for effect modifiers that were selected based on available qualitative evidence from the extracted records. Table 1 presents the characteristics of the included studies regarding region, patient, and disease characteristics and intervention.

**TABLE 1:** Study, patient, and intervention characteristics of selected records.

Trial and author (year)	Region	Phase	MM setting	Therapy (treatment arm)	Therapy (reference arm)	*n* (total patients)	Follow‐up (months, median)
GRIFFIN							
Voorhees et al. (2020)	USA	2	NDMM TE	D‐VRd	VRd	207	22.1
Voorhees et al. (2023)	“	“	“	“	“	“	49.6
PERSEUS							
Sonneveld et al. (2024)	Global	3	NDMM TE	D‐VRd	VRd	709	47.5
ATLAS							
Dytfeld et al. (2023)	Global	3	NDMM TE	K‐Rd	Rd	180	33.8
CASSIOPEIA							18.8
Moreau et al. (2019)	Europe	3	NDMM TE	D‐VTd	VTd	1085	35.4
Moreau et al. (2021)	“	“	“	D	OBS	886	80.1 (1st randomization)
Moreau et al. (2024)	“	“	“	“	“	“	70.6 (2nd randomization)
IFM 2009							
Attal et al. (2017)	Europe	3	NDMM TE	VRd + ASCT	VRd	700	44.0 (RVD‐alone) 43.0 (transplantation)
TOURMALINE‐MM3							
Dimopoulos et al. (2019)	Global	3	NDMM TE	Ixazomib	Placebo	656	31.0
PETHEMA/GEM2014MAIN							
Rosinol et al. (2023)	Europe	3	NDMM TE	VRd	Rd	332	69.0
VCAT							
Horvath et al. (2019)	Global	3	NDMM TE	VTP	TP	203	22.3 (VTP arm) 23.2 (TP arm)
CARDAMON							
Yong et al. (2023)	UK	3	NDMM TE	HSCT	KCd	218	40.2
DETERMINATION							
Richardson et al. (2022)	USA	3	NDMM TE	ASCT + VRd	VRd	722	76.0
Combined TOURMALINE							
Paiva et al. (2023)	Global	3	NDMM TE & TIE	Ixazomib	Placebo	1362	14.0
	“	“	“	“	“	“	28.0
OCTANS							
Fu et al. (2023)	Asia	3	NDMM TIE	D‐VMP	VMP	220	12.3
	“	“	“	“	“	“	41.2
ENDURANCE							
Kumar et al. (2020)	USA	3	NDMM TIE	KRd	Rd	1087	9.0
MAIA							
Facon et al. (2021)	Global	3	NDMM TIE	D‐Rd	Rd	737	28.0
ALCYONE							
Mateos et al. (2018)	Global	3	NDMM TIE	D‐VMP	VMP	706	16.5
Mateos et al. (2020)	“	“	“	“	“	“	40.1
CLARION							
Facon et al. (2019)	Global	3	NDMM TIE	KMP	VMP	955	22.0
EMN20							
Bringhen et al. (2023)	Global	3	NDMM TIE	K‐Rd	Rd	82	24.9
IMROZ							
Facon et al. (2024)	Global	3	NDMM TIE	Isa‐VRd	VRd	446	59.7
APOLLO							
Dimopoulos et al. (2021)	Europe	3	RRMM	D‐Pd	Pd	304	16.9
BOSTON							
Grosicki et al. (2020)	Global	2	RRMM	SVd	Vd	402	13.2 (SelVd) 16.5 (Vd)
CANDOR							
Usmani et al. (2022)	Global	3	RRMM	KdD	Kd	466	50.4
CASTOR							
Spencer et al. (2018)	Global	3	RRMM	D‐Vd	Vd	498	19.4
Mateos et al. (2020)	“	“	“	“	“	“	40.0
POLLUX							
Dimopoulos et al. (2016)	Global	3	RRMM	D‐Rd	Rd	569	13.5
Dimopoulos et al. (2023)a	“	“	“	“	“	“	79.7
IKEMA							
Moreau et al. (2021)	Global	3	RRMM	Isa‐Kd	Kd	302	20.7
Martin et al. (2023)	“	“	“	“	“	“	44.0
DREAMM‐7							
Hungria et al. (2024)	Global	3	RRMM	BVd	DVd	494	28.2
DREAMM‐8							
Dimopoulos et al. (2024)	Global	3	RRMM	BPd	PVd	302	21.8
KarMMa‐3							
Rodriguez‐Otero et al. (2023)	Global	3	RRMM	Ide‐cel	SoC	386	18.6
CARTITUDE‐4							
San‐Miguel et al. (2023)	Global	3	RRMM	Cilta‐cel	SoC	419	15.9
DREAMM‐3							
Dimopoulos et al. (2023b)	Global	3	RRMM	B	Pd	325	11.5 (B) 10.8 (Pd)

*Note*: Multiple entries referring to the same clinical trial provide varying outcomes for different timepoints.

Abbreviations: ASCT, autologous stem cell transplantation; B, belantamab mafodotin; D, daratumumab; d, dexamethasone; Isa, isatuximab; K, carfilzomib; M, melphalan; NDMM TE, newly diagnosed multiple myeloma transplant eligible; NDMM TIE, newly diagnosed multiple myeloma transplant ineligible; P, pomalidomide; R, lenalidomide; RRMM, relapsed/refractory multiple myeloma; S, selinexor; T, thalidomide; V, bortezomib.

### 3.1 Meta‐Analysis

#### 3.1.1 Final Follow‐Up (Base Case) Analysis

**FIGURE 2:**
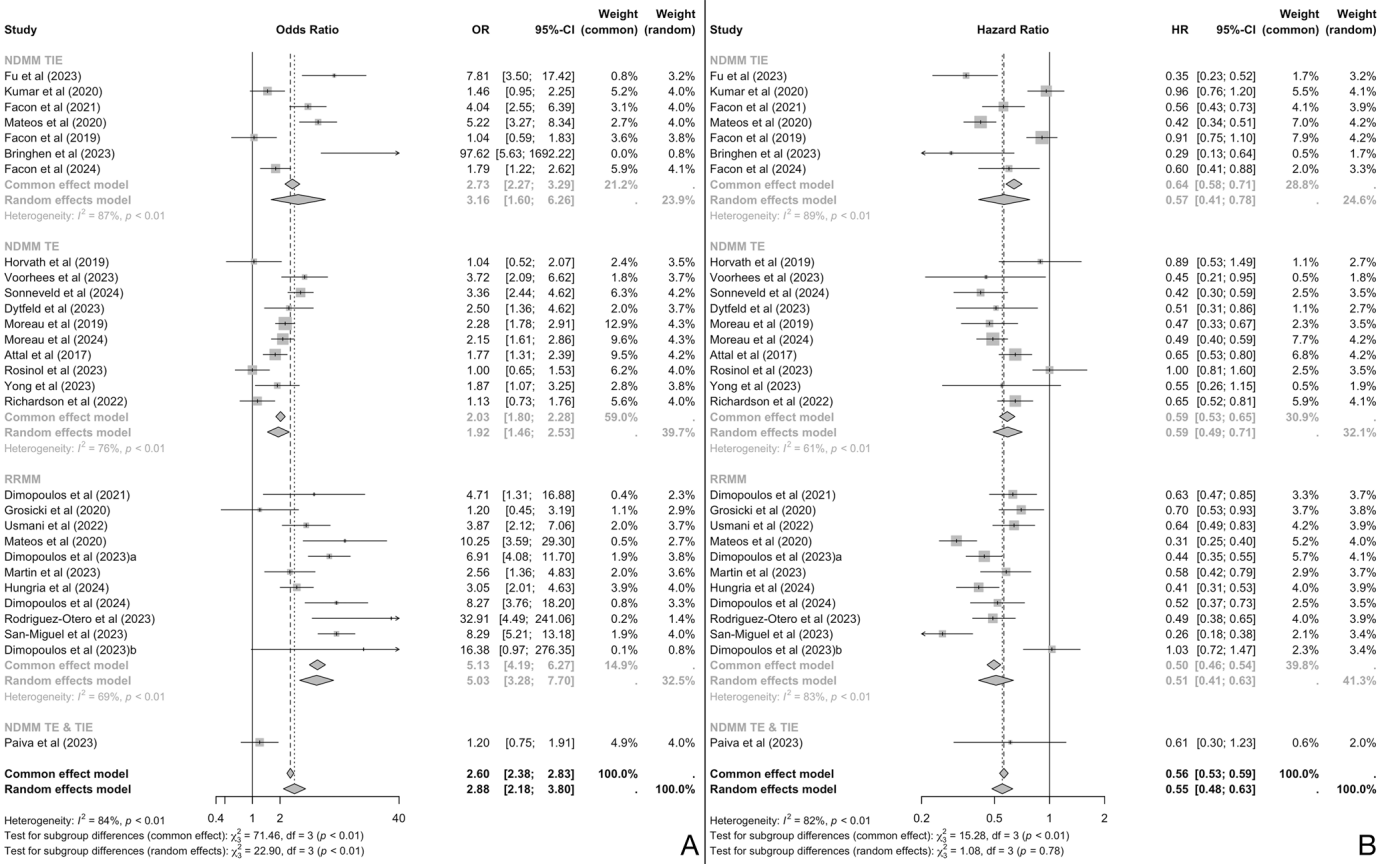
MRD negativity (OR) (A) and PFS (HR) (B) pooled‐estimates for the whole study population (by MM setting). 95%‐CI, 95% confidence interval; HR, hazard ratio; MM, multiple myeloma; MRD, minimal residual disease; NDMM TE, newly diagnosed multiple myeloma transplant eligible; NDMM TIE, newly diagnosed multiple myeloma transplant ineligible; OR, odds ratio; PFS, progression free survival; RRMM, relapsed/refractory multiple myeloma.

Out of the 36 included records, 7 were interim analyses and were thus excluded for the base case, final (and longest) follow‐up meta‐analysis. In the 29 studies included, patients in treatment arms exhibited statistically significant higher odds of MRD negativity when compared to these of reference arms [OR 2.88, 95% CI (2.18, 3.80)] (Figure 2A). This significant and favorable pooled‐estimate of MRD negativity OR comes in line with statistically significant favorable PFS [HR 0.55, 95% CI (0.48, 0.63)] for the treatment arms (Figure 2B). Moreover, 16 study records reported OS outcomes (HRs) and were subjected to meta‐analysis, generating a significant and favorable pooled‐estimate [HR 0.82, 95% CI (0.72, 0.90)] for the treatment arms (Figure S8).

**FIGURE 3:**
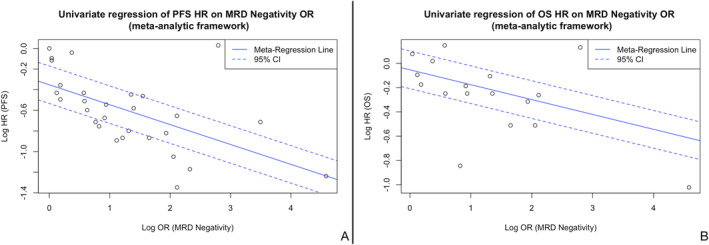
Univariate regression in a meta‐analytic framework for the association of MRD. Negativity (OR) on PFS (HR) (A) and MRD negativity (OR) on OS (HR) (B). 95% CI, 95% confidence interval; HR, hazard ratio; MRD, minimal residual disease; OR, odds ratio; PFS, progression free survival. Regression equation (2A): Log(PFS_HR) = −0.35 − 0.20 × Log(MRDneg_OR), *p* < 0.001, Regression equation (2B): Log(OS_HR) = −0.12*log(MRDneg_OR), *p* = 0.023. [Color figure can be viewed at wileyonlinelibrary.com]

Subsequent univariate regressions (meta‐analytic framework) and meta‐regression analyses revealed a strong statistically significant and negative association between MRD negativity odds ratios and survival outcomes hazard rations (Figure 2). For PFS, the slope of the univariate regression was β = −0.20 (*p* < 0.001) with R^2^ = 0.33 and for OS β = −0.12 (*p* = 0.023) with R^2^ = 0.15 (Figure 3). The statistically significant coefficients and the proportion of variance explained by R^2^ for the PFS‐MRD and OS‐MRD indicate that PFS and OS HRs associate with MRD negativity ORs. These associations for MRD negativity ORs and PFS HRs were further validated for NDMM patients, where there was a strong association (β = −0.35, *p* < 0.001, R^2^ = 0.79), while for RRMM patients the results were consistent but not significant (β = −0.06, R^2^ = 0.0, *p* = 0.635).

Post hoc multivariate meta‐regression analysis on the fit of PFS HRs and MRD negativity ORs accounted for a part of the heterogeneity between studies (I^2^ = 73%) while also underlining a considerable association (coeff. = −0.16, *p* = 0.017, R^2^ = 0.37). MM disease setting, MRD assessment technology and MRD sensitivity threshold were not proven to modify the relationship between PFS HRs and MRD negativity ORs (Table S1). Post hoc multivariate meta‐regression analysis on the fit of OS HRs and MRD negativity ORs accounted for heterogeneity between studies (I^2^ = 0%), but only MRD assessment technology was proven to modify the relationship between the two effect outcomes (Table S2).

Additionally, higher risk of being MRD positive in‐between treatment and reference arms [RD = −0.17, 95% CI (−0.21, −0.12)] (Figure S13) was associated with smaller differences in PFS and OS HRs (slope of univariate regressions β = 2.38 (*p* < 0.001) with R^2^ = 0.73 for PFS and β = 1.52 (*p* < 0.001) with R^2^ = 1 for OS) (Figures S17 and S18), respectively.

Subsequent subgroup analyses by treatment type [anti‐BCMA (CAR T‐cells, belantamab mafodotin), anti‐CD38 or other] revealed higher MRD negativity odds for patients on anti‐BCMA [OR = 7.13, 95% CI (3.54, 14.36)], followed by anti‐CD38 [OR = 3.61, 95% CI (2.75, 4.74)] and other treatments [OR = 1.40, 95% CI (1.16, 1.70)] (Figure S3). These observations were comparable to those of PFS HRs, as patients on anti‐BCMA had the most favorable outcomes compared to those of reference arms [HR = 0.49, 95% CI: (0.32, 0.75)], followed by anti‐CD38 [HR = 0.48, 95% CI (0.42, 0.54)] and other treatments [HR = 0.74, 95% CI: (0.63, 0.86)] (Figure S6). The trends for OS were similar (Figure S11).

#### 3.1.2 1‐Year Follow‐Up Meta‐Analysis

A total of 11 records reported results at a 1‐year follow‐up period. In this subgroup, patients of the treatment arm exhibited statistically significant increased odds for MRD negative status [OR = 2.93, 95% CI (1.84, 4.65)] paired with statistically significant reduced hazard rates for disease progression or death (PFS) [HR = 0.57, 95% CI (0.44, 0.73)] when compared to the reference arm (Figures S24 and S27). Only five records reported OS outcome HRs and the random‐effects model pooled‐effect estimate was not statistically significant [HR = 0.82, 95% CI (0.61, 1.10)] (Figure S30).

Continued exploration for the relationship between MRD status and survival outcomes utilized subsequent univariate regressions in a meta‐analytic manner. For PFS, the slope of the univariate weighted regression was −0.14 (*p* = 0.378, R^2^ = 0.0). As R^2^ hints, heterogeneity within the associations is considerable and that leads to a non‐statistically but still consistent negative association between PFS HRs and MRD negativity ORs (Figure S34).

Post hoc multivariate meta‐regression analysis on the fit of PFS HRs and MRD negativity ORs based on MM disease setting, MRD assessment technology and MRD sensitivity threshold did not account for heterogeneity between studies (I^2^ = 78%) and none of these factors were proven to significantly modify the relationship between the assessed outcomes (Table S3).

#### 3.1.3 2‐Year Follow‐Up Meta‐Analysis

A total of 12 records reported results at 2‐year follow‐up periods. In this subgroup, patients of the treatment arms exhibited statistically significant increased odds for MRD negative status [OR = 2.98, 95% CI (1.73, 5.14)] paired with statistically significant reduced hazard rates for disease progression or death (PFS) [HR = 0.55, 95% CI (0.43, 0.70)] (Figures S38 and S41). Overall, survival HRs were only reported in 3 records and taking into account the high heterogeneity of the data set, no meta‐analysis was conducted.

Subsequent univariate regressions in a meta‐analytic manner revealed a strong statistically significant and negative association between PFS HRs and MRD negativity ORs with a slope of −0.21 (*p* < 0.001) with R^2^ = 0.62. The high proportion of variance explained by R^2^ for PFS‐MRD underlines the strong association (Figure S45).

Post hoc multivariate meta‐regression analysis on the fit of PFS HRs and MRD negativity ORs based on MM disease setting, MRD assessment technology and MRD sensitivity threshold did not account for heterogeneity between studies (I^2^ = 55%) and none of these factors were proven to significantly modify the relationship between the assessed outcomes (Table S4).

#### 3.1.4 3‐Year Follow‐Up Meta‐Analysis

Five records provided MRD status data and survival outcomes for 3‐year follow‐up periods and were subjected to meta‐analysis. Results were statistically significant, as patients in treatment arms exhibited increased odds for MRD negative status [OR = 2.94, 95% CI (1.55, 5.59)] and lower PFS hazard rates [HR = 0.48, 95% CI (0.36, 0.64)] (Figures S48 and S51). In these records, overall survival outcomes were either premature or scarce and thus are not included in this sub‐meta‐analysis.

#### 3.1.5 4‐Year Follow‐Up Meta‐Analysis

Seven records provided outcomes between arms for 4‐year follow‐up periods. Patients of the treatment arms had statistically significant higher odds of being MRD negative when compared to those of the reference arms [OR = 3.64, 95% CI (2.47, 5.37)] while also having statistically significant favorable PFS [HR = 0.51, 95% CI (0.42, 0.61)] and OS [HR = 0.80, 95% CI (0.68, 0.92)] (Figures S57 and S60).

#### 3.1.6 Sustained MRD Negativity for 12 Months

Ten records reported outcomes for 12‐month sustained MRD negativity in terms of pure numbers between arms. As such, a meta‐analysis was conducted in order to test the association between 12‐month sustained MRD negativity within arms (OR) and PFS (HR).

In this subgroup, patients in the treatment arms had as 2.68 times the odds to sustain MRD negative status for 12 months [OR = 2.68, 95% CI (1.67, 4.31)] while also exhibiting lower hazard rates for disease progression or death [PFS HR = 0.52, 95% CI (0.43, 0.63)] (Figures S71 and S74). Data for OS were either scarce or premature and thus an analysis was not possible.

Further exploration via meta‐analytic oriented regression revealed a strong statistically significant and negative association. For PFS, the slope of the univariate regression was −0.30 (*p* < 0.001) with R^2^ = 0.91. The high proportion of variance explained by R^2^ for PFS‐MRD indicates that PFS HRs associate strongly with MRD negativity ORs (Figure S78).

Post hoc multivariate meta‐regression analysis on the fit of PFS HRs and MRD negativity ORs based on MM disease setting, MRD assessment technology and MRD sensitivity threshold showed no significant interactions and accounted for a large percentage heterogeneity between studies (I^2^ = 30%) (Table S5).

#### 3.1.7 TTNT vs. MRD Negativity Analysis

Six records from the total sample reported TTNT outcomes in terms of HRs between arms. Patients in the treatment arms exhibited a pooled 59% lower hazard rate for needing a next treatment regimen at any given time of the follow‐up period [HR = 0.41, 95% CI (0.31, 0.54)] when compared to those of the reference arms while also having increased odds of MRD negative status [OR = 3.73, 95% CI (1.90, 7.32)] (Figures S81 and S84).

#### 3.1.8 PFS in MRD Negative vs. MRD Positive Patients

Only 5 out of the 35 records reported PFS outcomes for MRD negative vs. MRD positive patients. These were results from five separate trials and were meta‐analyzed in order to produce a pooled‐effect survival outcome. Results of random‐effects analysis hinted at a 20% lower hazard rate for disease progression or death for MRD negative patients when compared to MRD positive patients [HR = 0.80, 95% CI (0.62, 1.03)] (Figure S89).

Additional subgroup analyses by region, follow‐up period, treatment type and adjustment were conducted in order to adjust for heterogeneity and funnel plots were constructed to assess for publication bias, as appropriate (Supporting Information Figures). Overall, the risk of bias of the included studies was assessed as low (Table S6), in terms of the randomization process, deviations from intended interventions, missing outcome data, outcome measurements, and selective reporting of the results.

## 4 Discussion

The use of surrogate endpoints in myeloma clinical trials is essential for the efficient and successful advancement of novel treatment. MM usually requires prolonged investigations to evaluate significant clinical outcomes such as OS. Nevertheless, the gradual course of the illness and extended survival may result in delays in accessing potentially life‐saving treatments while awaiting OS statistics to mature. Surrogate endpoints, such as PFS and MRD, provide early signs of therapy success and potentially forecast long‐term results without necessitating prolonged follow‐up [53, 54]. The use of these surrogate indicators enables clinicians to evaluate and select effective treatments more rapidly, therefore expediting regulatory approval procedures and enhancing patient access to innovative medications. Nonetheless, properly evaluating surrogate endpoints is crucial to guarantee they accurately represent genuine therapeutic benefits, hence fostering confidence among regulatory agencies, healthcare professionals, and patients [54].

Our updated meta‐analysis adds to the body of evidence supporting MRD as a surrogate endpoint for PFS and OS in MM, and it aligns with previous meta‐analyses in this field indicating strong associations between MRD negativity and PFS/OS both at trial‐ and individual patient‐level [7, 8, 9, 10, 11, 12, 13]. Furthermore, it also encompasses data from recently published studies on quadruplet regimens in the upfront treatment, as well as anti‐BCMA CAR T‐cells and conjugated monoclonal antibodies in the relapsed/refractory setting. All the meta‐analyses in the field have included studies that vary in design, treatment strategies, prior lines of treatment, methods, sensitivity threshold, and schedule of MRD assessments. Actually, this heterogeneity is representative of a wide spectrum of clinical scenarios and constitutes a strength of the observed association between MRD negativity and survival outcomes.

MRD is a reliable and biological plausible predictor of survival outcomes. Although the achievement of a complete response (CR) has been associated with improved survival outcomes, there were cases with early relapses even after attaining a CR [55, 56, 57]. The introduction of MRD evaluation in the clinical practice enabled a better prognostic discrimination of patients based on more sensitive techniques [58]. A pooled analysis of data from 609 NDMM patients enrolled in the GEM2000, GEM2005MENOS65, and GEM2010MAS65 clinical trials showed that MRD negative status exceeded the prognostic significance of CR attainment for PFS and OS, irrespective of treatment modality or risk category. Indeed, patients with MRD positive CR status had similar survival outcomes with patients achieving near CR or PR after frontline treatment. Therefore, the survival advantage of CR response category is primarily driven by MRD negative cases [59].

Based on the current evidence, it seems that an MRD sensitivity threshold of 10^−5^ is sufficient for separating distinct prognostic groups of patients. Although next‐generation sequencing methods may deepen the limit of clonal plasma cell detection, both our and previously published meta‐analyses showed no differences between 10^−5^ and 10^−6^ sensitivity thresholds. The next‐generation flow cytometry enables the detection of aberrant plasma cells at the level of 2 × 10^−6^, whereas it is reliable, easily accessible and widely available [60].

The evidence supporting MRD as a surrogate endpoint for PFS and OS is consistent across treatment modalities. Our subgroup analyses showed that the observed associations are retained among studies including regimens with anti‐CD38 monoclonal antibodies, as well as among those including anti‐BCMA treatments. Importantly, accumulating data support the value of MRD negativity as a predictor of PFS in patients receiving novel immunotherapies including CAR T‐cells and bispecific antibodies [60, 61, 62].

One of the main limitations of our meta‐analysis pertains to the limited availability of raw data in the individual studies regarding the outcomes of patients in each treatment arm according to MRD status. For this reason, we followed an indirect approach calculating MRD negativity ORs between treatment arms and linking them to the corresponding PFS and OS HRs. Until the end of search date, there were no published phase 2 or 3 RCTs comparing any bispecific antibody with standard of care. Therefore, the exact role of MRD with these treatments remains to be clarified. Moreover, although we showed significant results in the NDMM setting, there was a lack of statistical significance in the RRMM setting in the subgroup analysis. This may be due to the heterogeneity in treatment modalities and population characteristics including prior lines of treatment and variable refractoriness to drug classes. Furthermore, serological assessment of MRD by evaluating circulating tumor cells or by means of mass spectrometry are promising approaches that may be able to substitute the need for bone marrow aspiration [63, 64, 65, 66, 67]; however, these should be prospectively evaluated in clinical studies.

Following the approval as an early endpoint from regulatory agencies, the integration of MRD as a primary endpoint in MM clinical trials is anticipated to have a significant impact on the clinical practice, as well. Apart from evaluating treatment efficacy and comparing different treatment regimens, MRD‐based treatment decisions may lead to therapy intensification or de‐escalation, introducing early rescue interventions, adapting maintenance treatment duration, and intensity. Several ongoing clinical trials and prospective studies are evaluating MRD‐driven therapeutic decisions by de‐intensifying or interrupting treatment in patients with sustained MRD negativity and re‐intensifying or re‐introducing treatment upon MRD resurgence [2, 68, 69, 70, 71].

In conclusion, this comprehensive meta‐analysis of RCTs enrolling 15 304 MM patients demonstrates that patient groups which exhibited higher odds of MRD negativity also had favorable survival outcomes in terms of both PFS and OS compared to others. This significant and strong association underscores MRD negativity as a potent surrogate endpoint for treatment efficacy in MM. These findings were consistent across all analyzed time points, reinforcing the robustness of MRD negative status as an indicator of long‐term treatment benefits.

## Ethics Statement

The authors have nothing to report.

## Conflicts of Interest

The authors declare no conflicts of interest.

## Supporting information


Data S1.

## Data Availability

The data that supports the findings of this study are available in the Supporting Information of this article.
