# Supplementary material for: Evaluating Minimal Residual Disease Negativity as a Surrogate Endpoint for Treatment Efficacy in Multiple Myeloma: A Meta‐Analysis of Randomized Controlled Trials
Source: Am J Hematol. 2025 Jan 9;100(3):427–38. doi: 10.1002/ajh.27582 (PMC11803549; doi:10.1002/ajh.27582)
Supplement: Supplementary file 1 — Data S1. [file AJH-100-427-s001.docx]

*Final follow-up (base case) analysis*

**Figure S1.** MRD negativity (OR) pooled-estimate for the base-case analysis (by region)


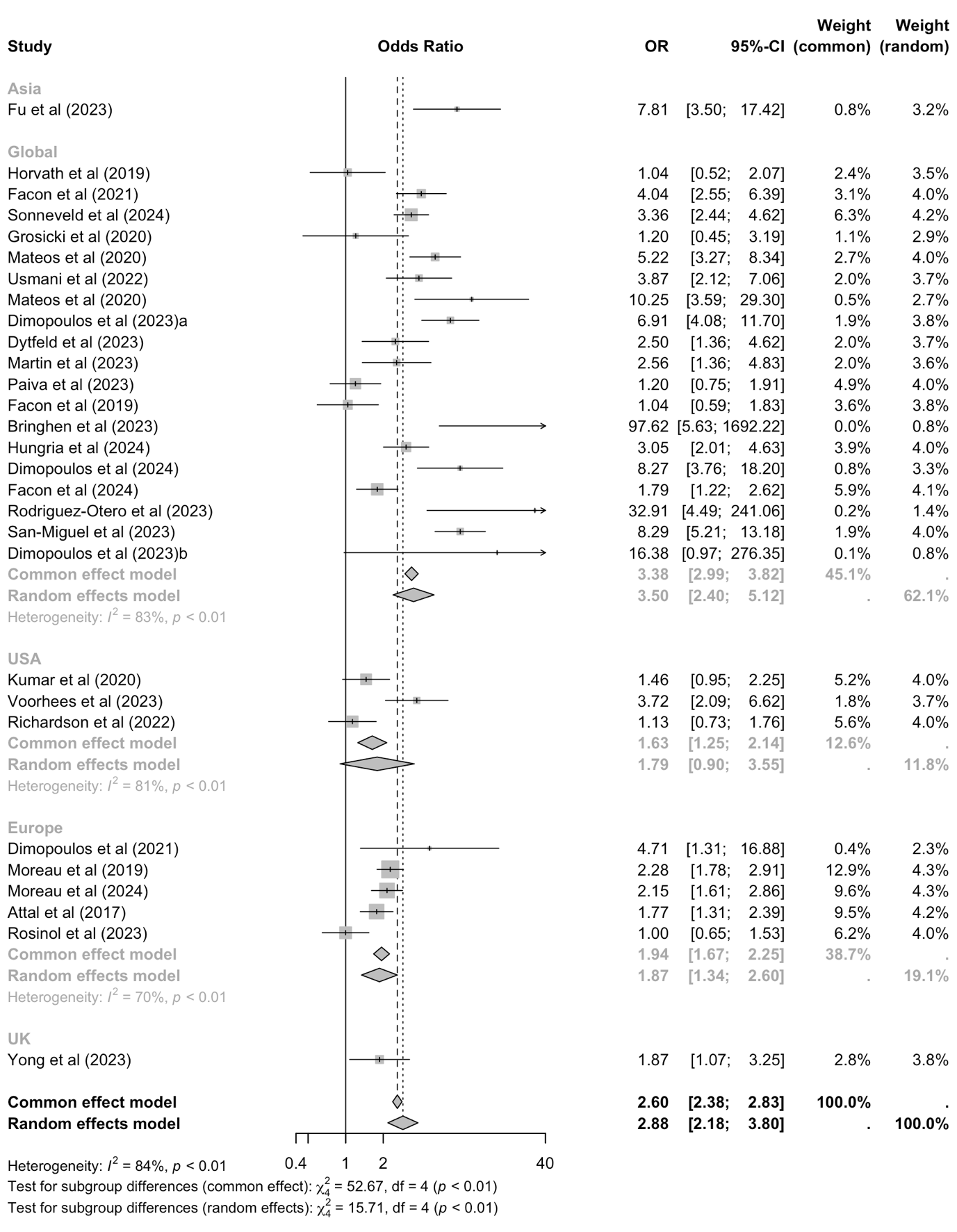


**Figure S2.** MRD negativity (OR) pooled-estimate for the base-case analysis (by follow-up)


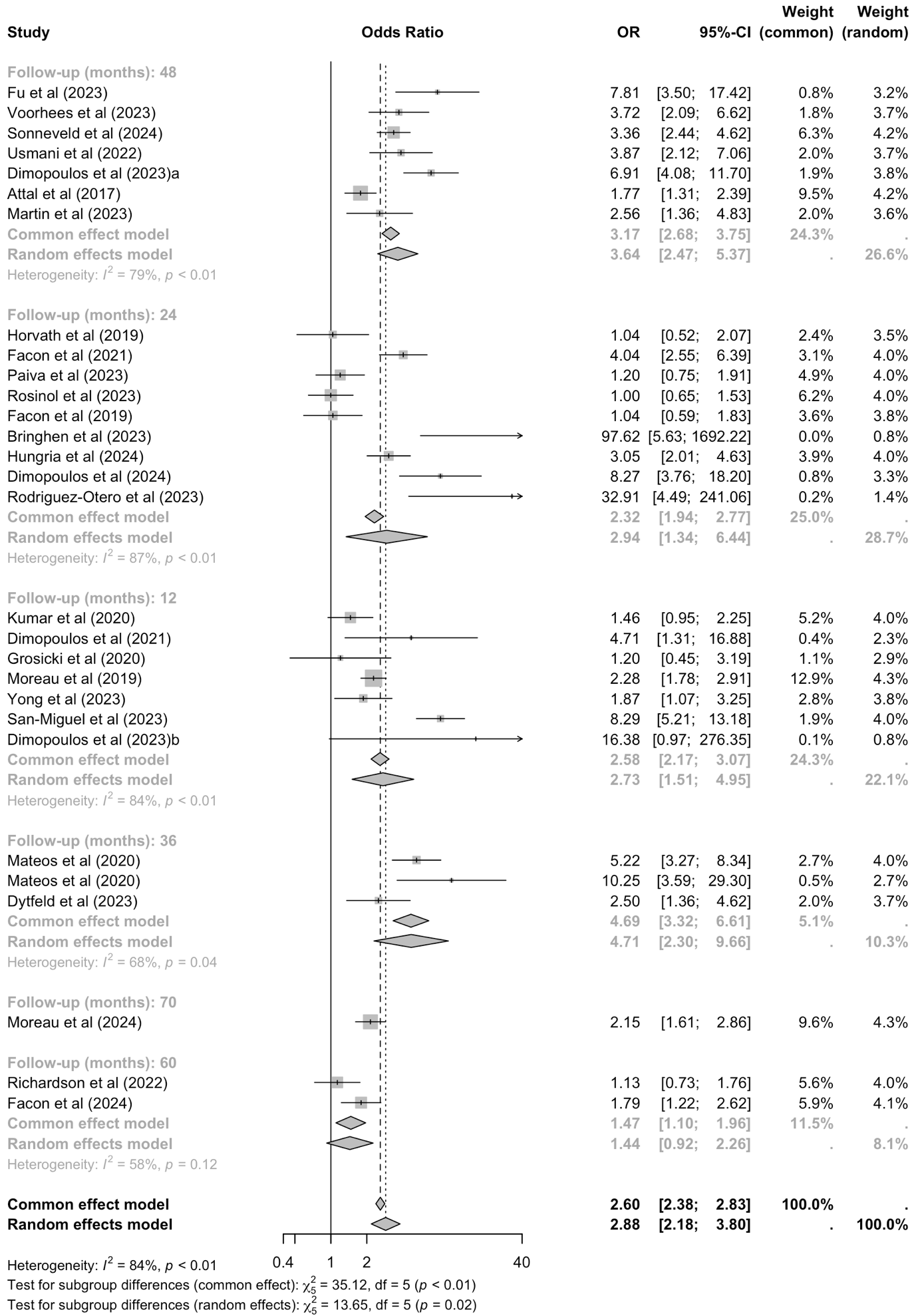


**Figure S3.** MRD negativity (OR) pooled-estimate for the base-case analysis (by treatment)


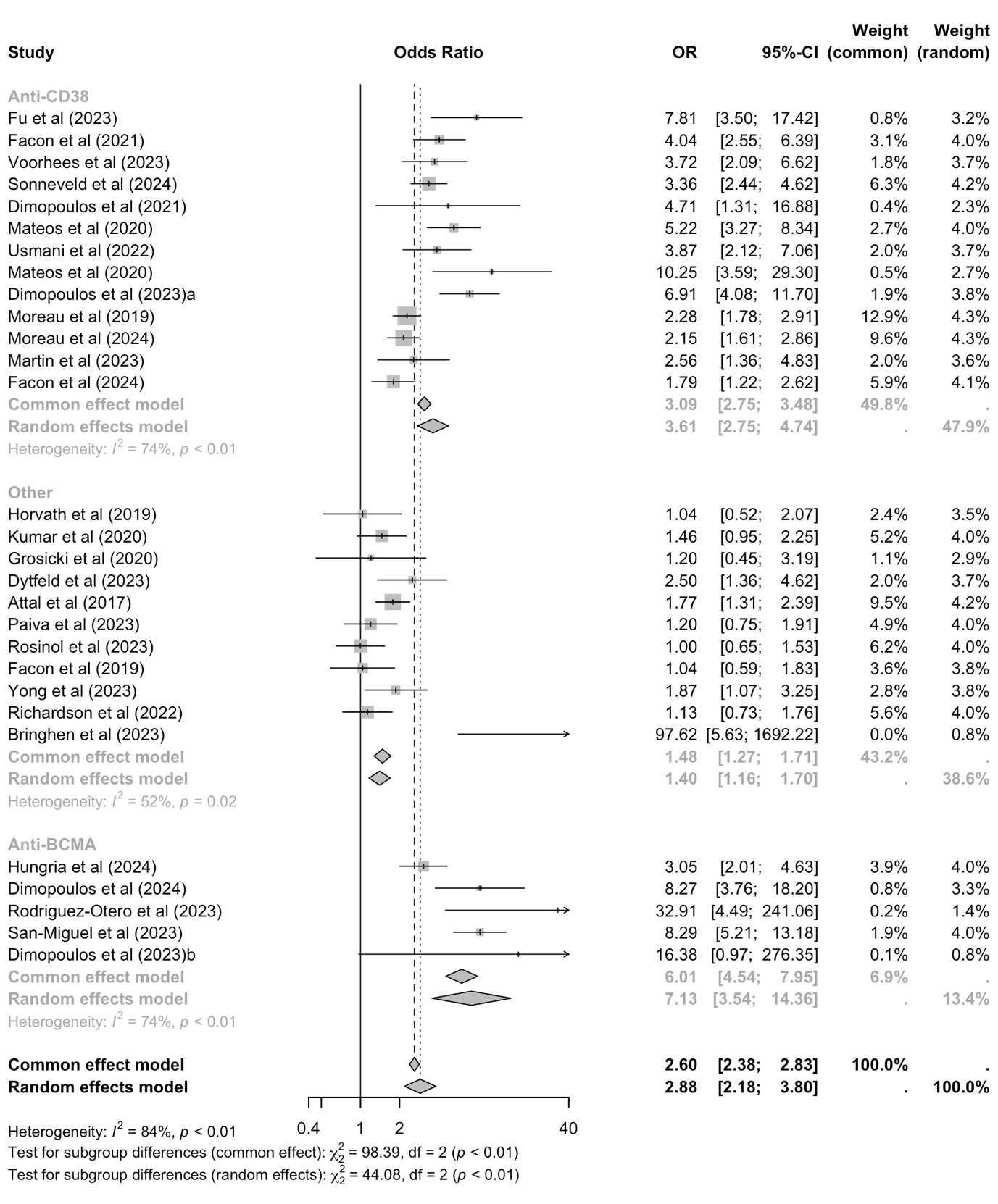


**Figure S4.** PFS (HR) pooled-estimate for the base-case analysis (by region)


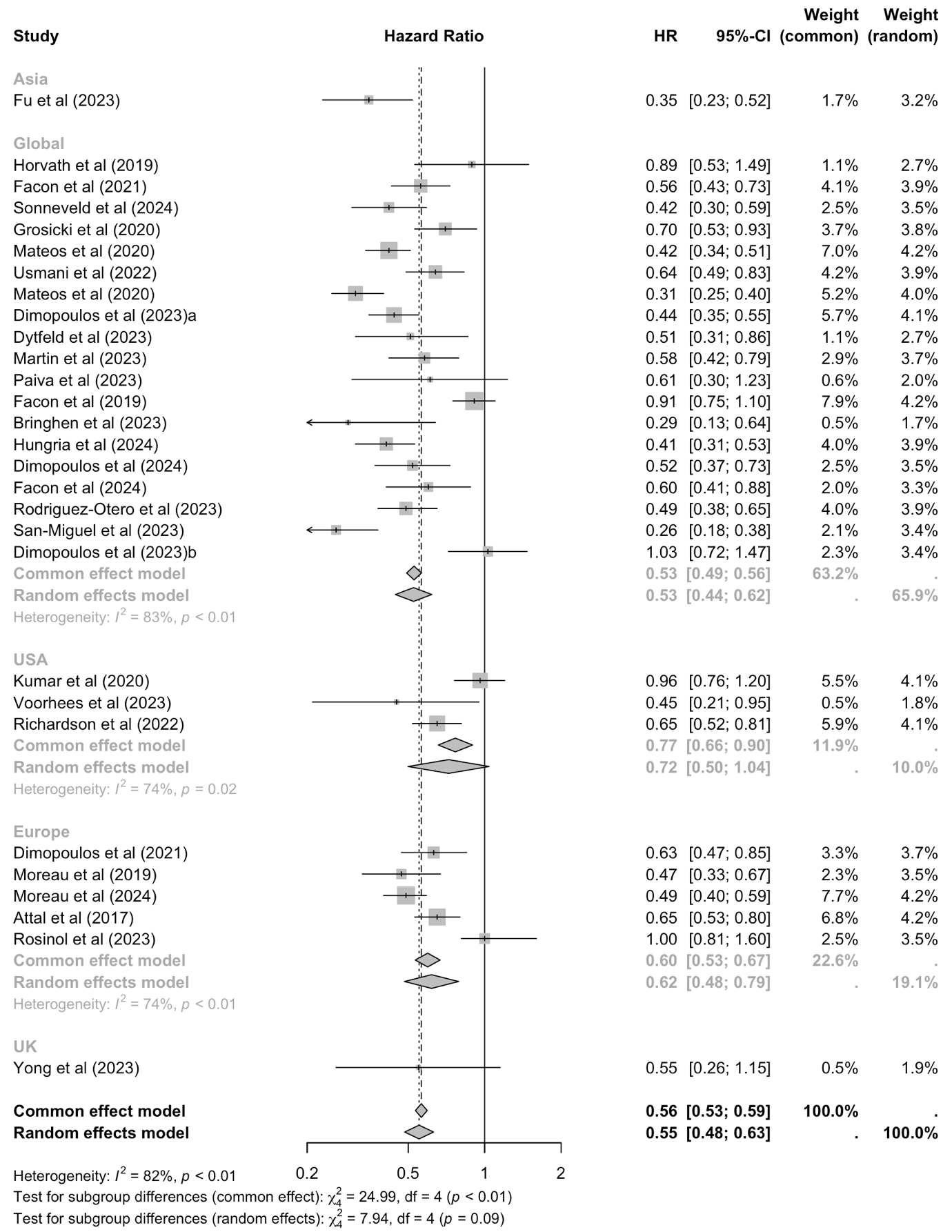


**Figure S5.** PFS (HR) pooled-estimate for the base-case analysis (by follow-up)


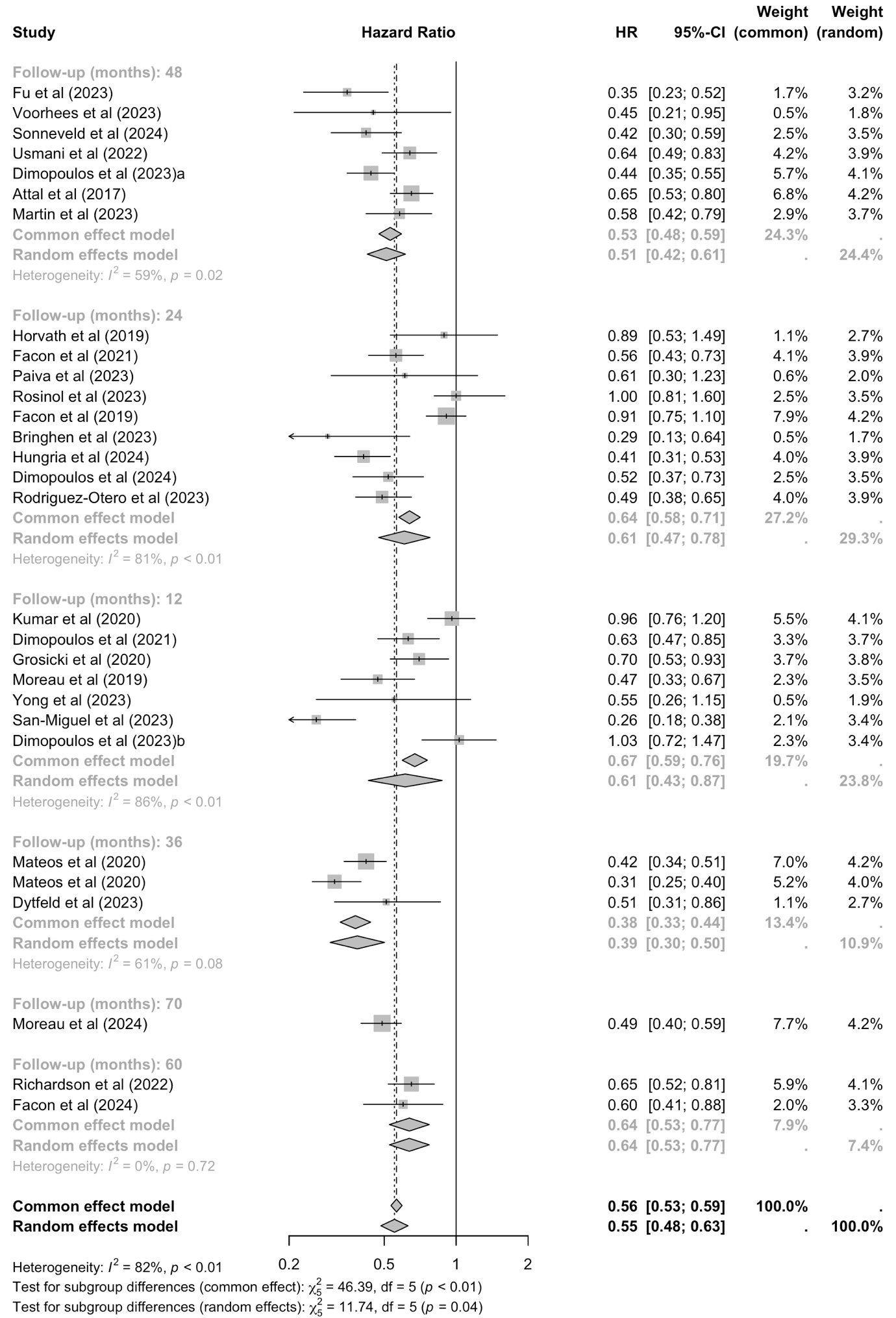


**Figure S6.** PFS (HR) pooled-estimate for the base-case analysis (by treatment)


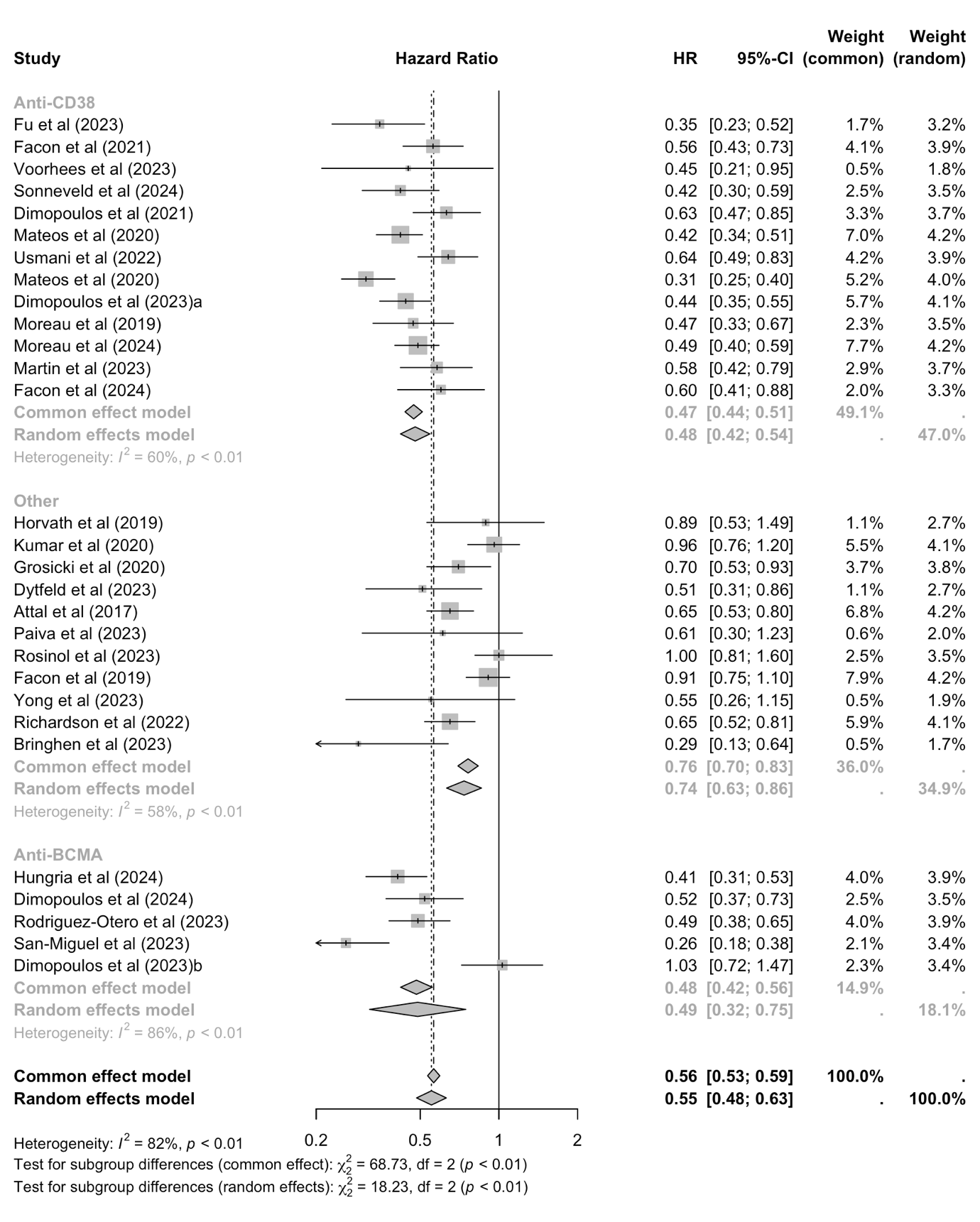


**Figure S7.** PFS (HR) pooled-estimate for the base-case analysis (by adjustment)


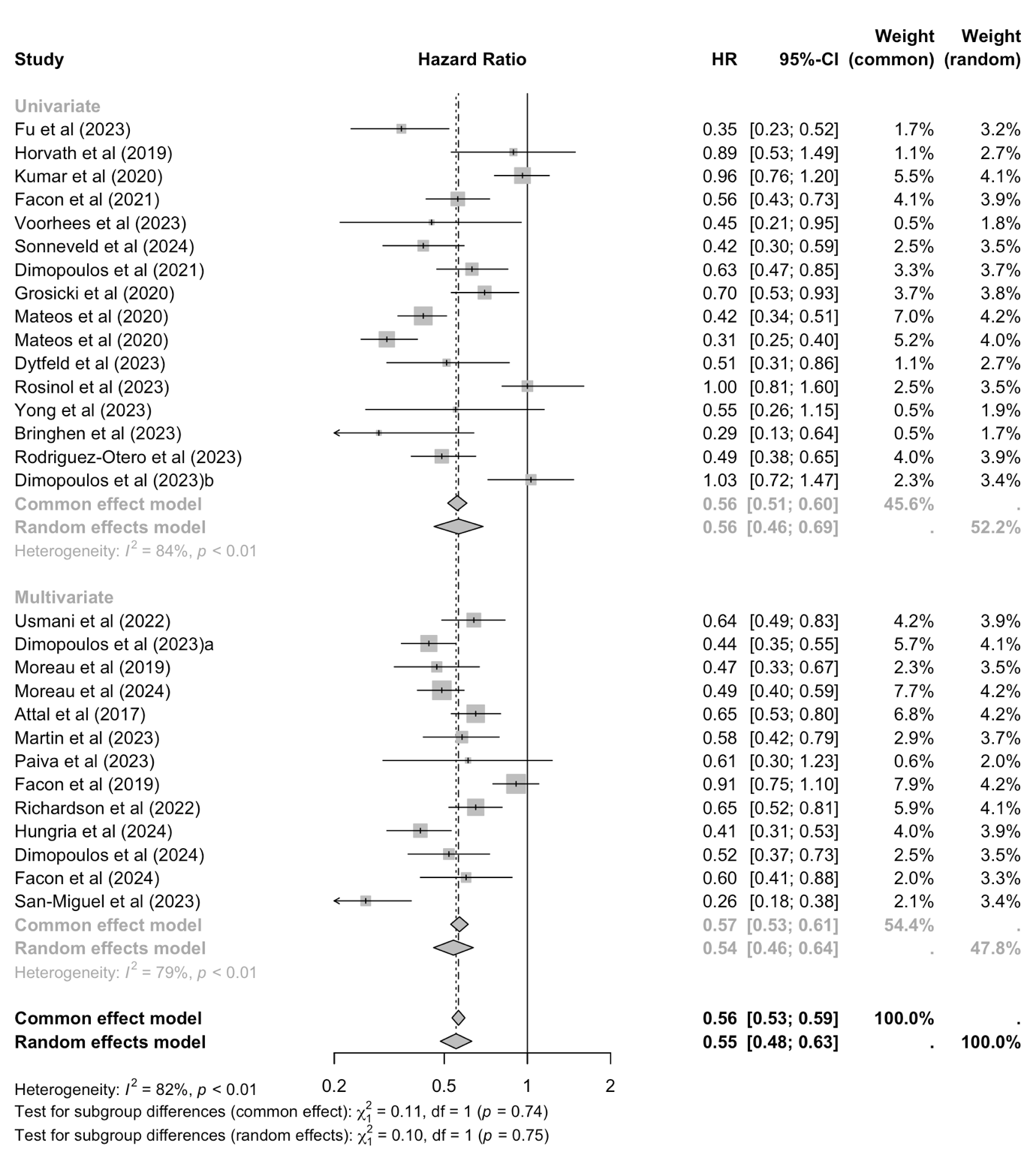


**Figure S8.** OS (HR) pooled-estimate for the base-case analysis (by MM setting)


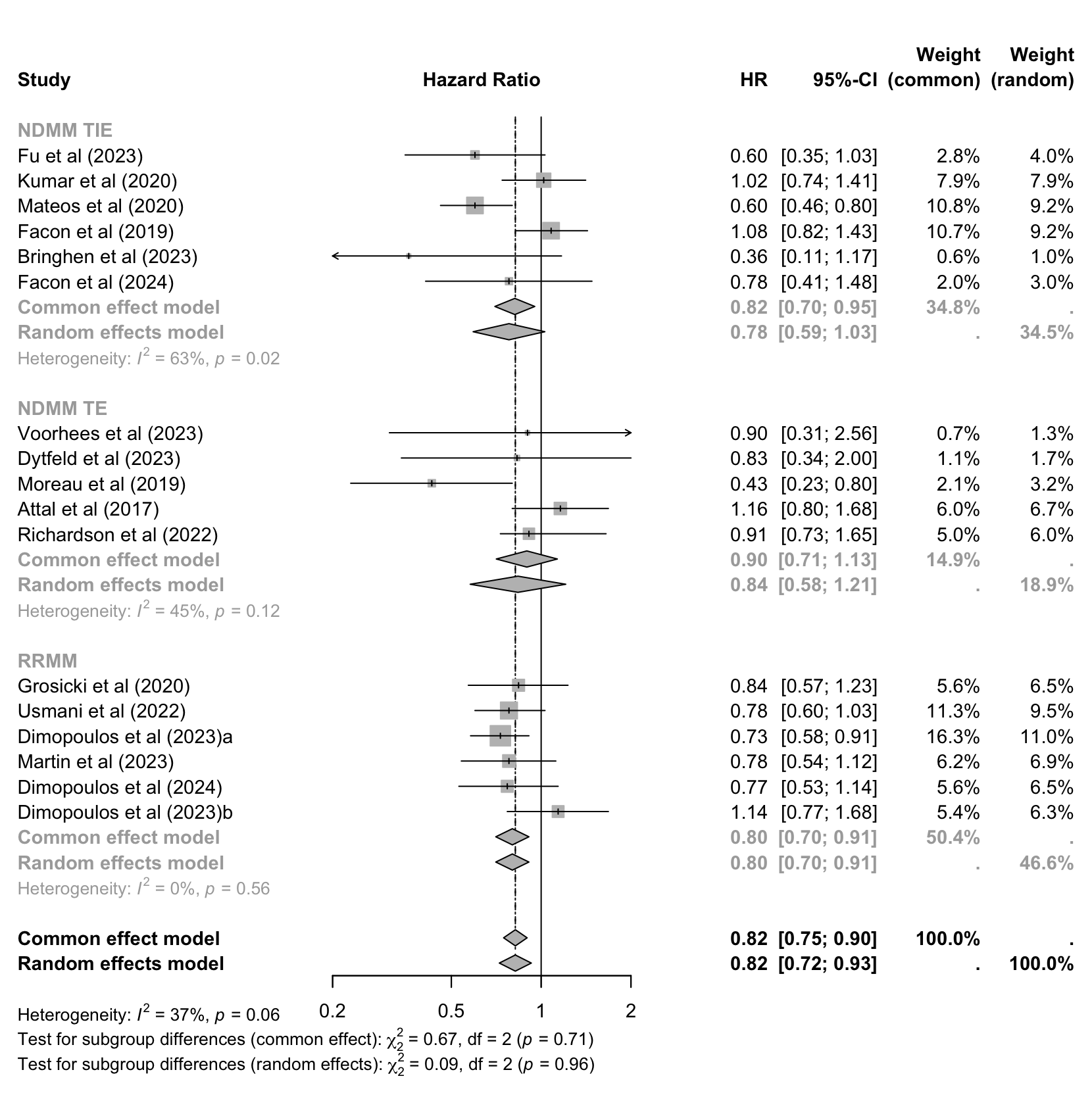


**Figure S9.** OS (HR) pooled-estimate for the base-case analysis (by region)


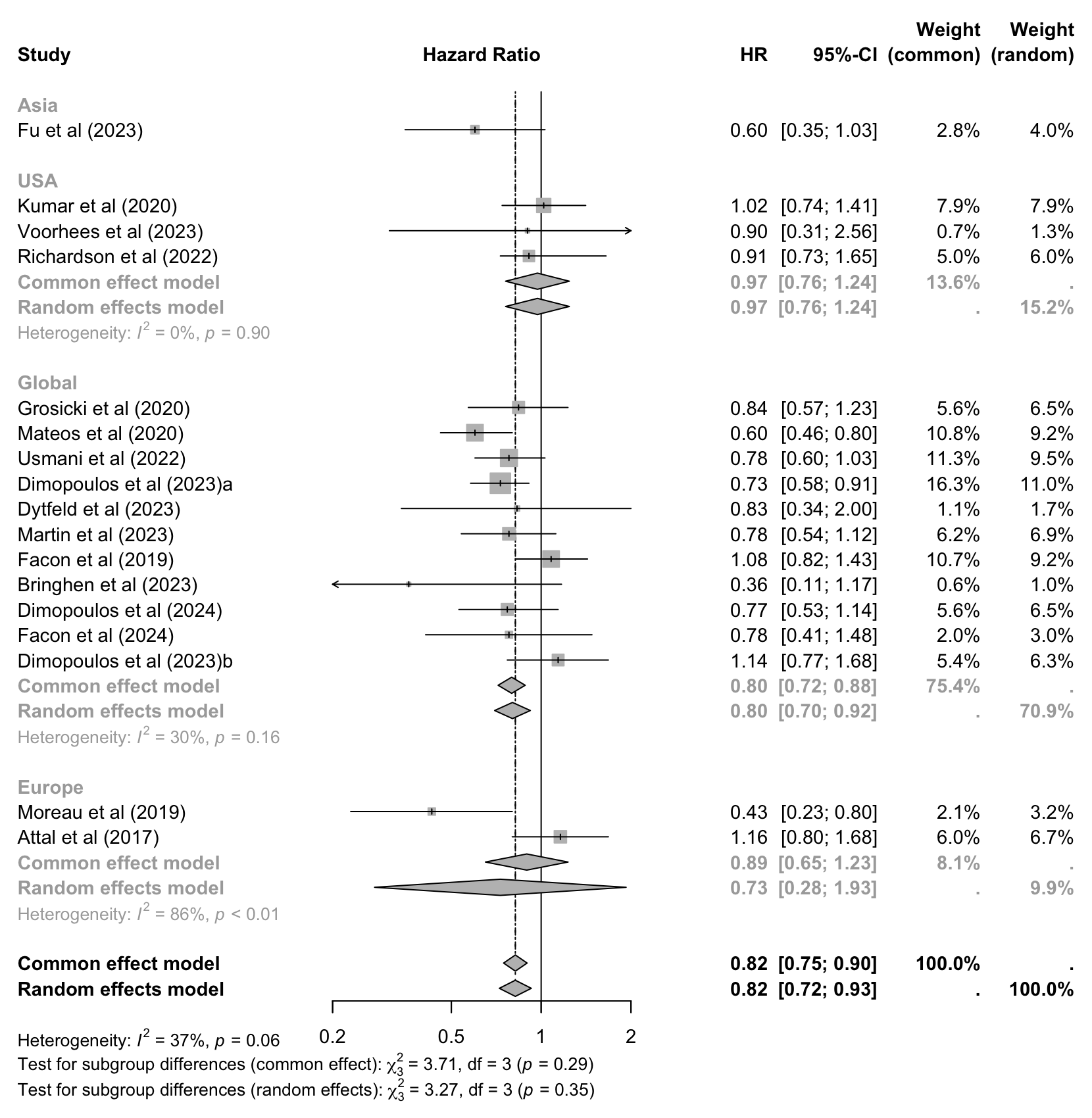


**Figure S10.** OS (HR) pooled-estimate for the base-case analysis (by follow-up)


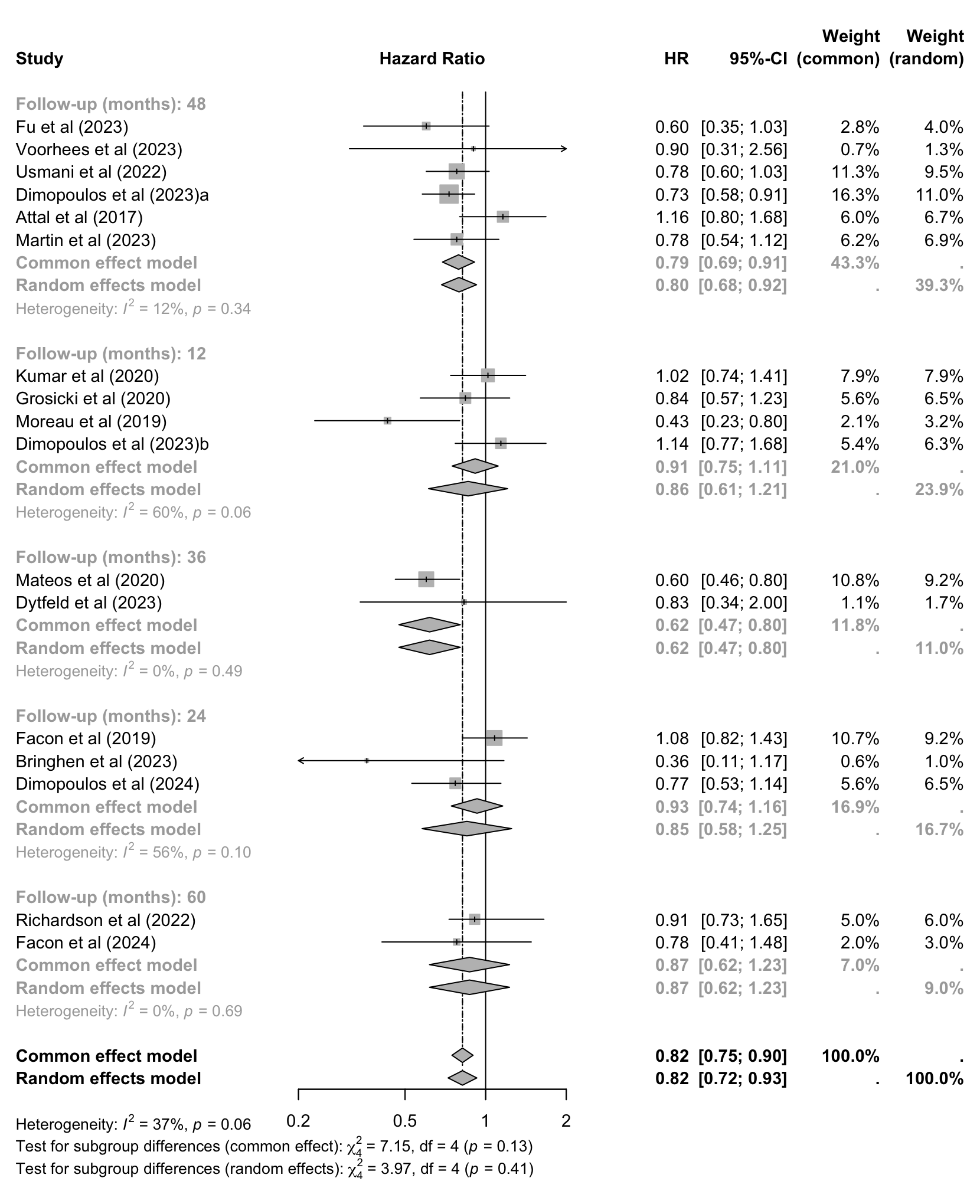


**Figure S11.** OS (HR) pooled-estimate for the base-case analysis (by adjustment)


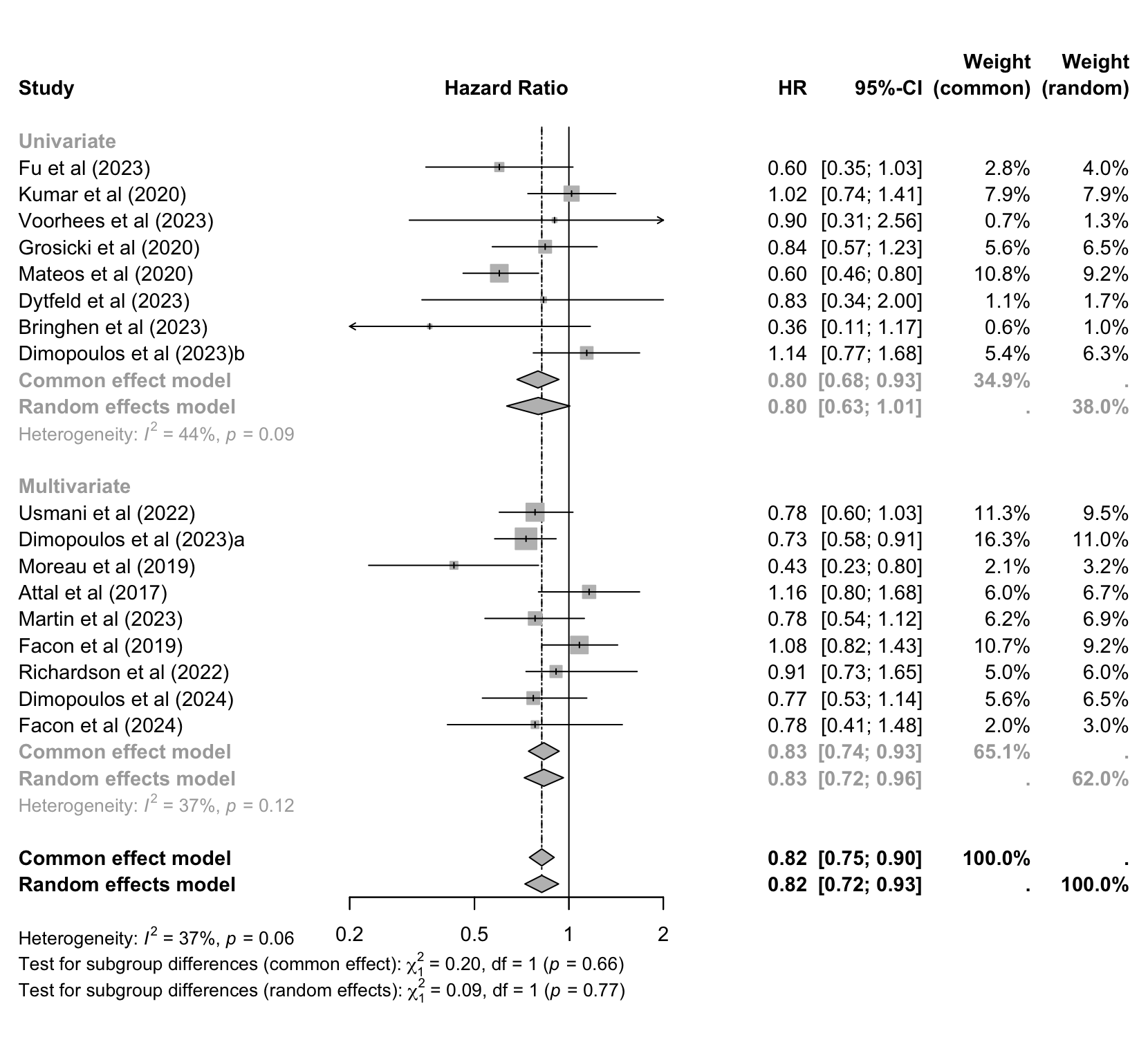


**Figure S12.**

**
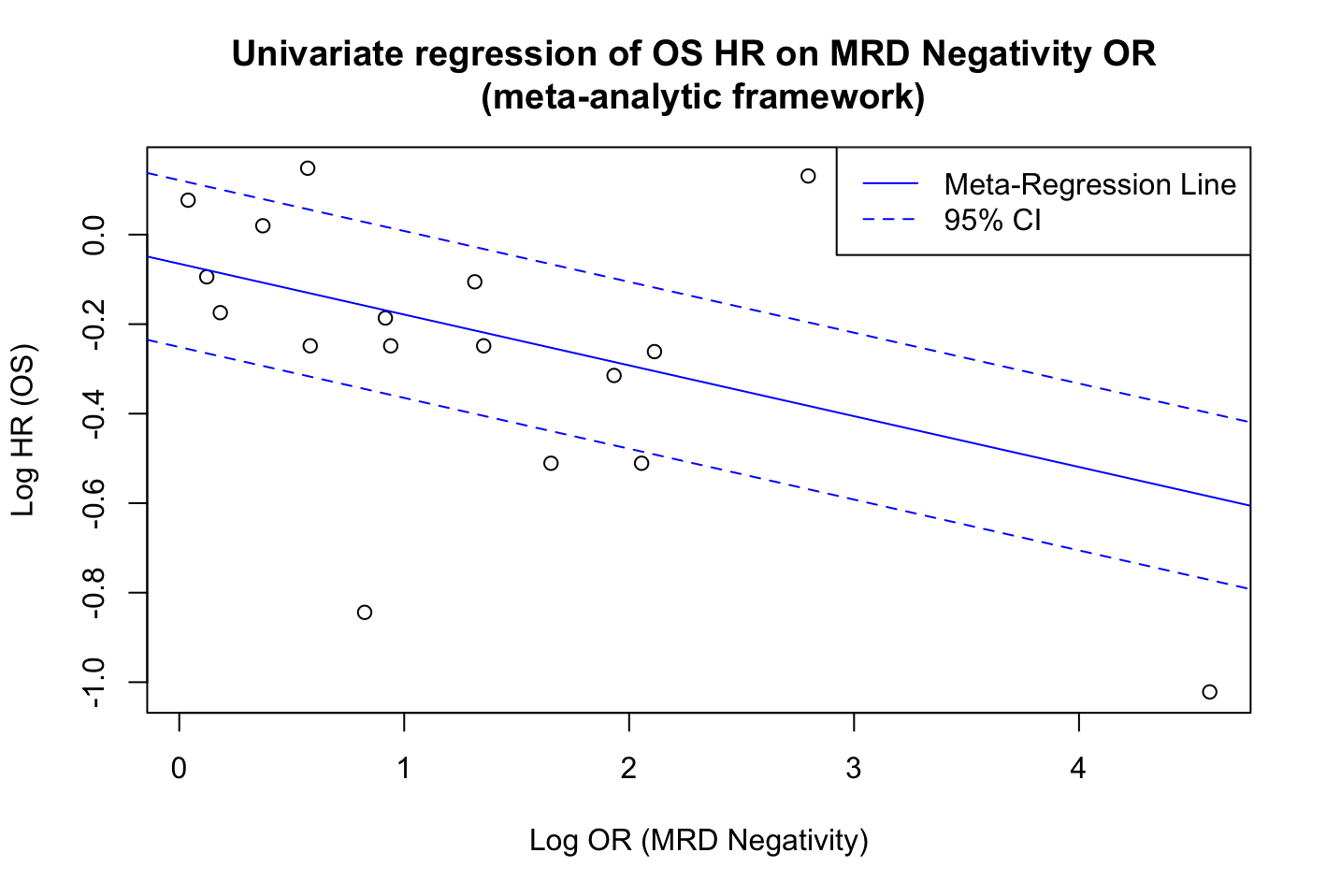
**

**Figure S13.** MRD positive (RD) pooled-estimate for the base-case analysis (by MM setting)


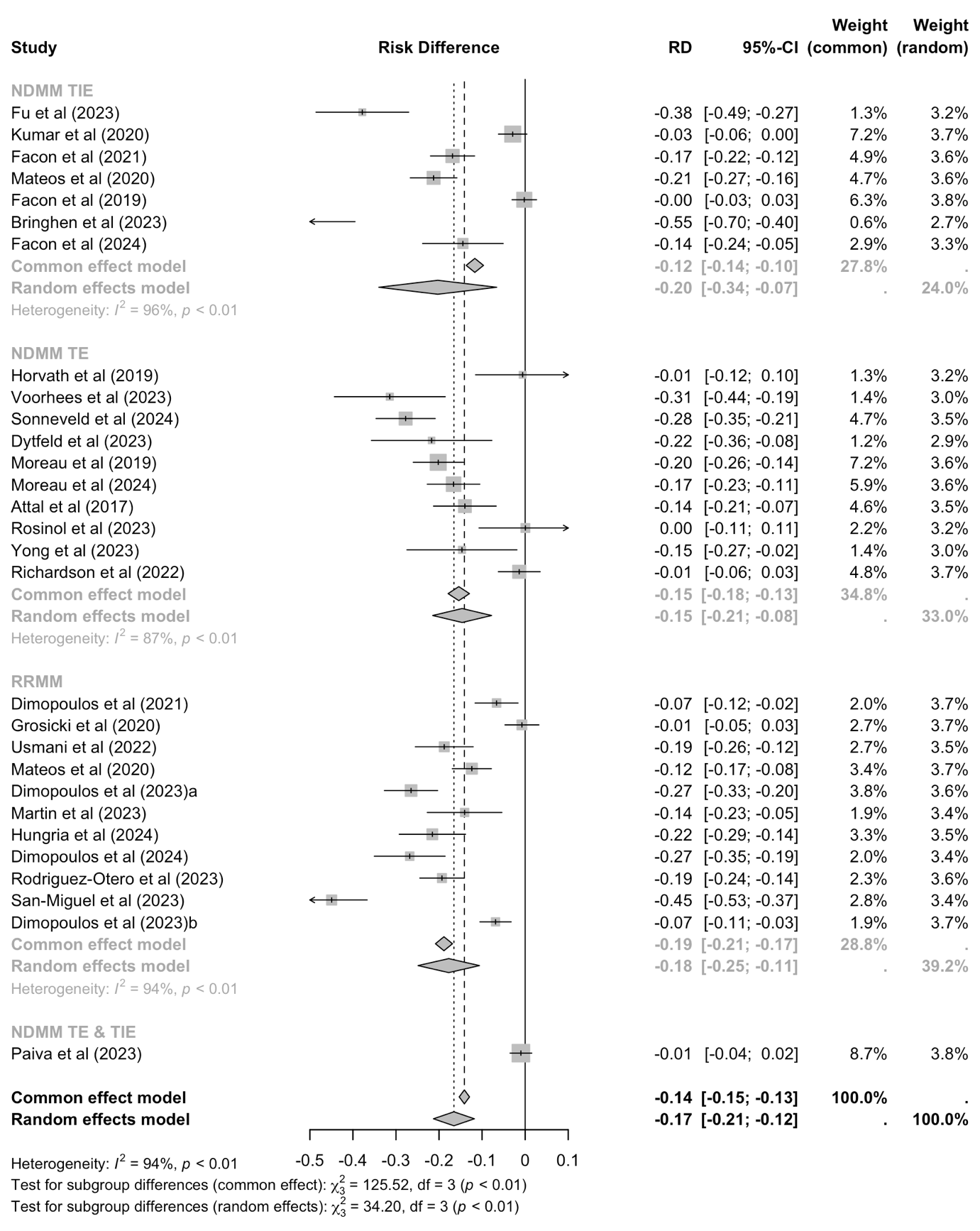


**Figure S14.** MRD positive (RD) pooled-estimate for the base-case analysis (by region)


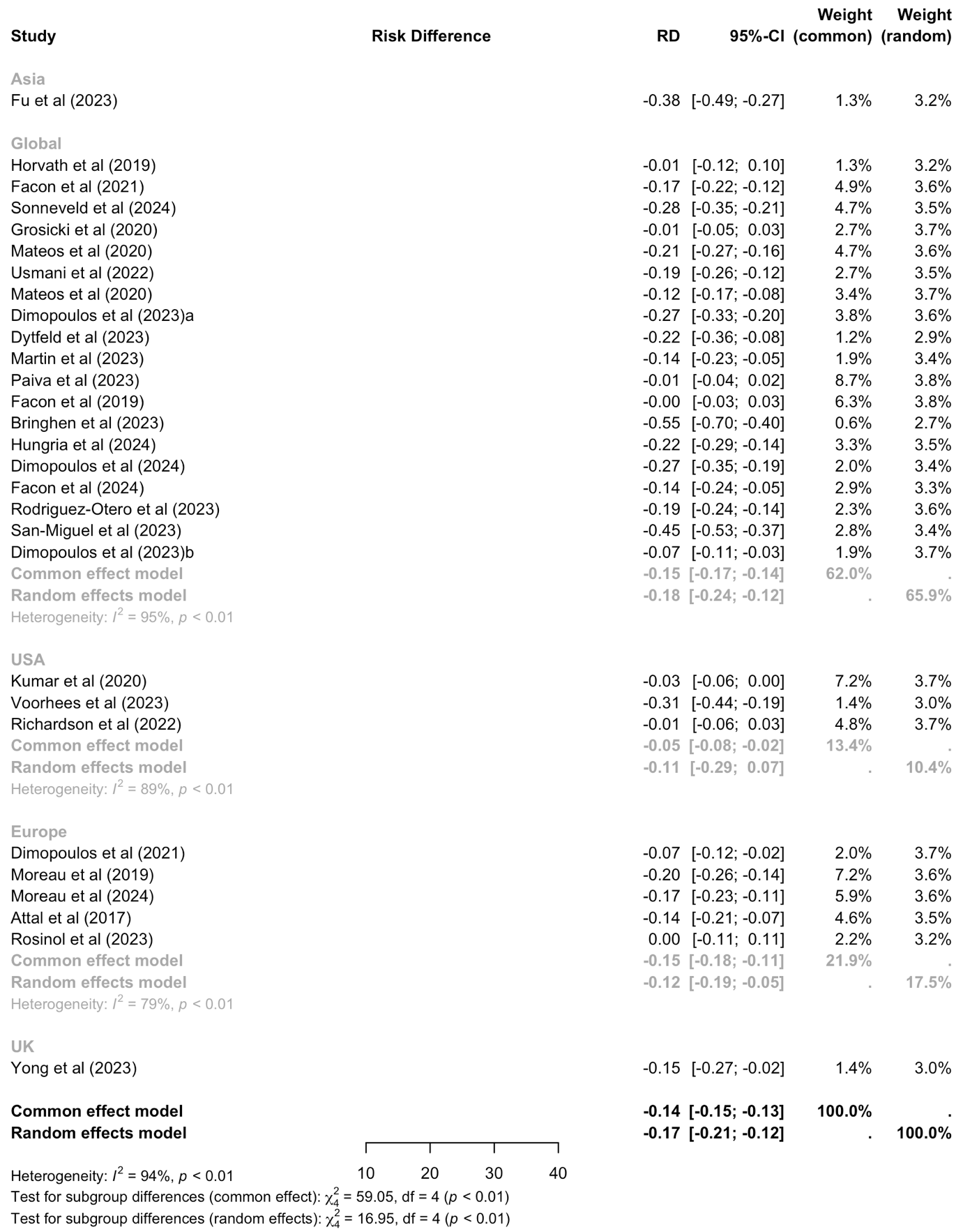


**Figure S15.** MRD positive (RD) pooled-estimate for the base-case analysis (by treatment)


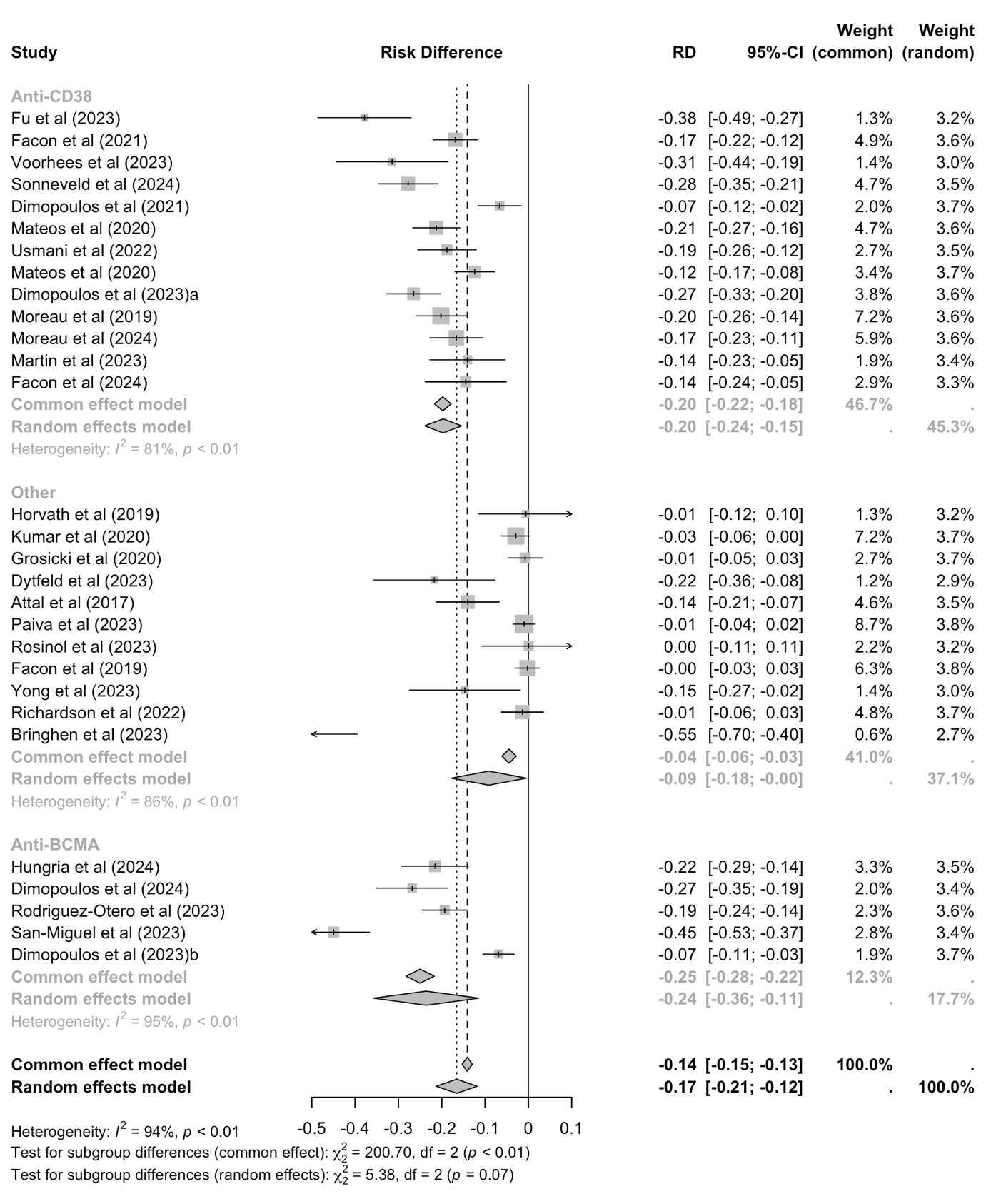


**Figure S16.** MRD positive (RD) pooled-estimate for the base-case analysis (by follow-up)

**
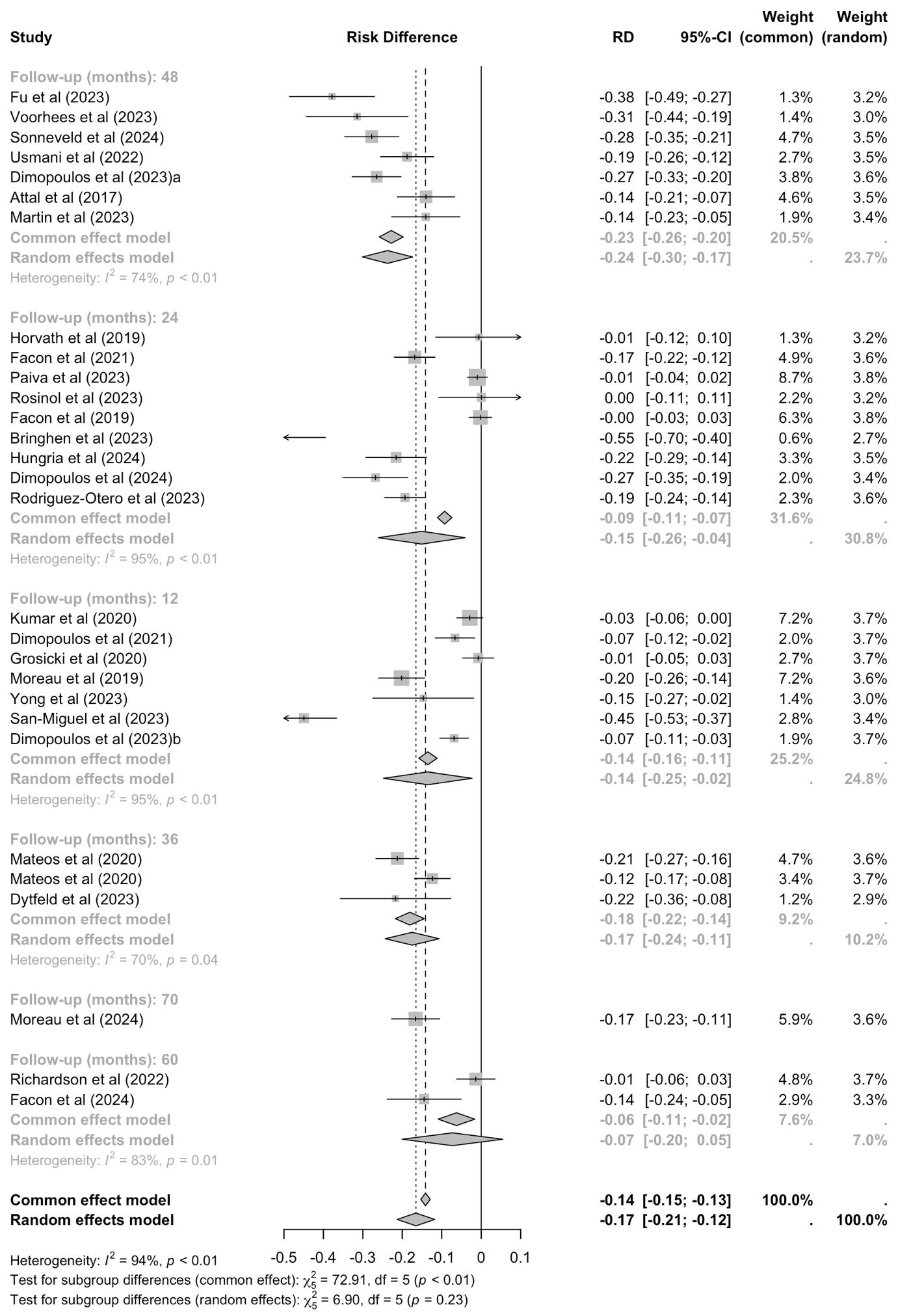
**

**Figure S17.**

**
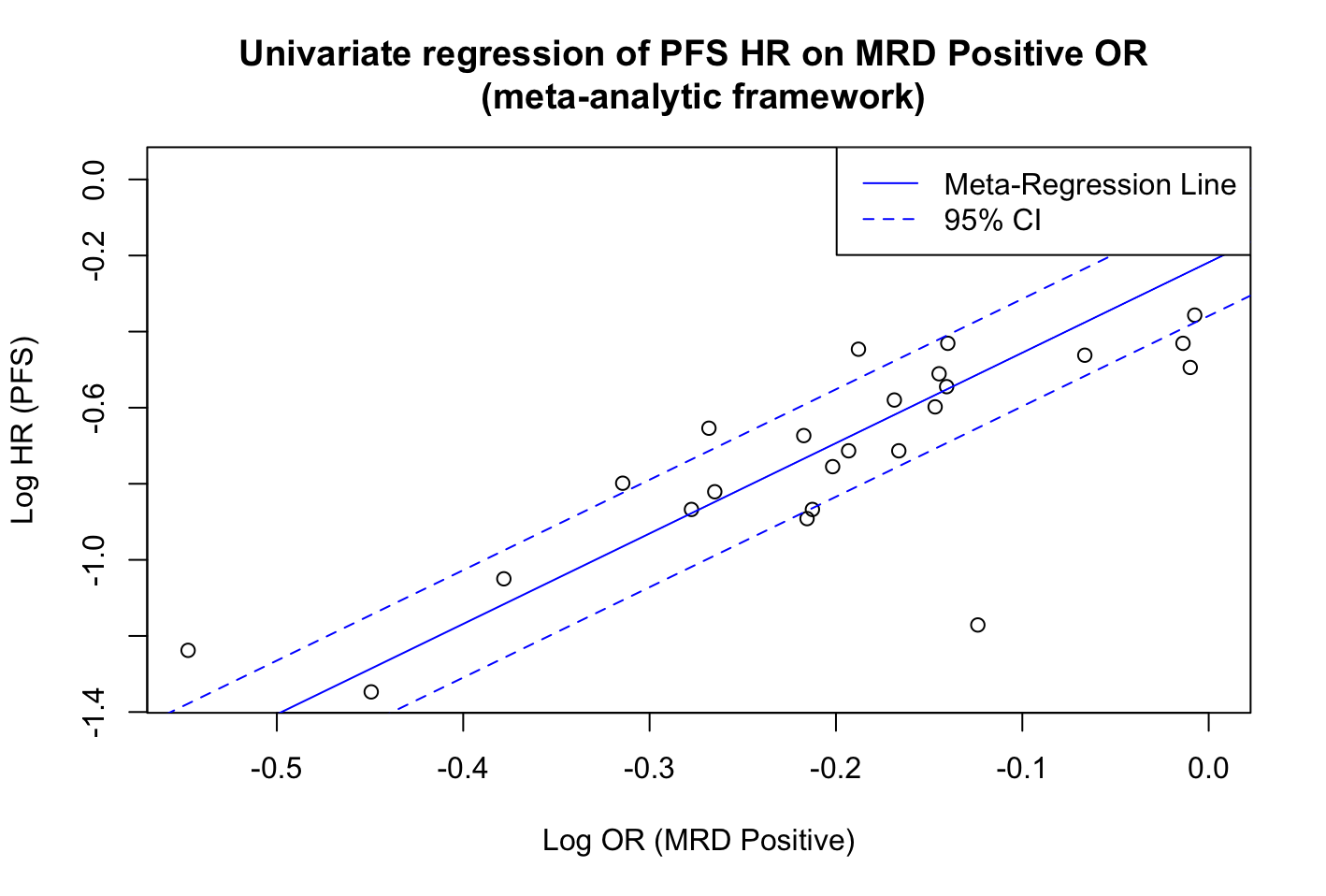
**

**Figure S18.**

**
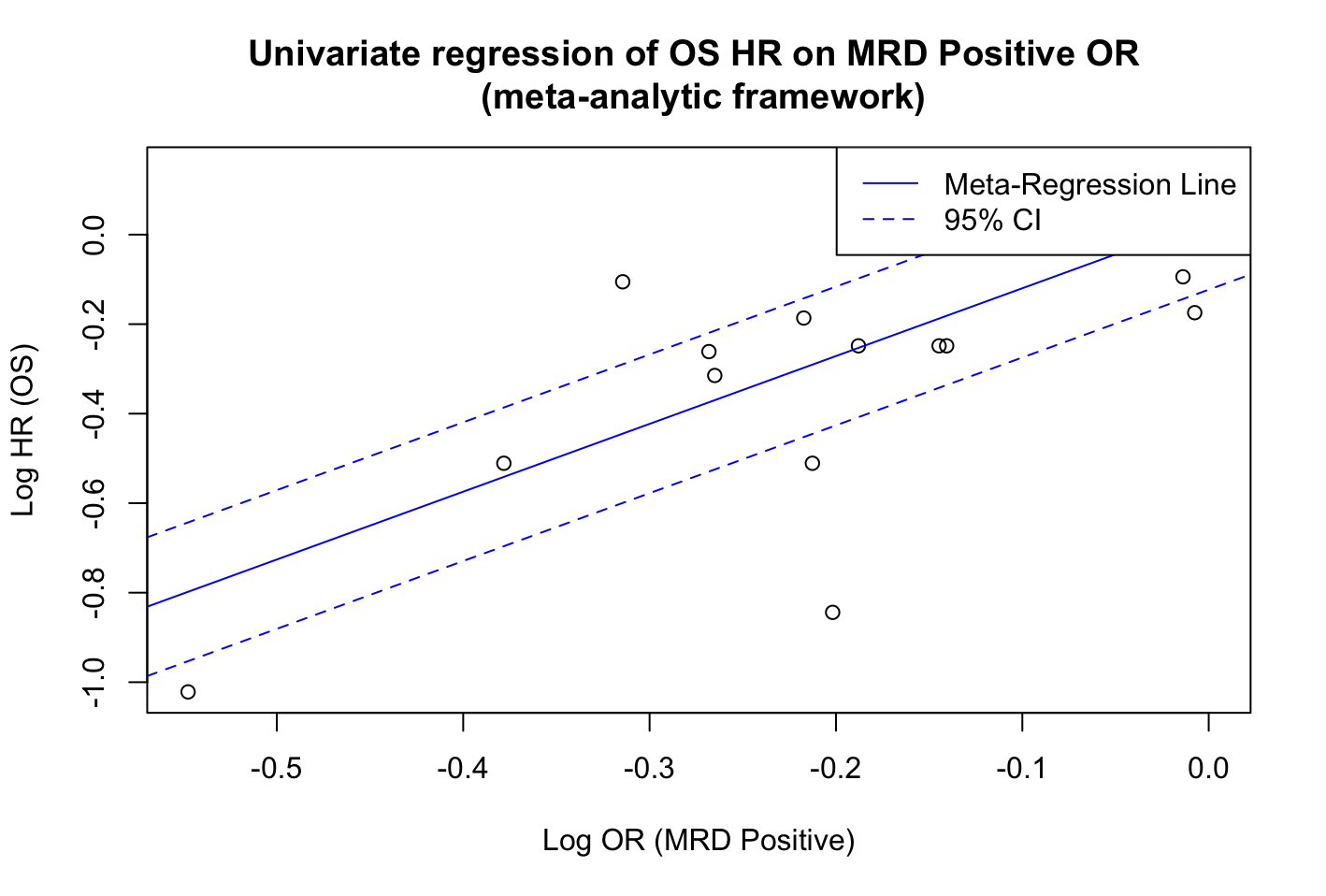
**

**Figure S19.** Funnel plot for MRD negativity ORs


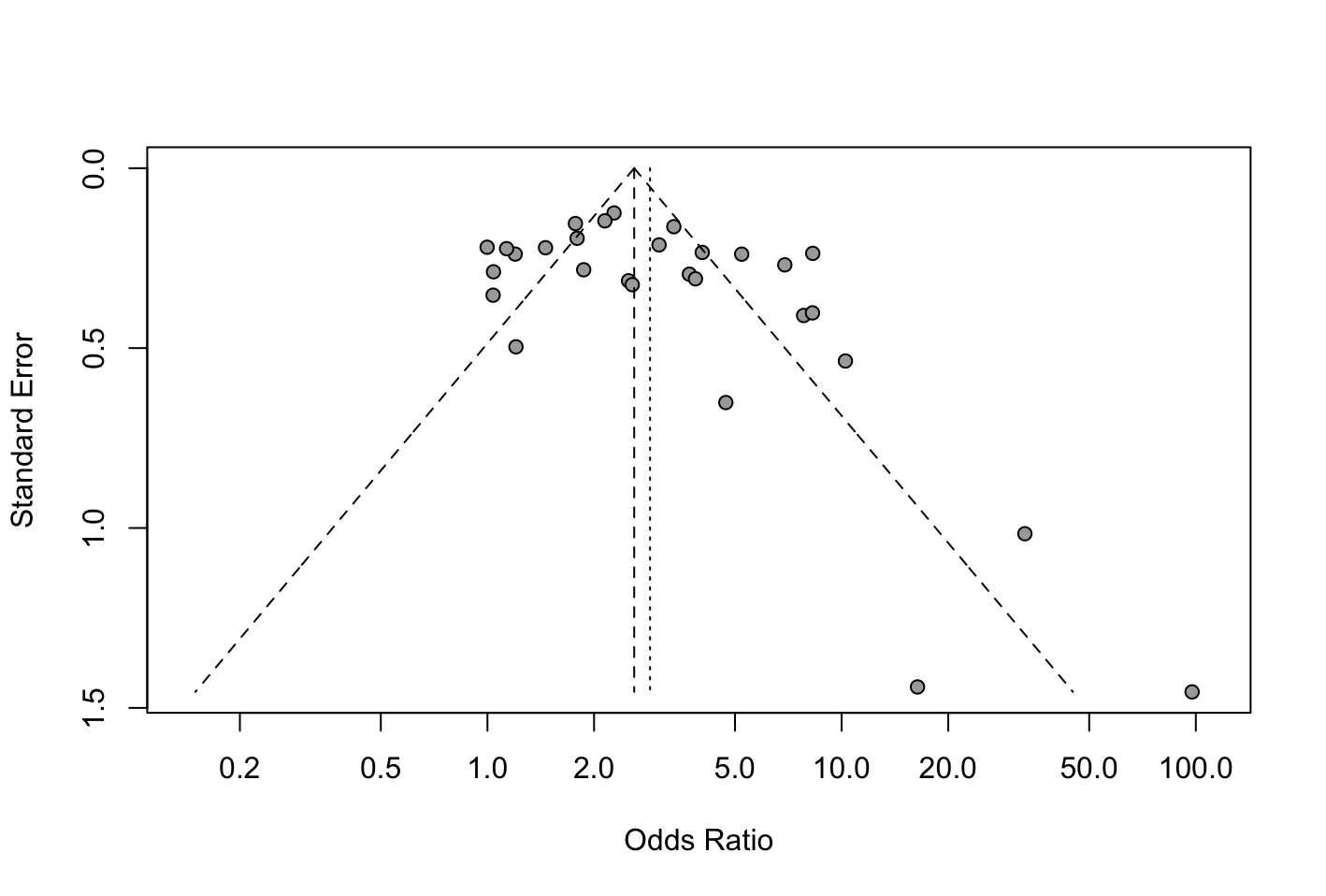


**Figure S20.** Funnel plot for PFS HRs


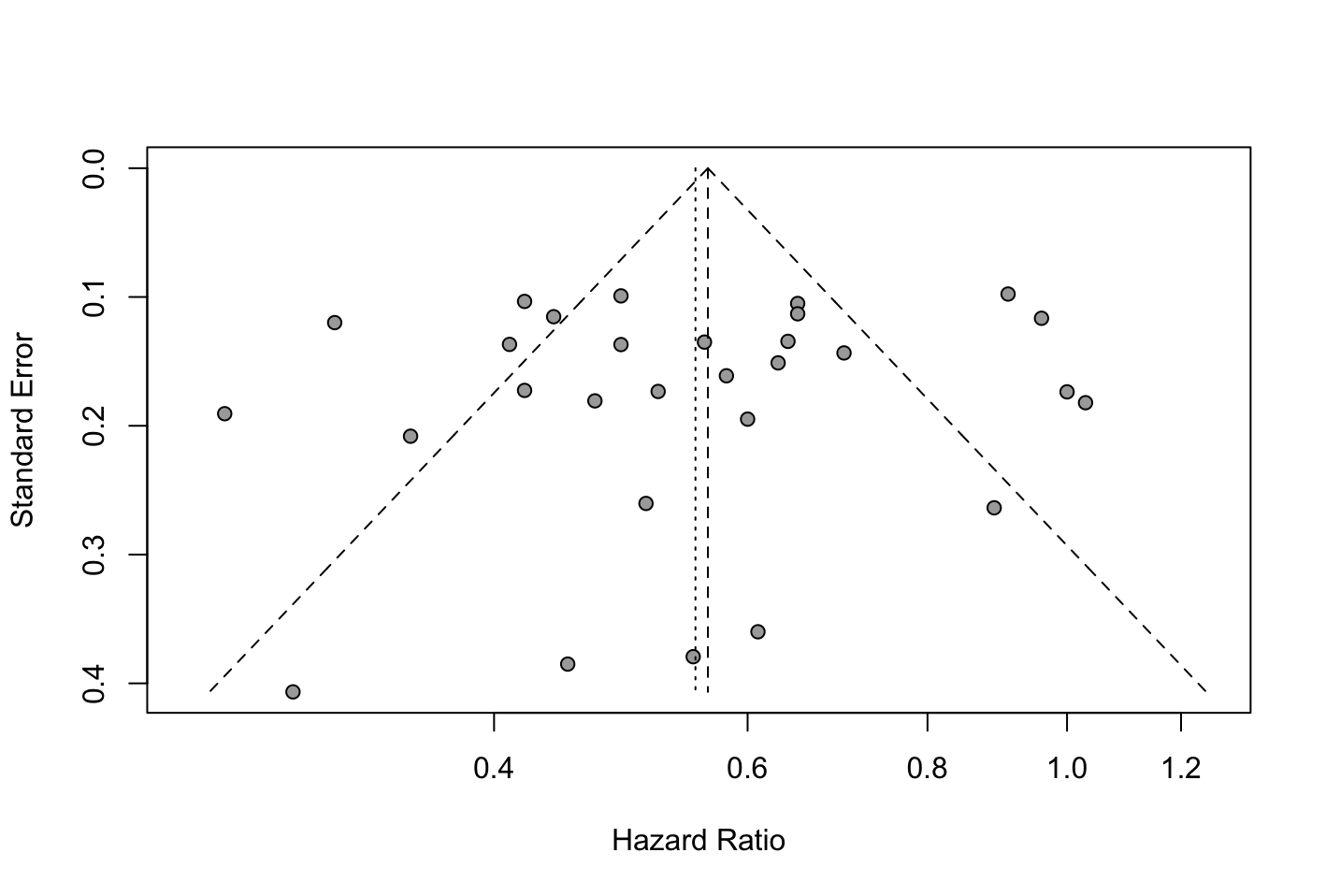


**Figure S21.** Funnel plot for OS HRs


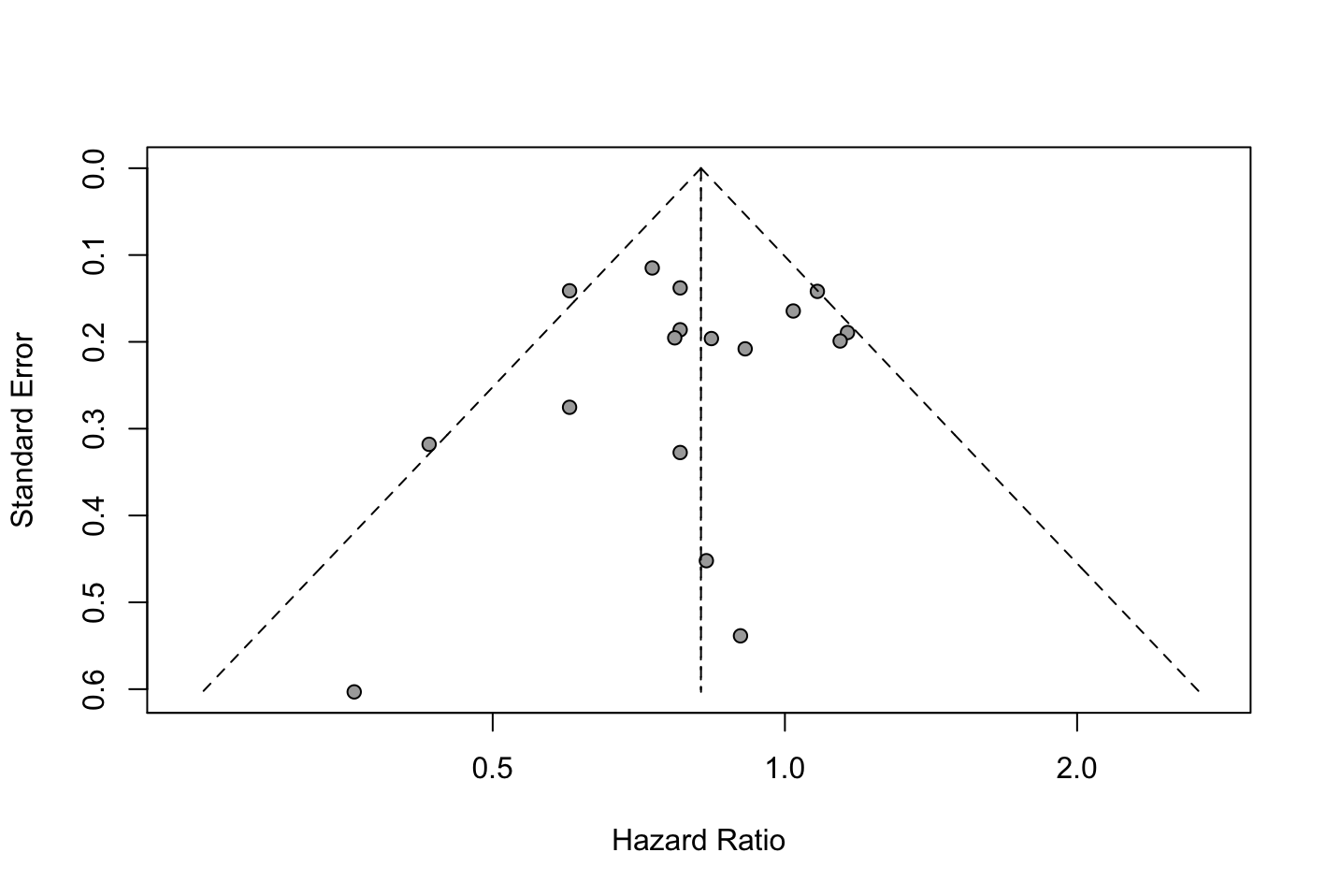


**Figure S22.** Funnel plot for MRD positive RDs


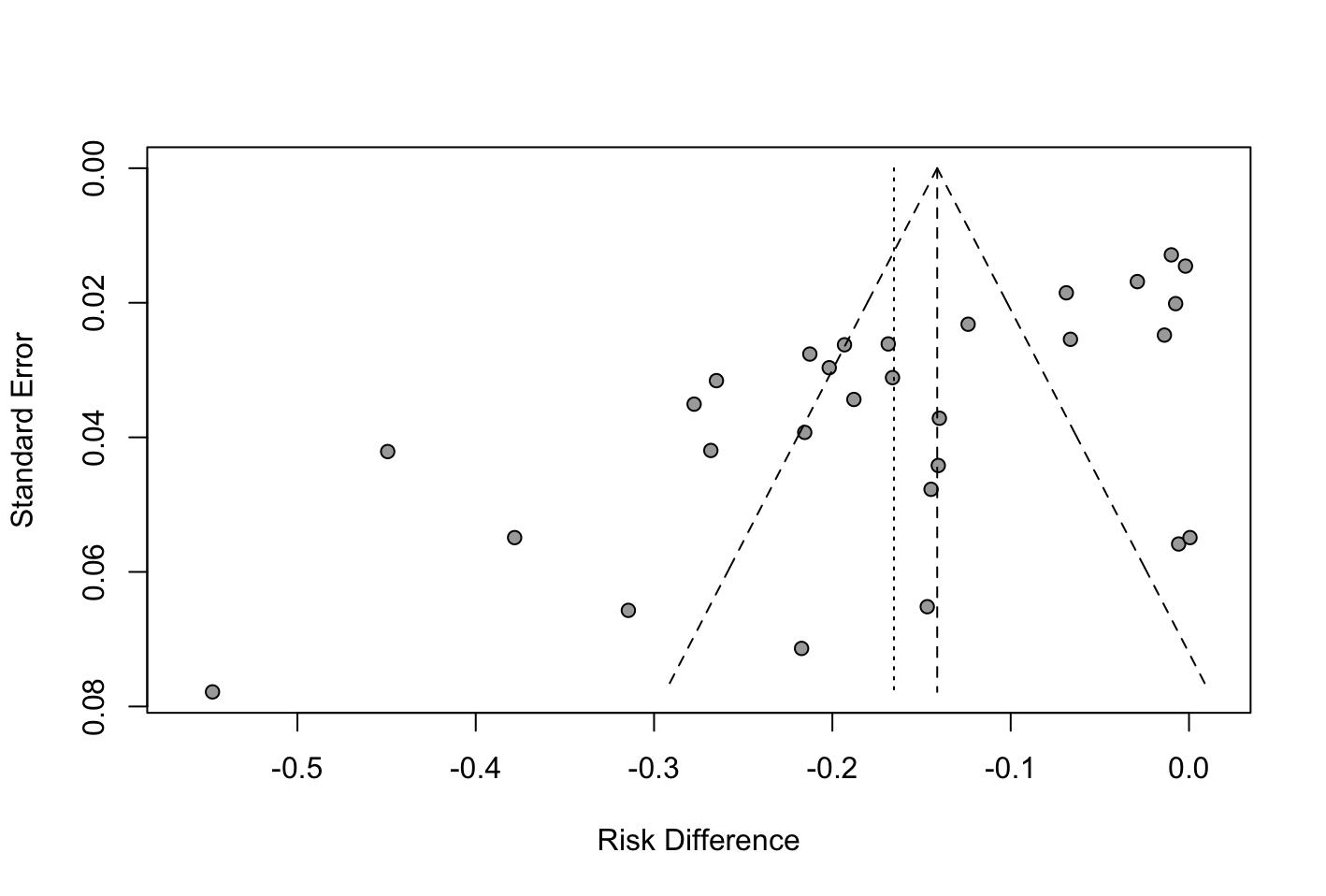


*1-year follow-up meta-analysis*

**Figure S23.** MRD negativity (OR) pooled-estimate (by MM setting)


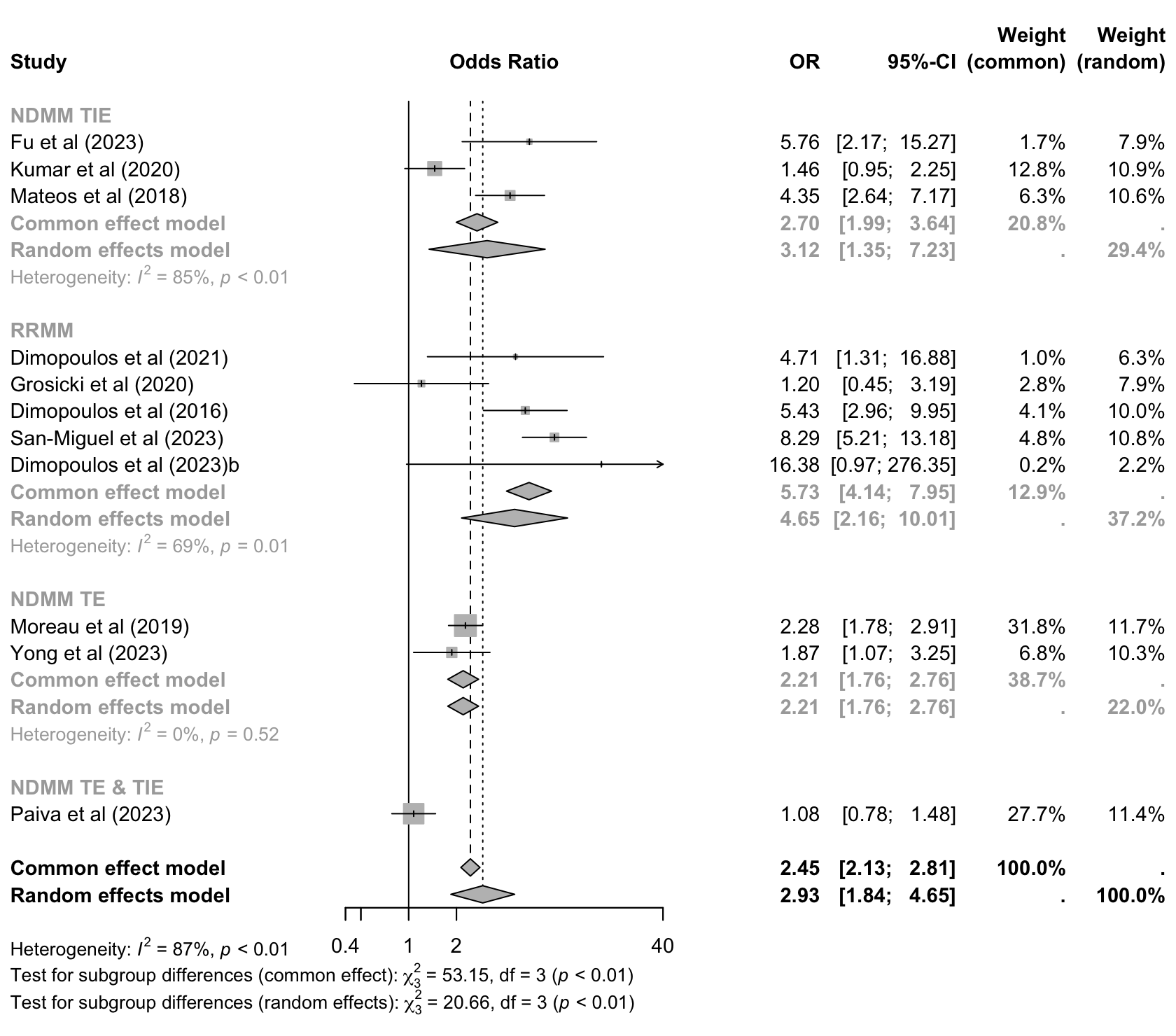


**Figure S24.** MRD negativity (OR) pooled-estimate (by region)


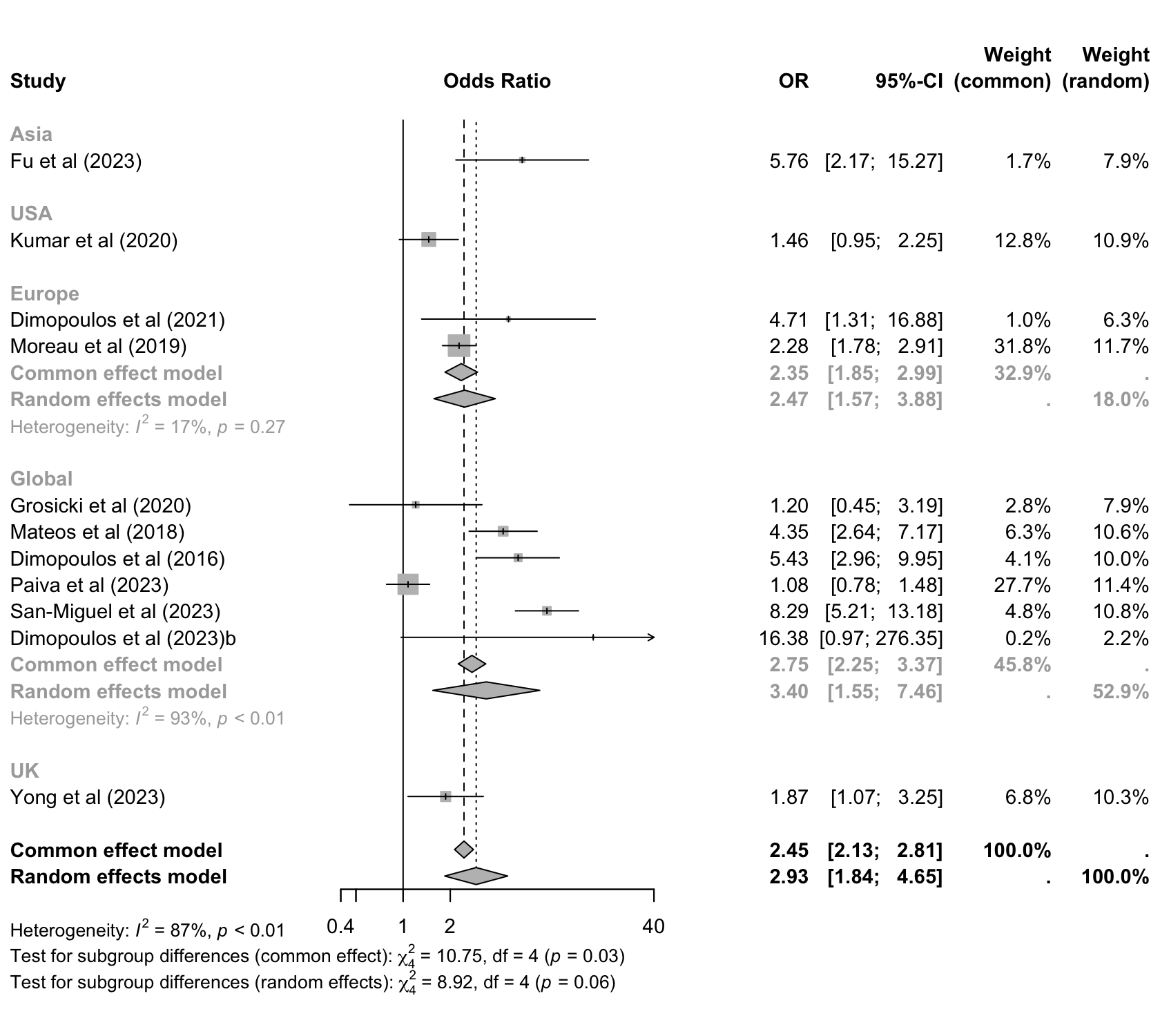


**Figure S25.** MRD negativity (OR) pooled-estimate (by treatment)

**
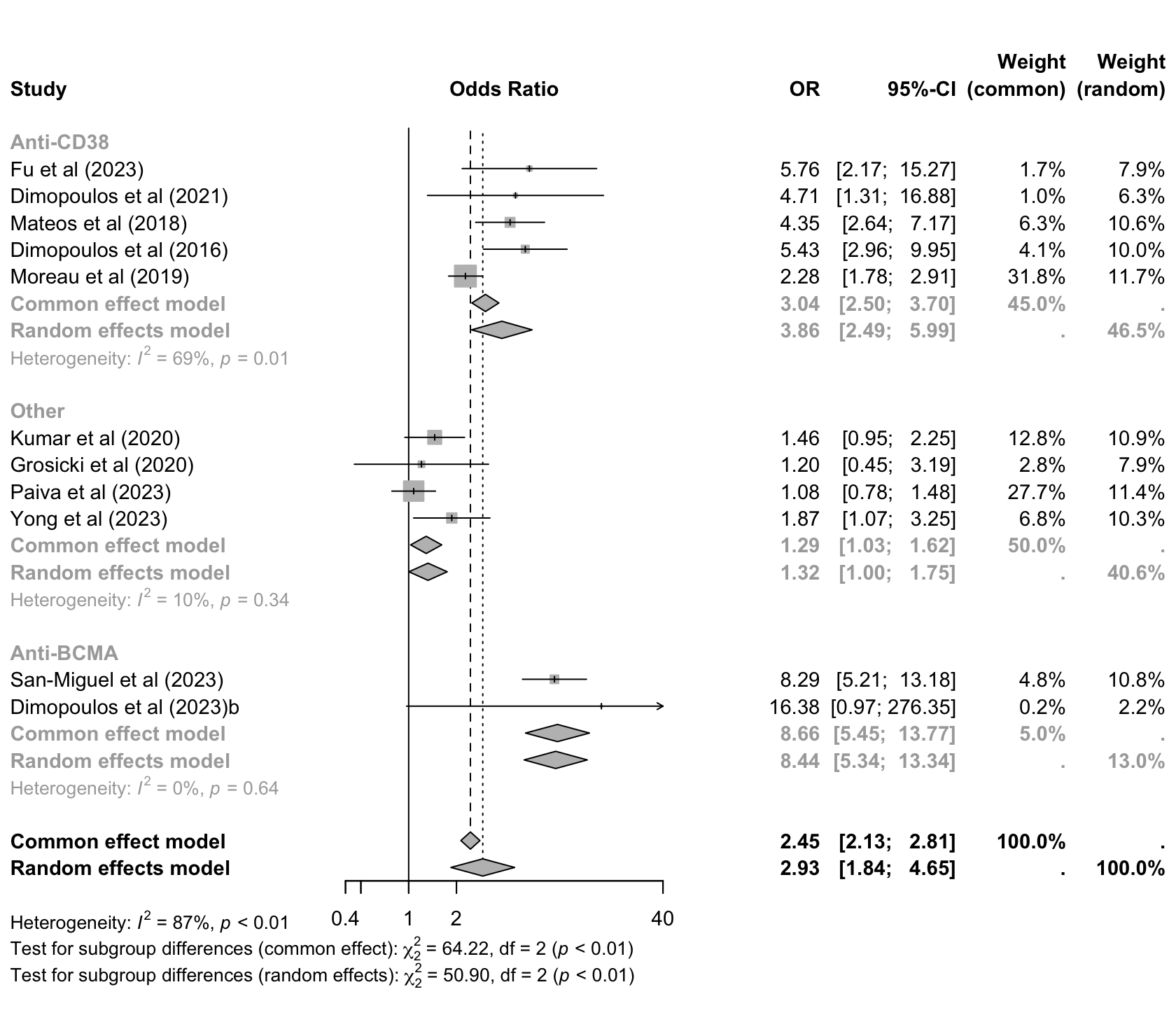
**

**Figure S26.** PFS (HR) pooled-estimate (by MM setting)


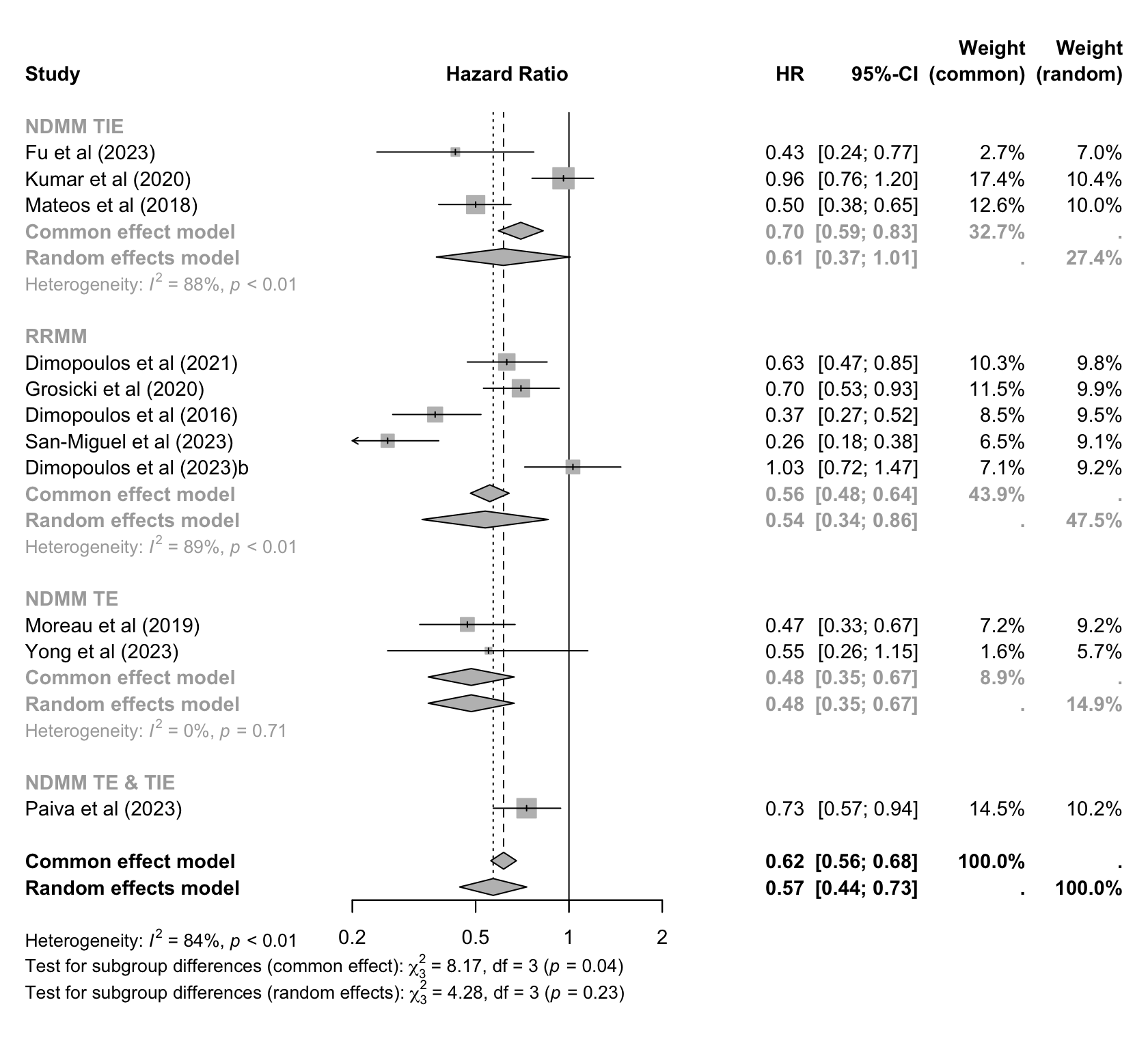


**Figure S27.** PFS (HR) pooled-estimate (by treatment)


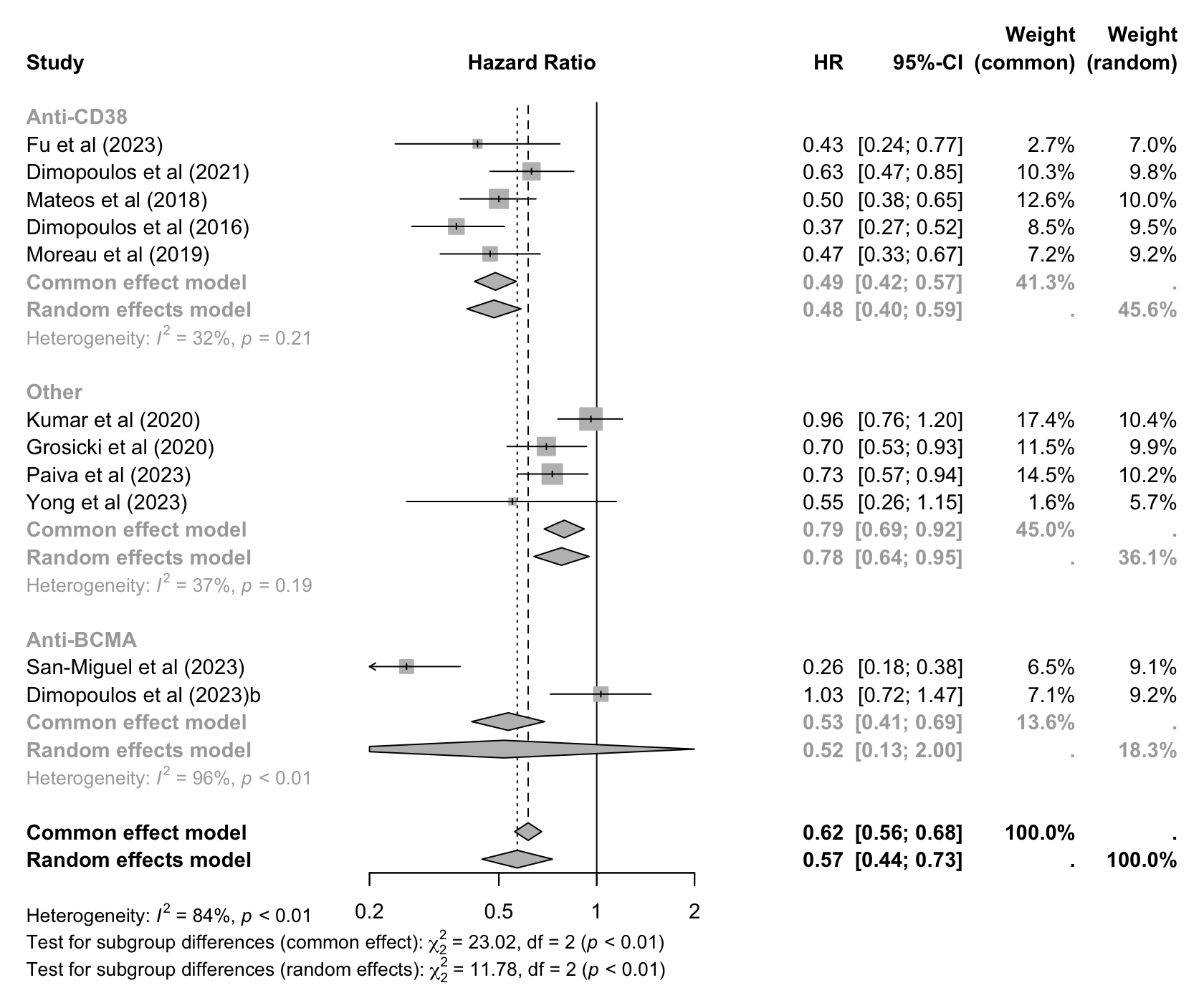


**Figure S28.** PFS (HR) pooled-estimate (by adjustment)


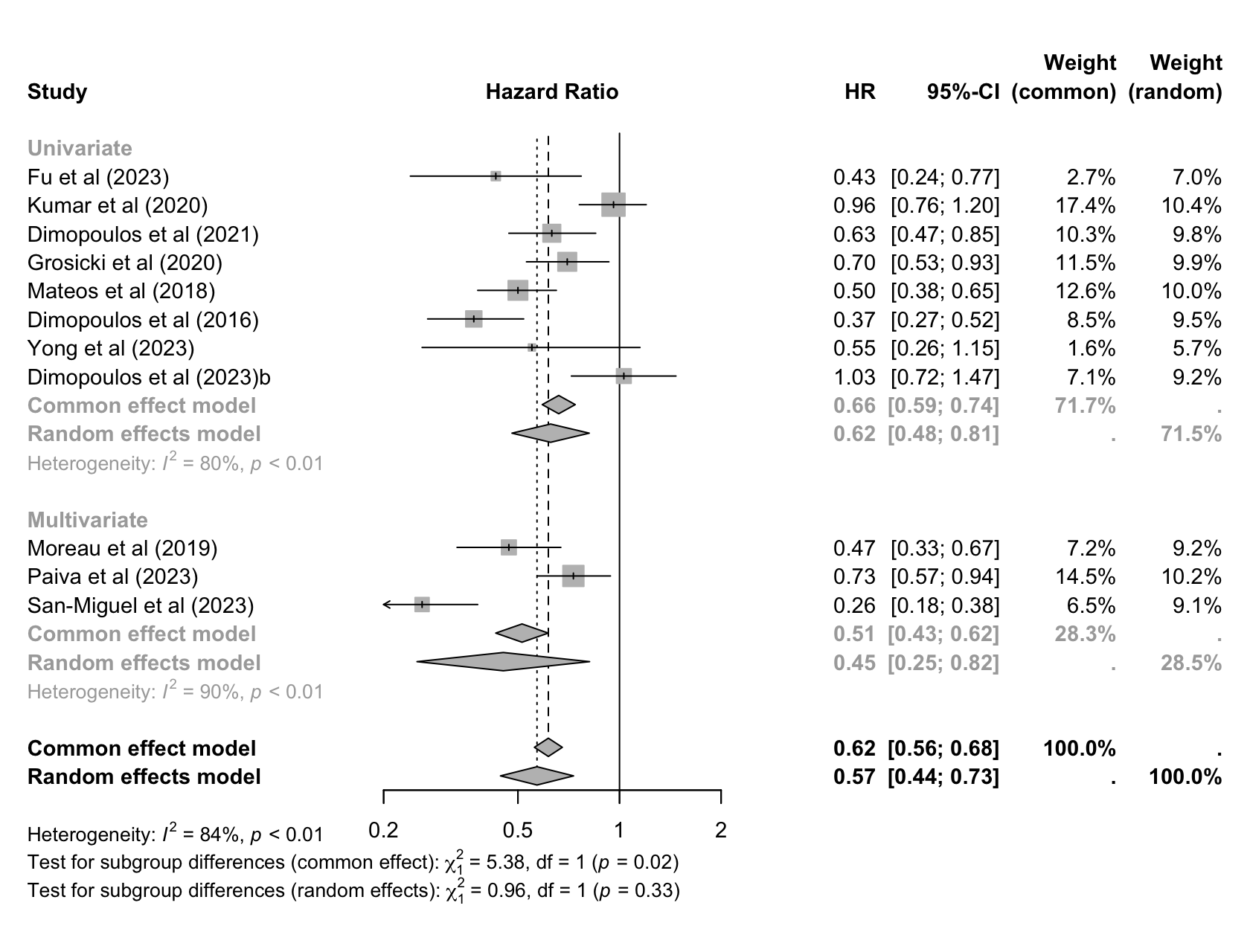


**Figure S29.** OS (HR) pooled-estimate (by MM setting)


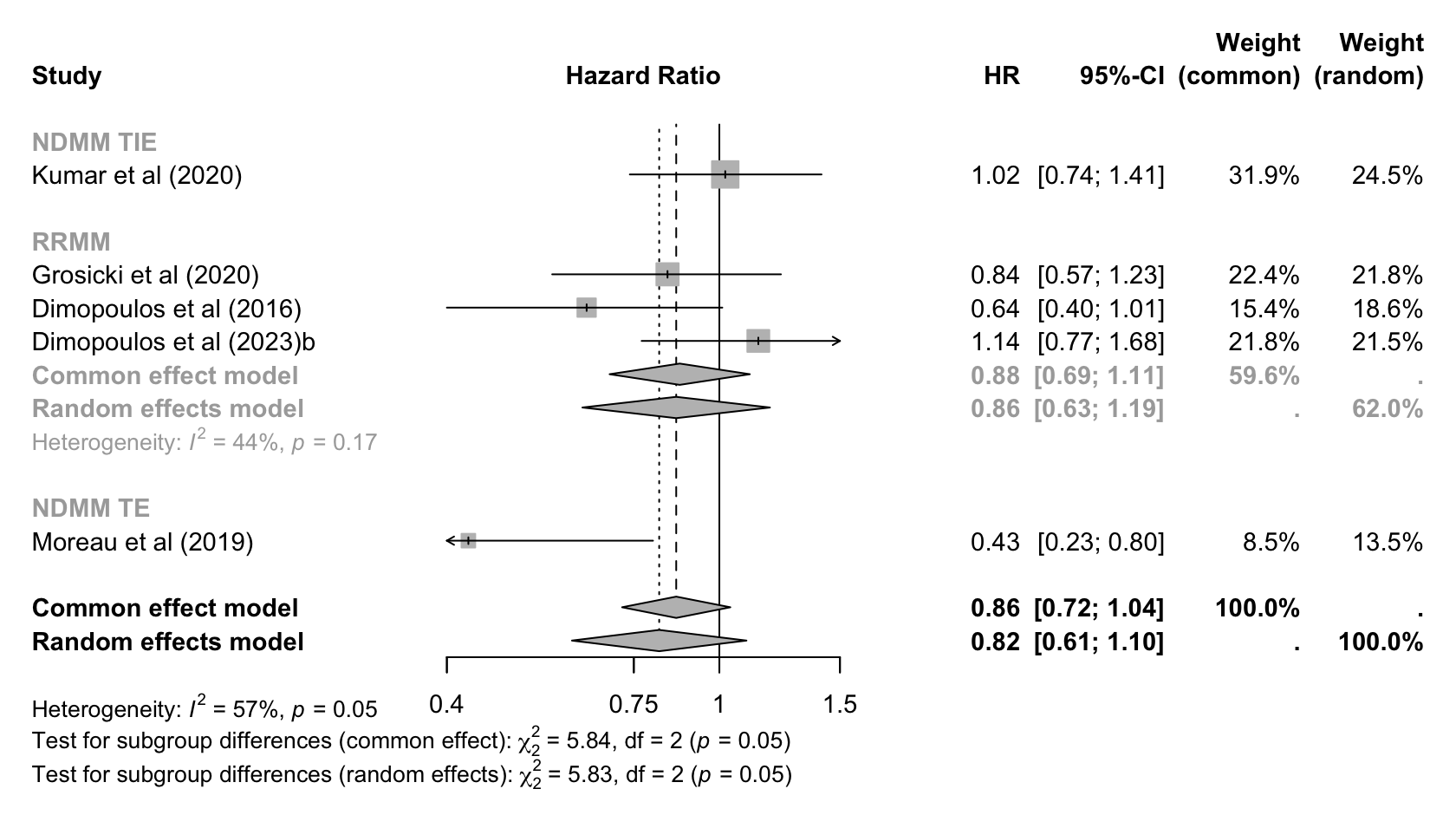


**Figure S30.** OS (HR) pooled-estimate (by region)


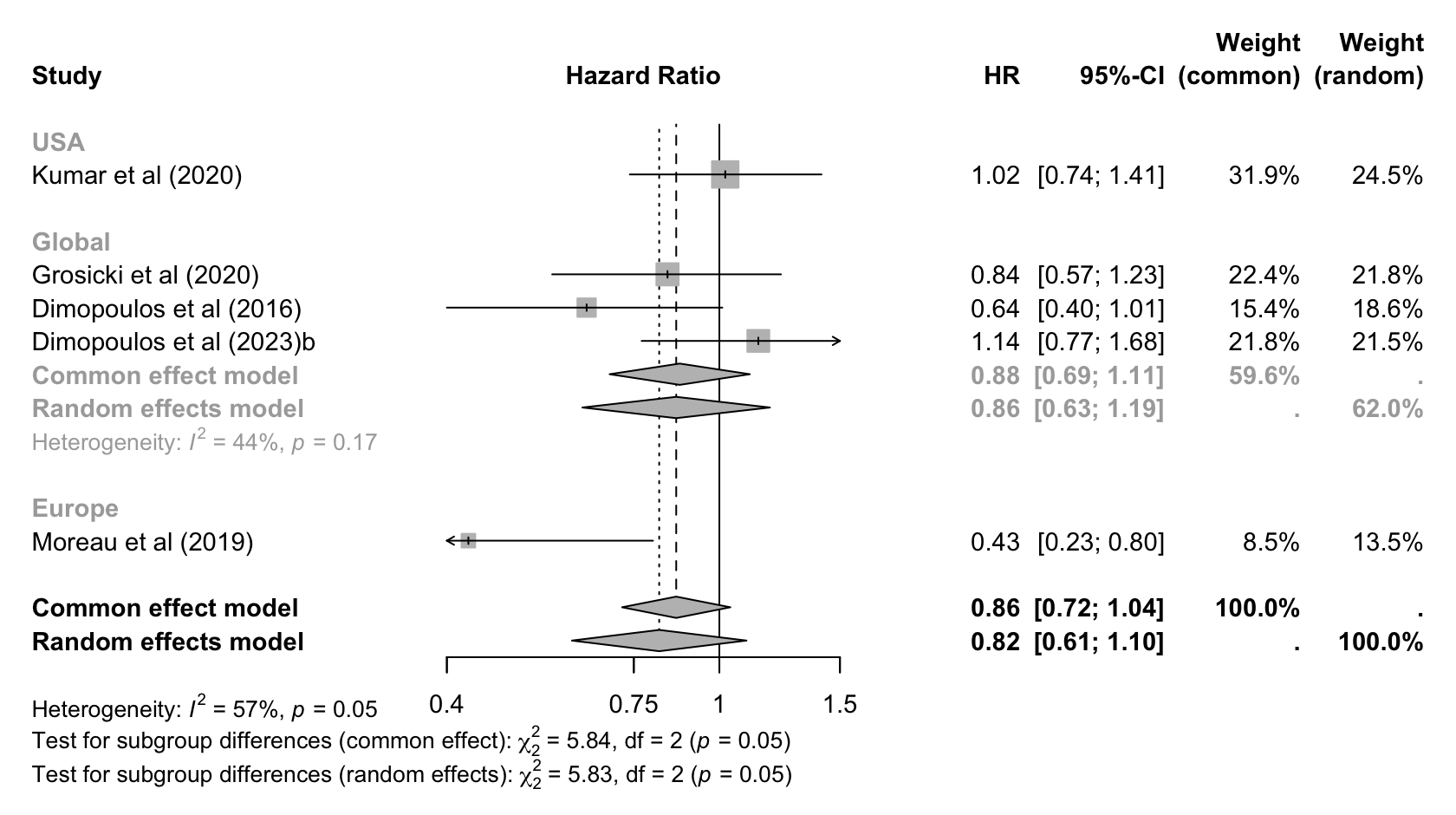


**Figure S31.** OS (HR) pooled-estimate (by treatment)


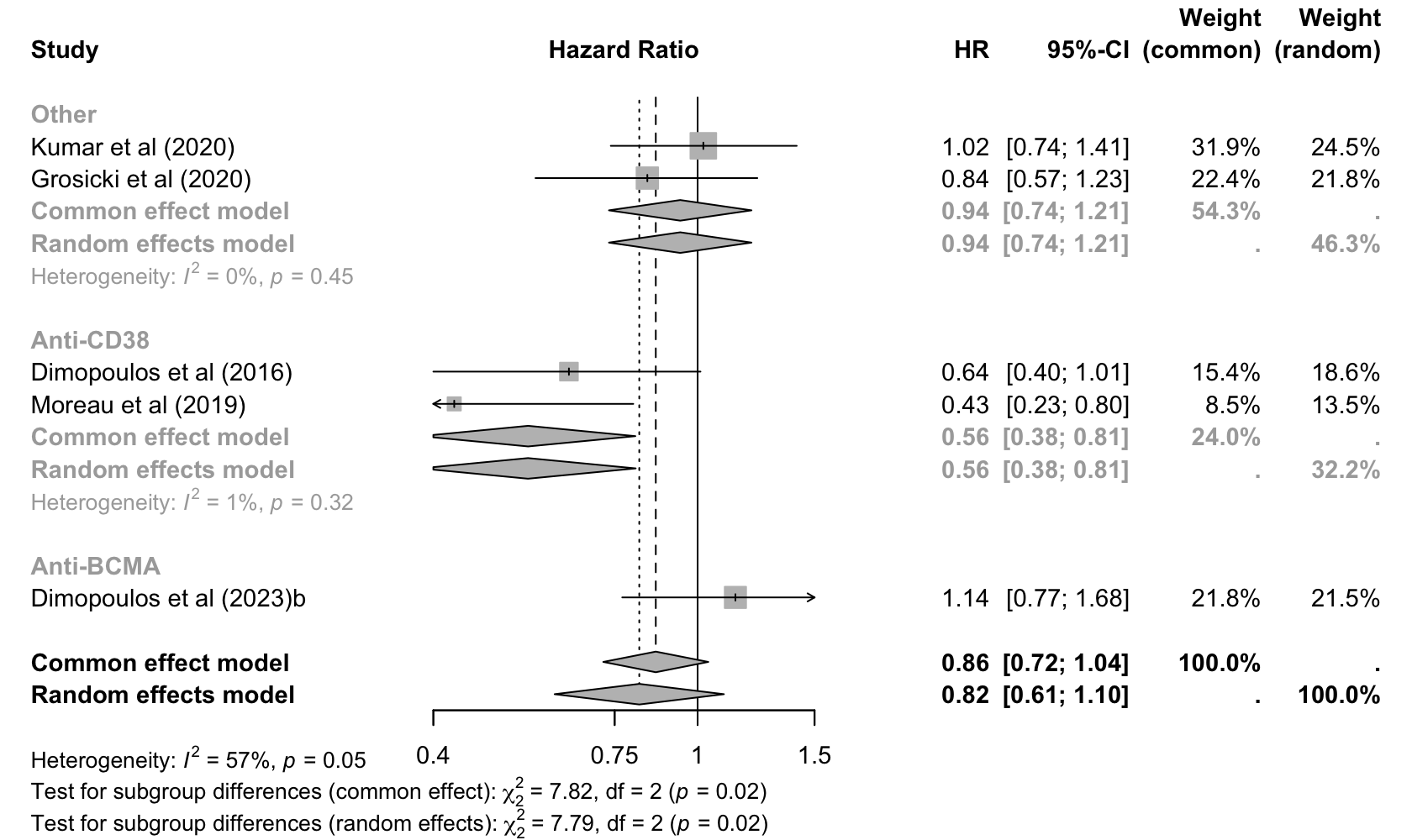


**Figure S32.** OS (HR) pooled-estimate for the base-case analysis (by adjustment)


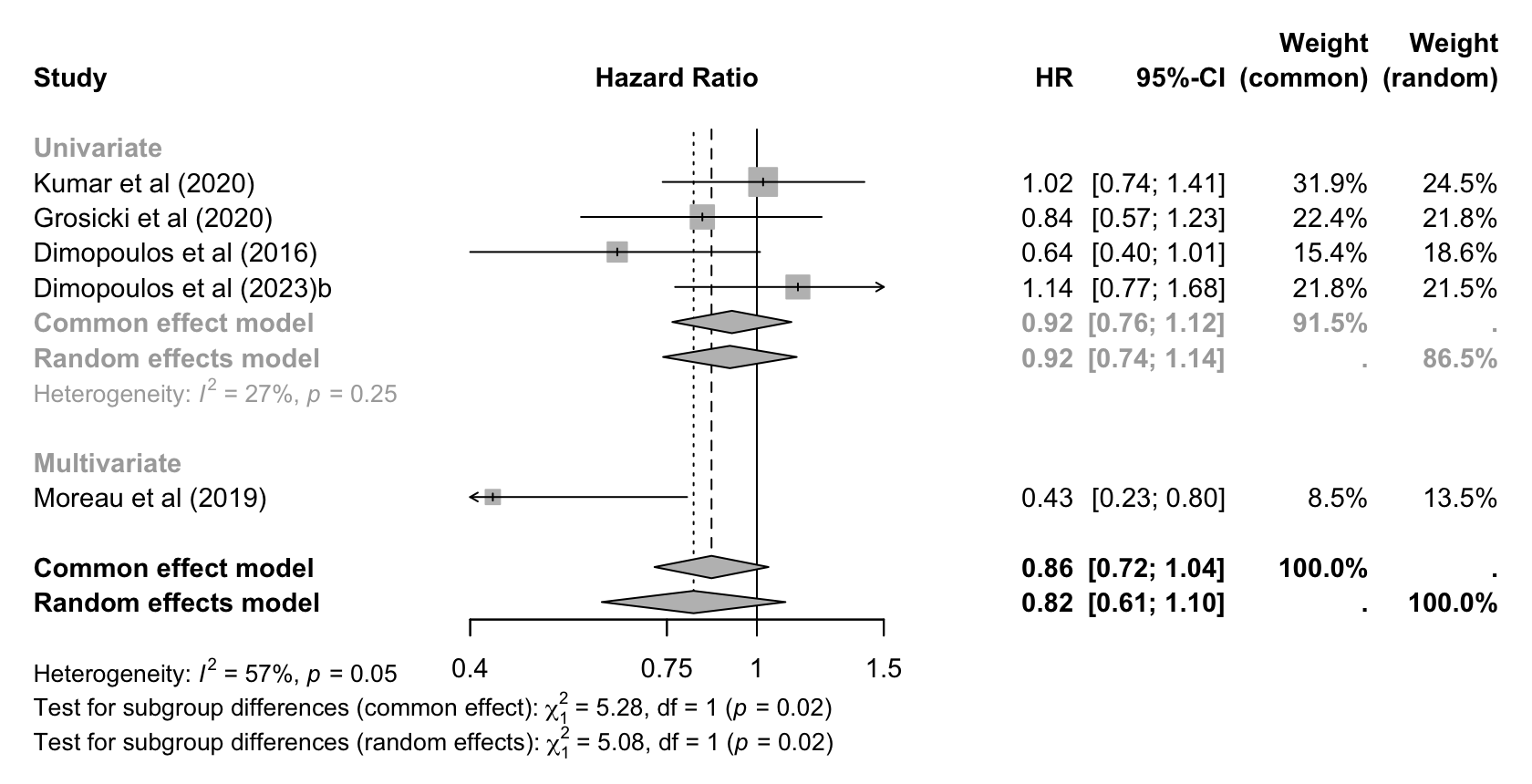


**Figure S33.**


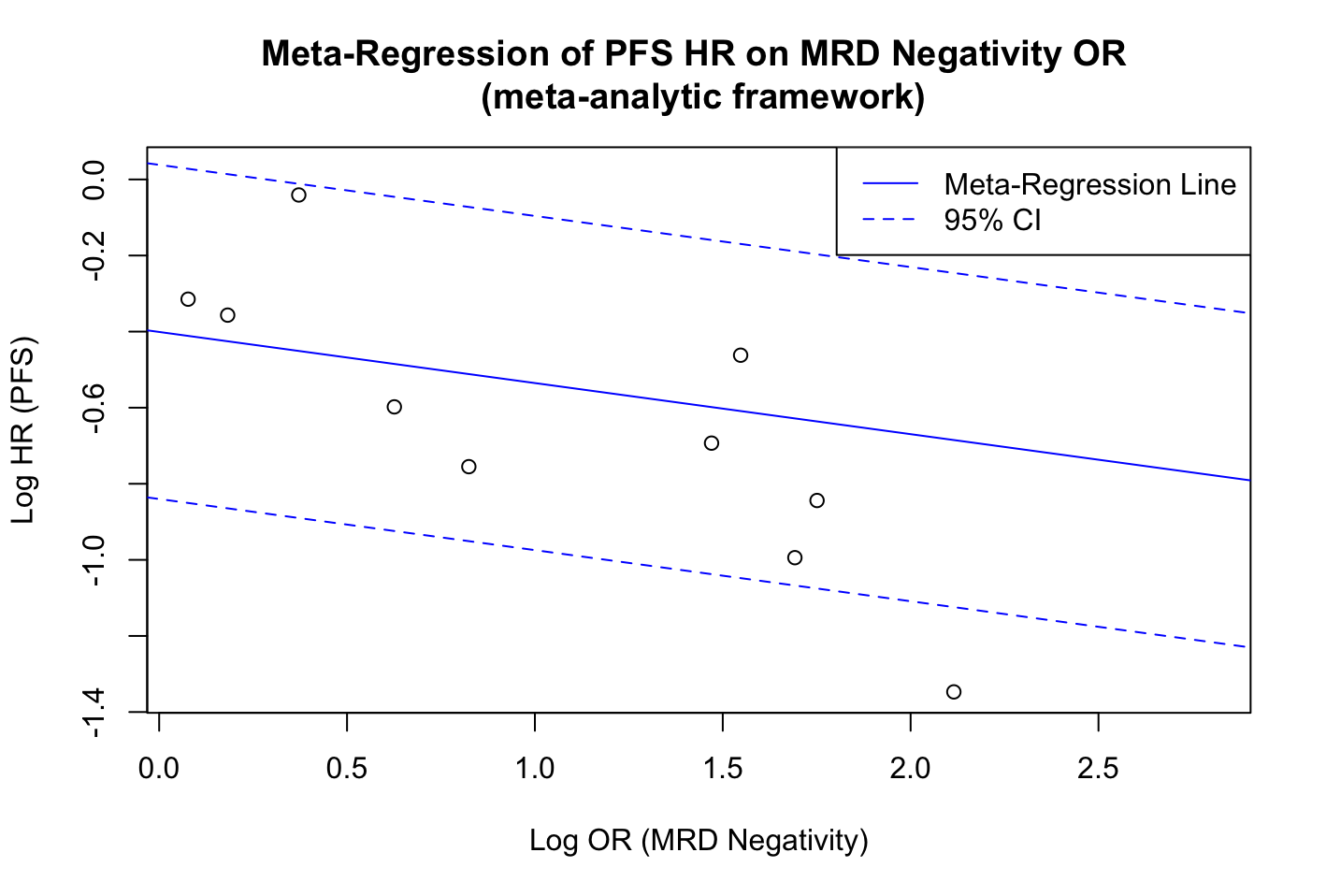


**Figure S34.** Funnel plot for MRD negativity ORs


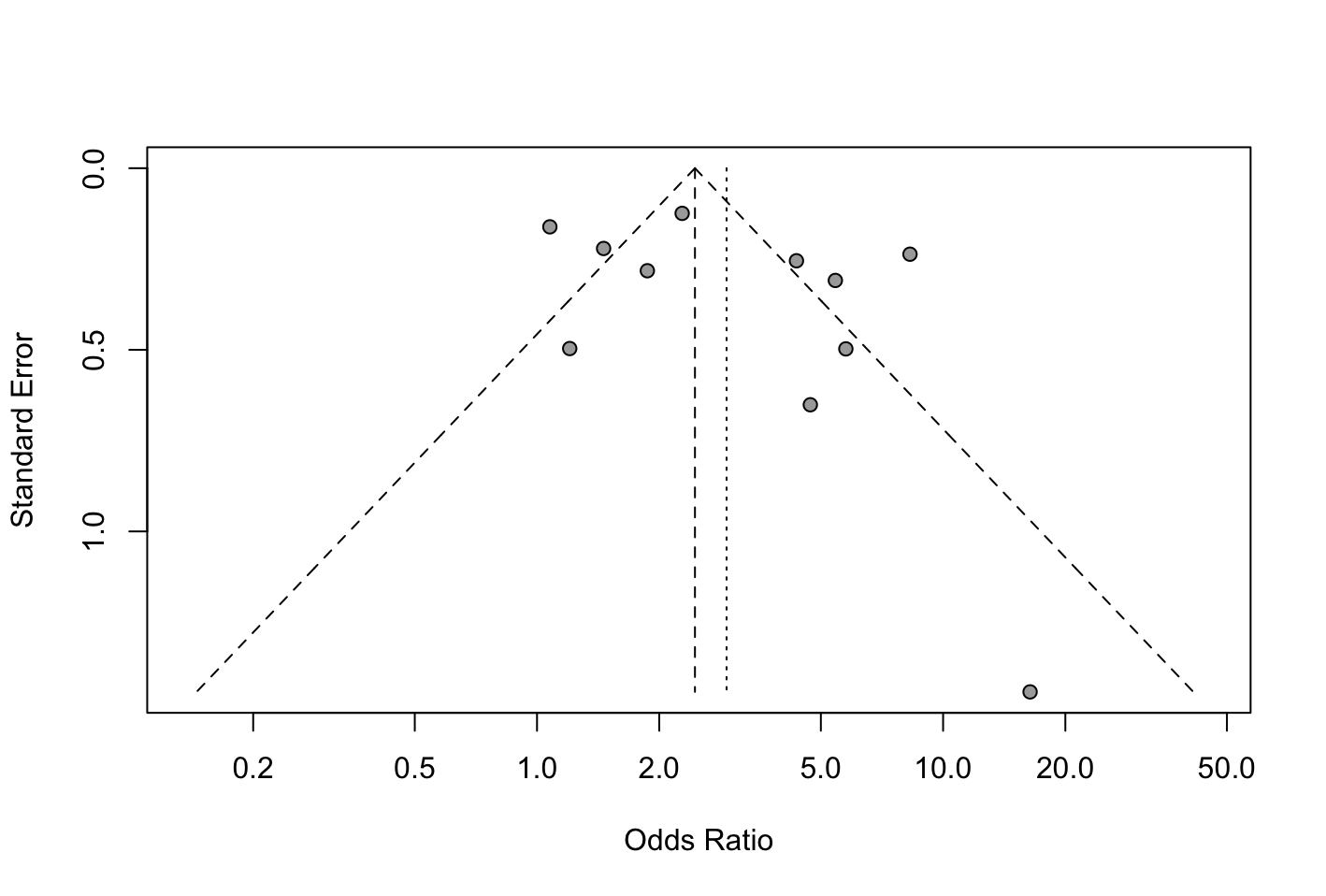


**Figure S35.** Funnel plot for PFS HRs


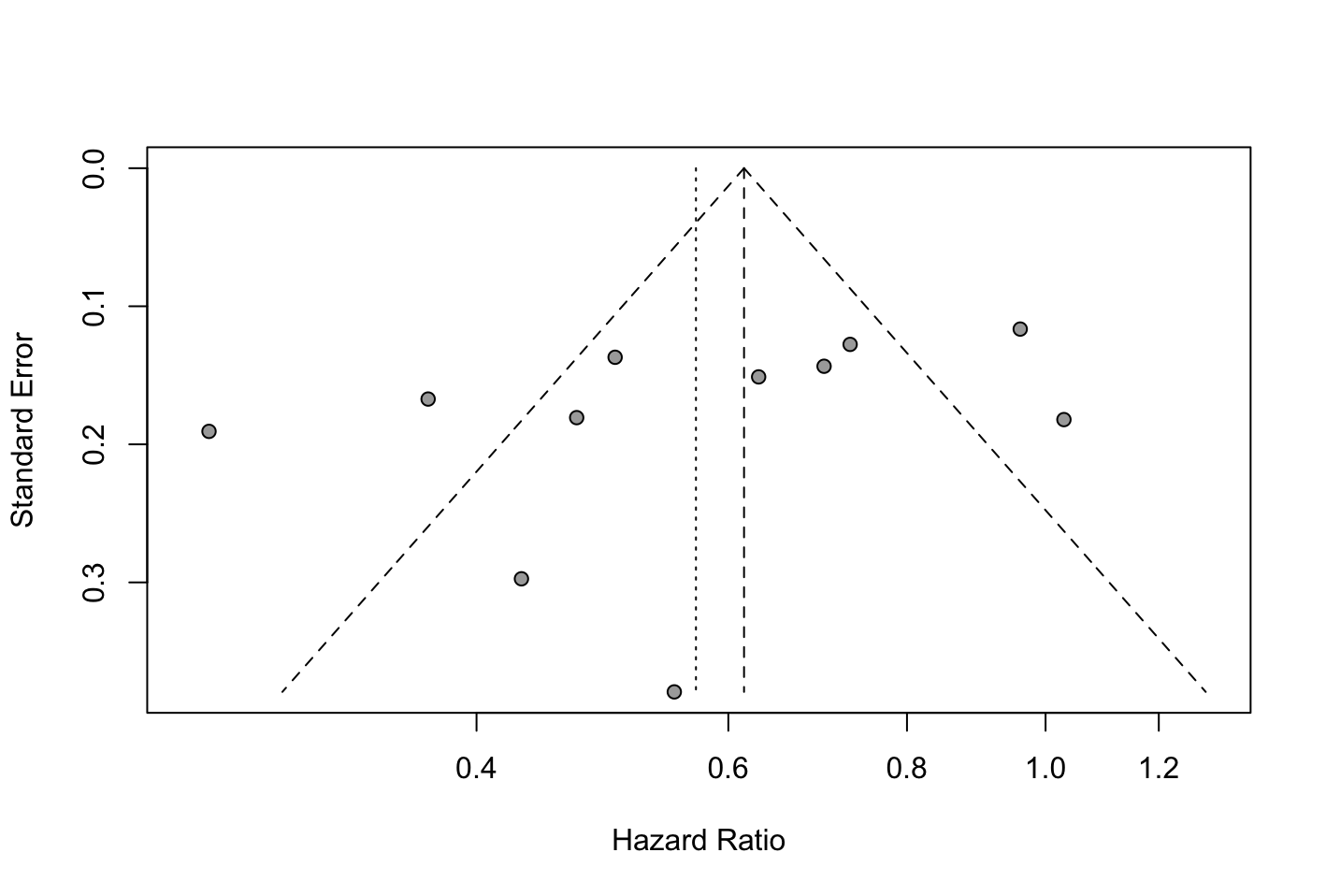


**Figure S36.** Funnel plot for OS HRs


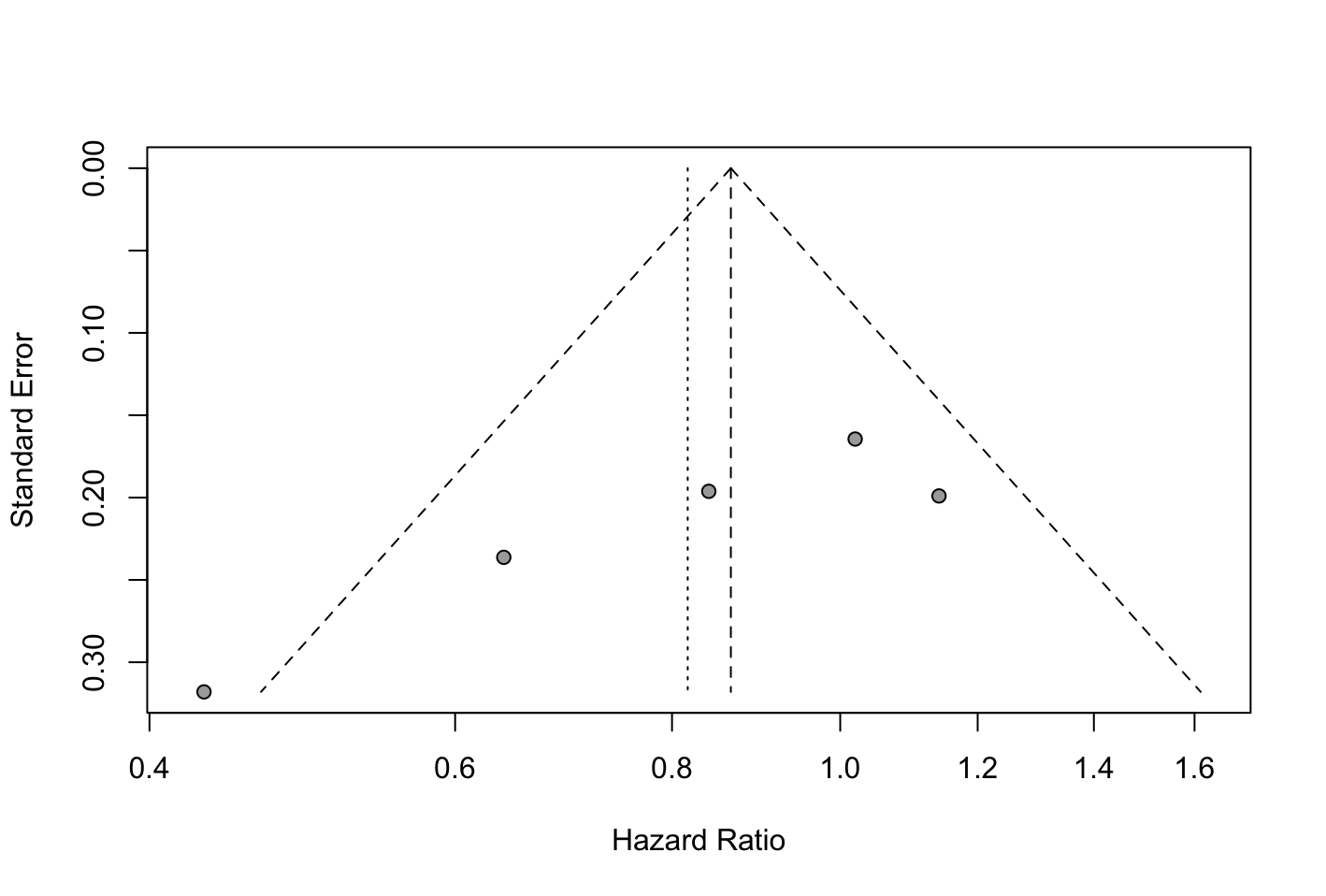


*2-year follow-up meta-analysis*

**Figure S37.** MRD negativity (OR) pooled-estimate for the base-case analysis (by MM setting)


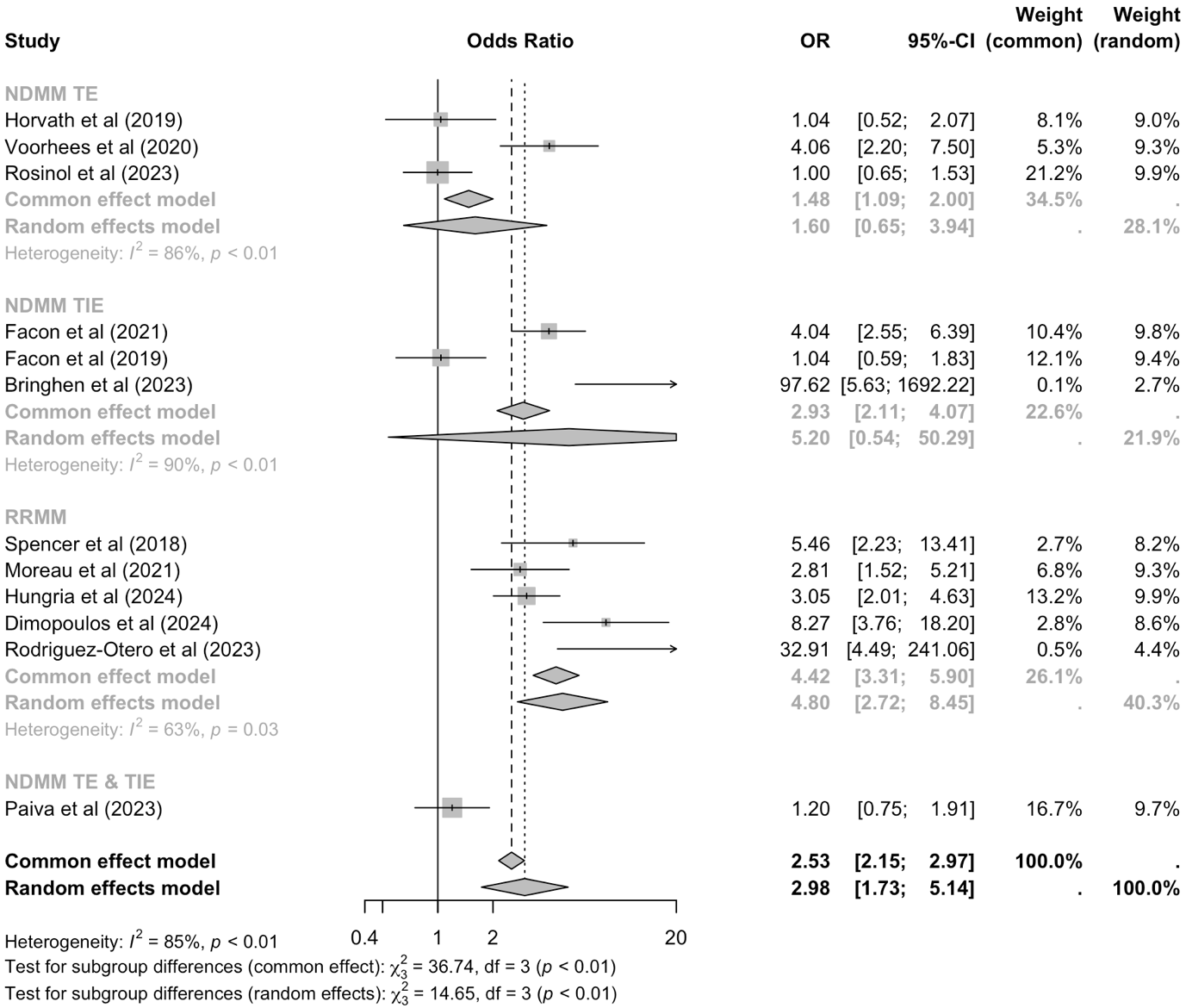


**Figure S38.** MRD negativity (OR) pooled-estimate for the base-case analysis (by region)


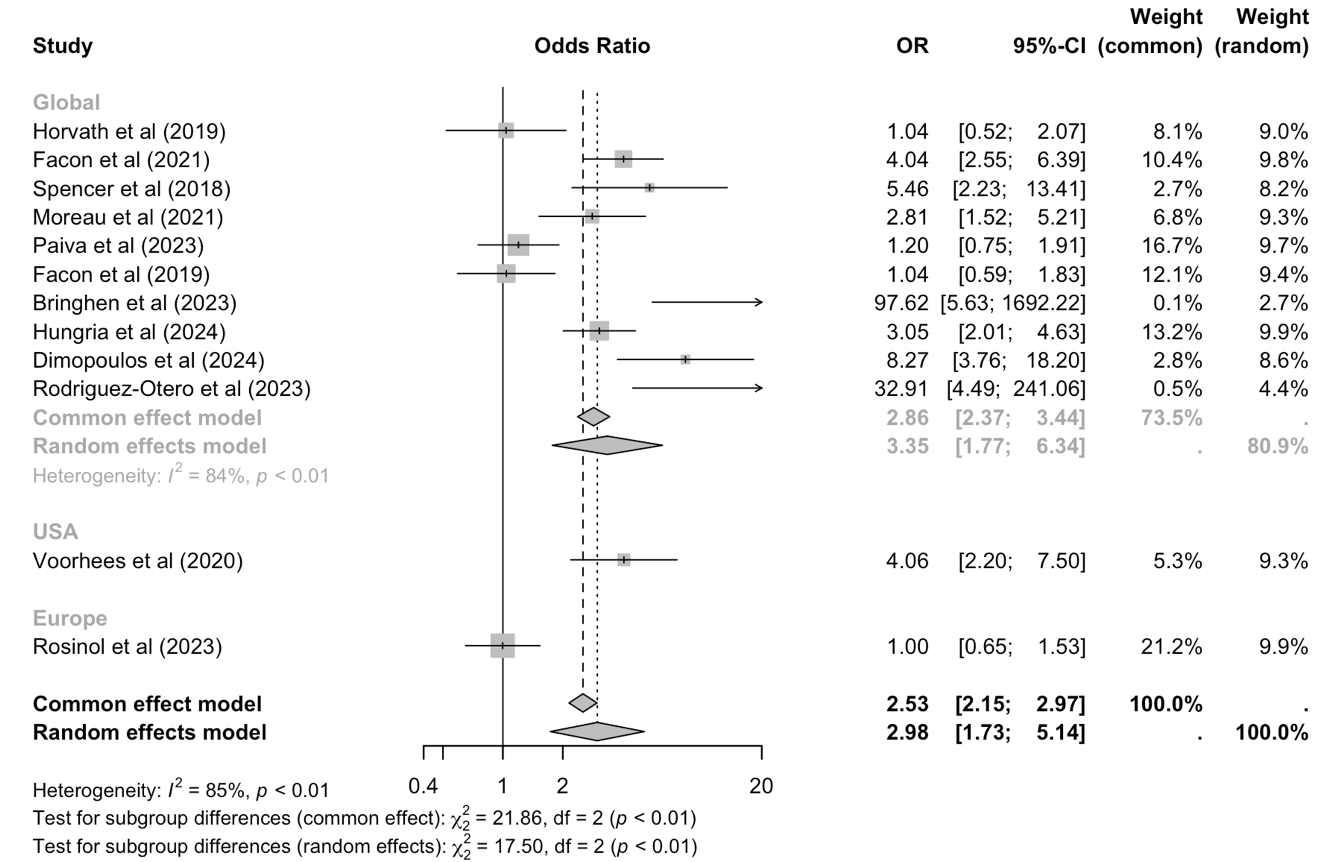


**Figure S39.** MRD negativity (OR) pooled-estimate for the base-case analysis (by treatment)


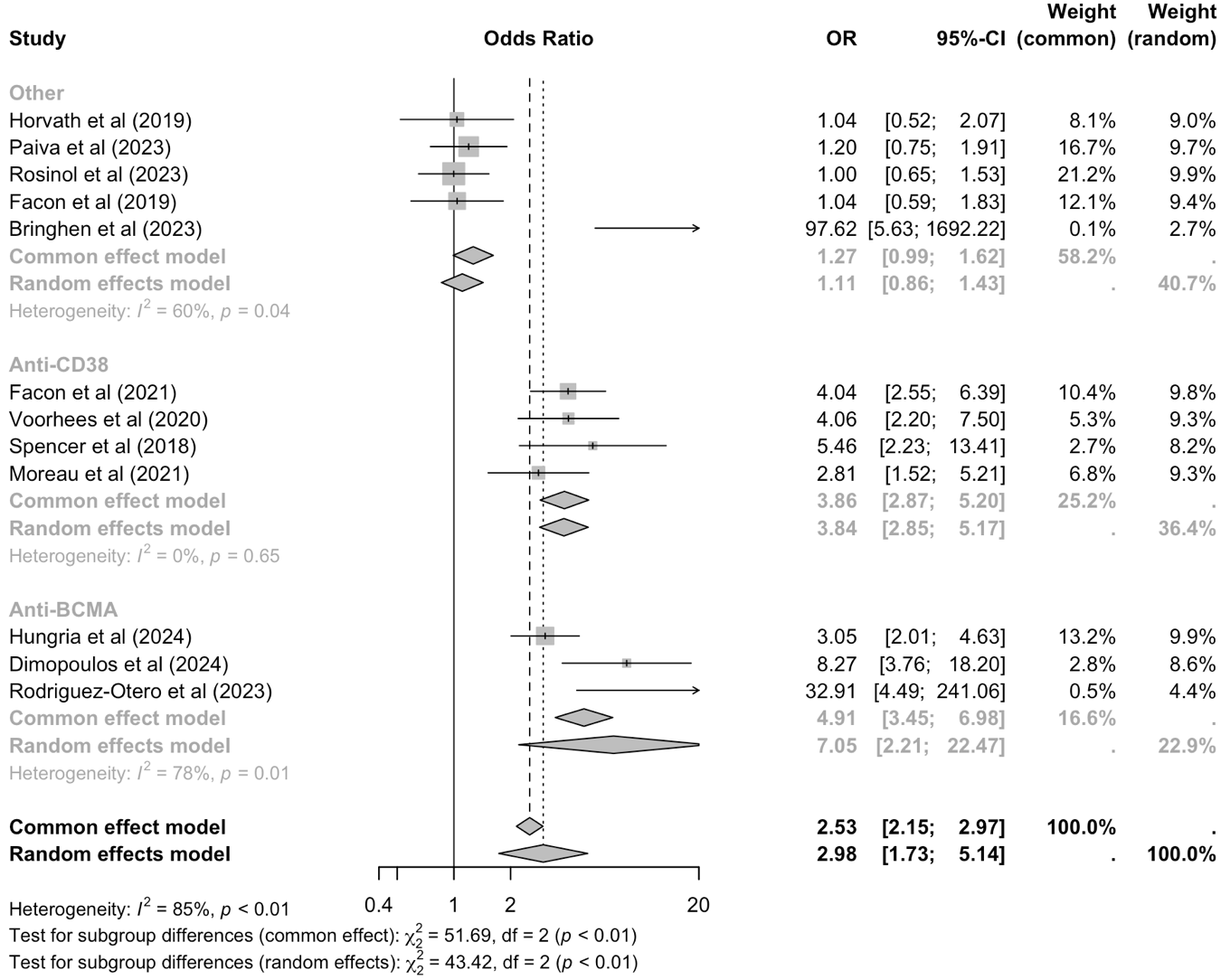


**Figure S40.** PFS (HR) pooled-estimate for the base-case analysis (by MM setting)


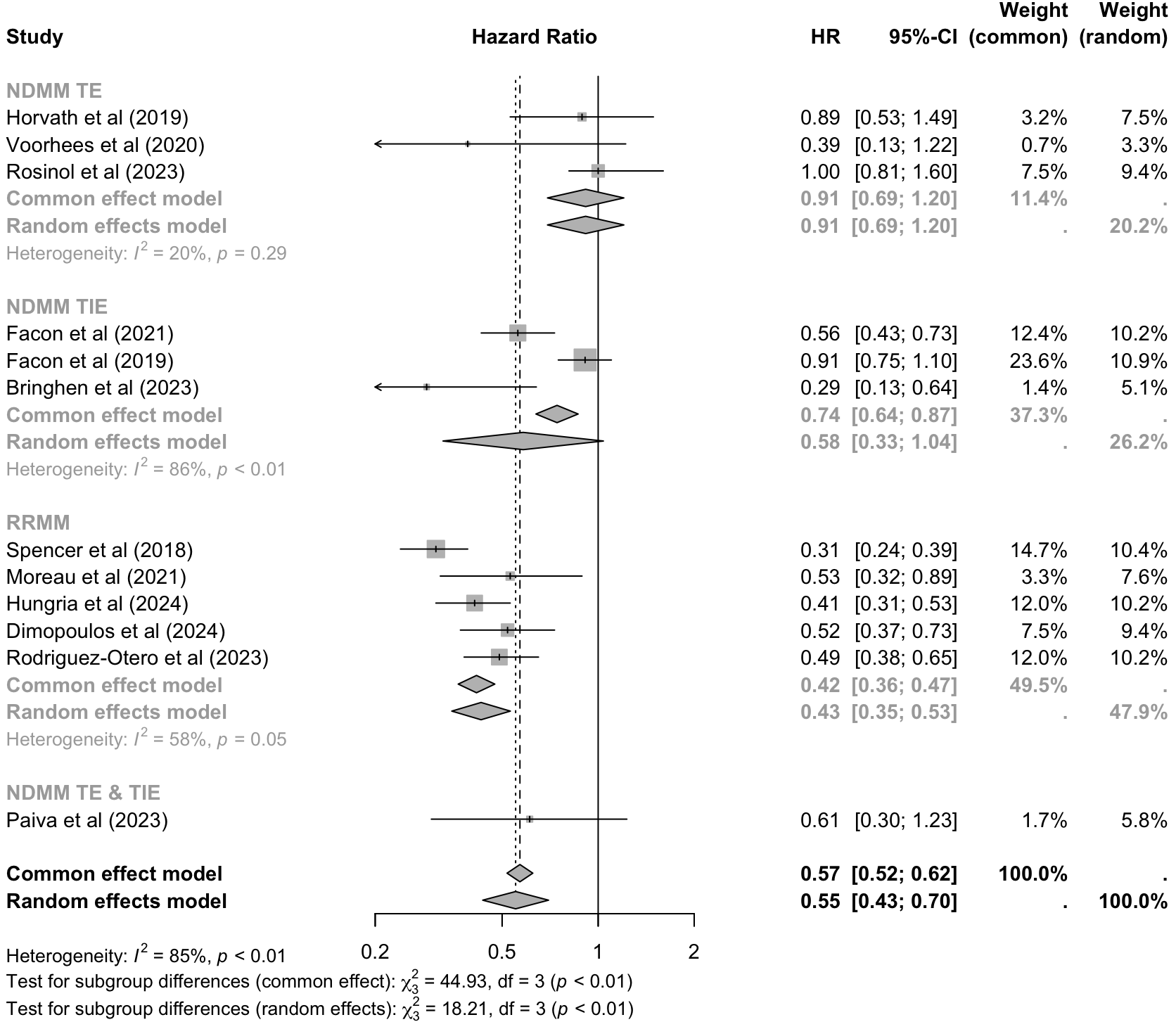


**Figure S41.** PFS (HR) pooled-estimate for the base-case analysis (by region)


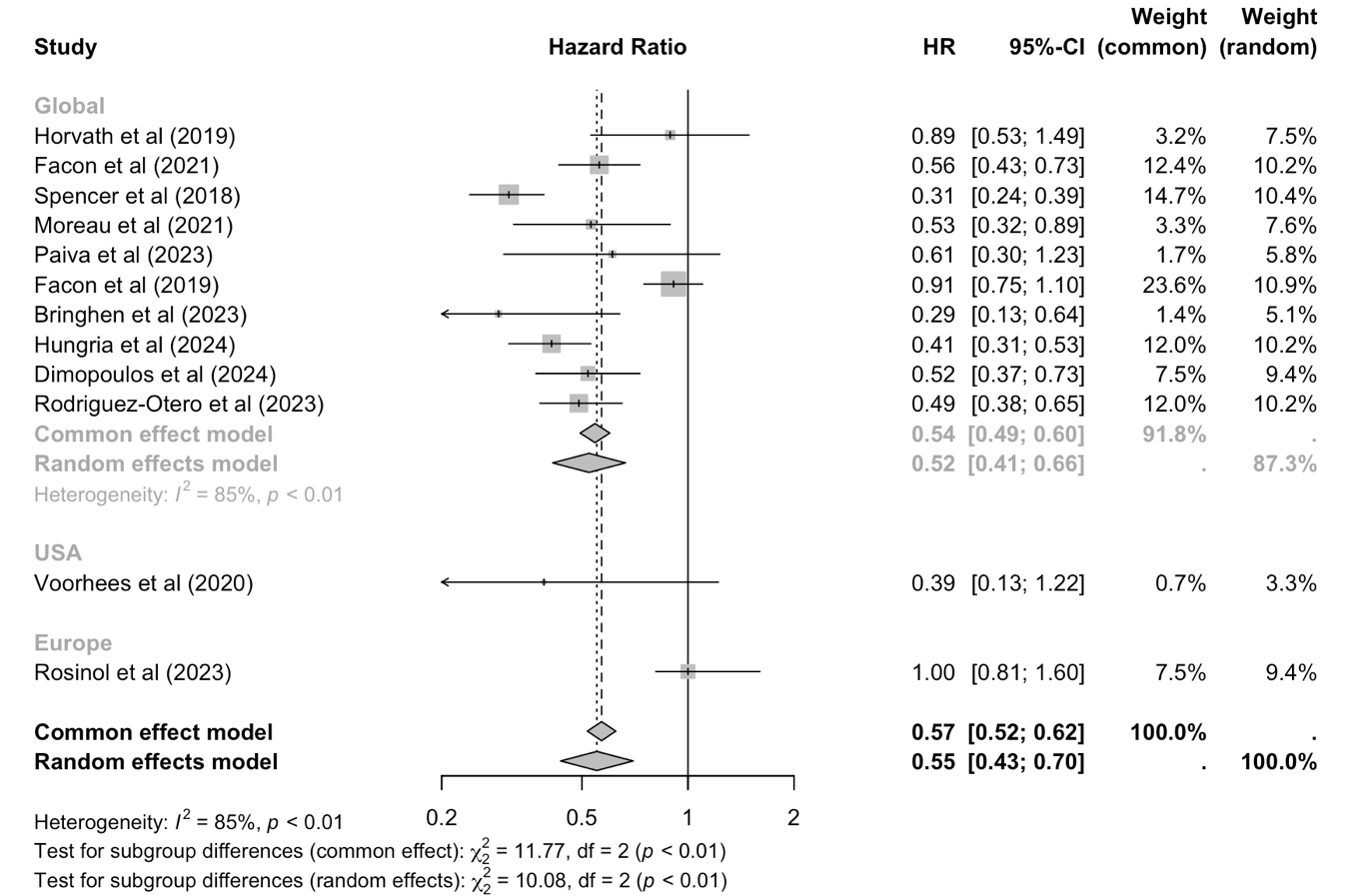


**Figure S42.** PFS (HR) pooled-estimate for the base-case analysis (by treatment)


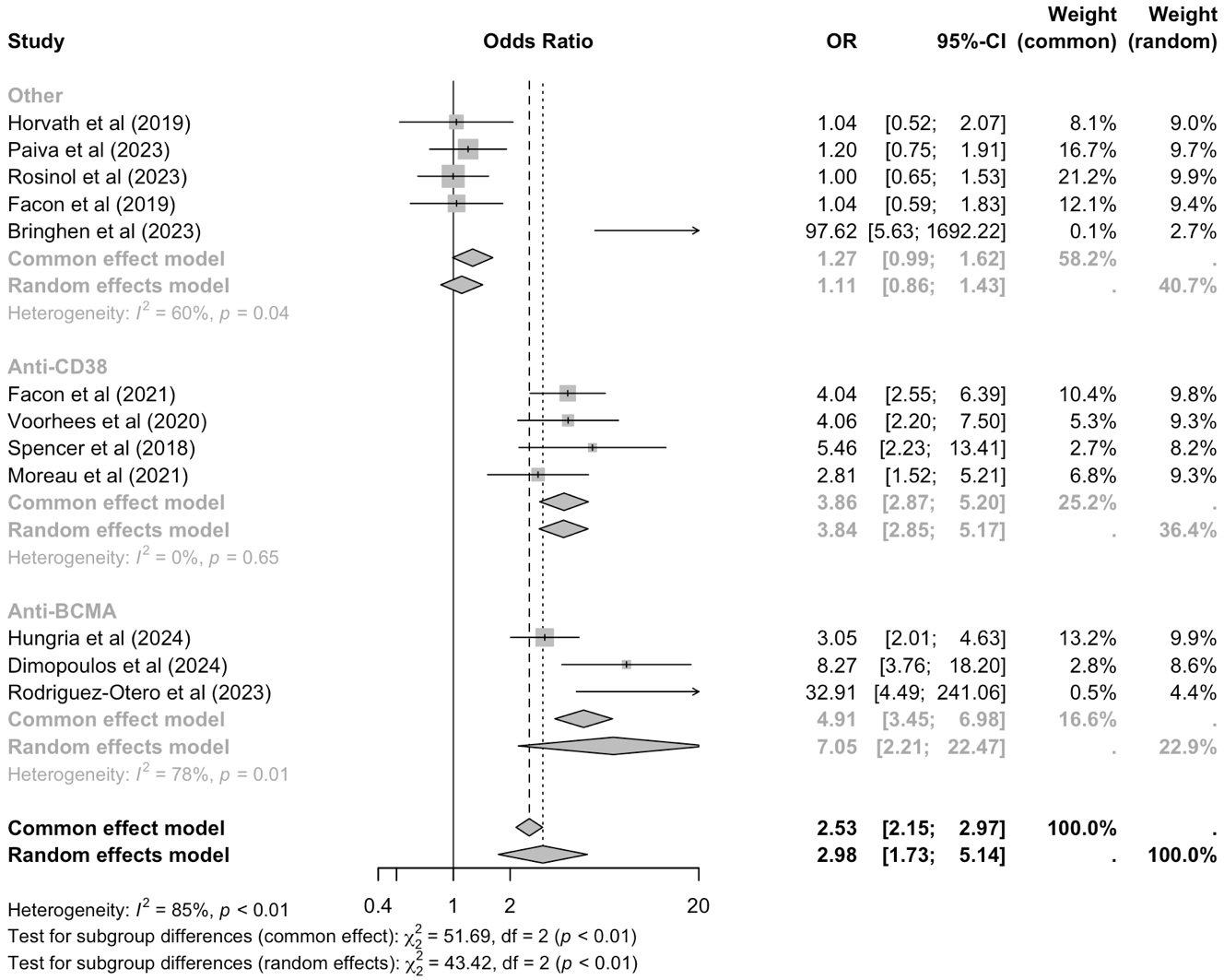


**Figure S43.** PFS (HR) pooled-estimate for the base-case analysis (by adjustment)


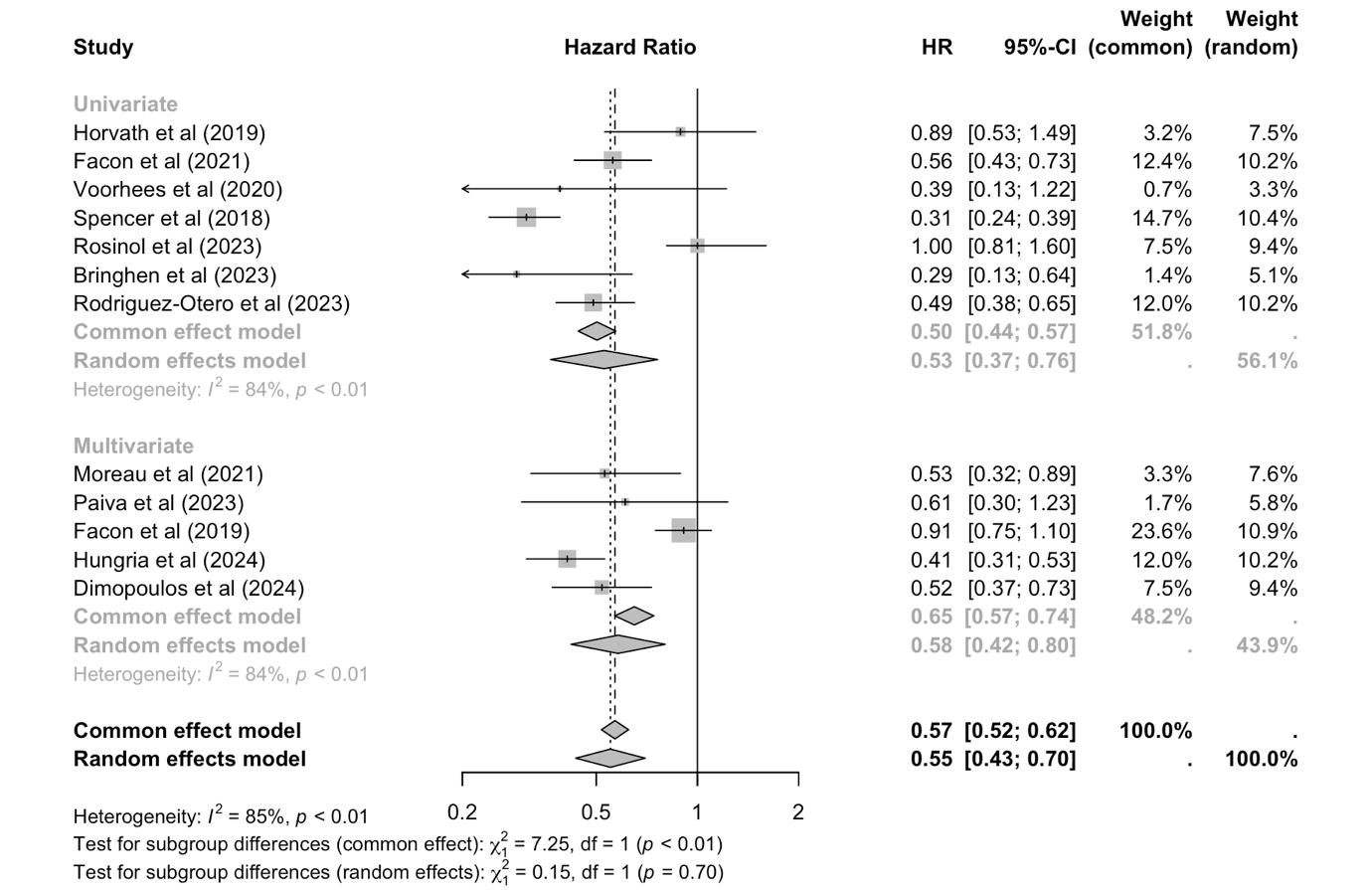


**Figure S44.**

**
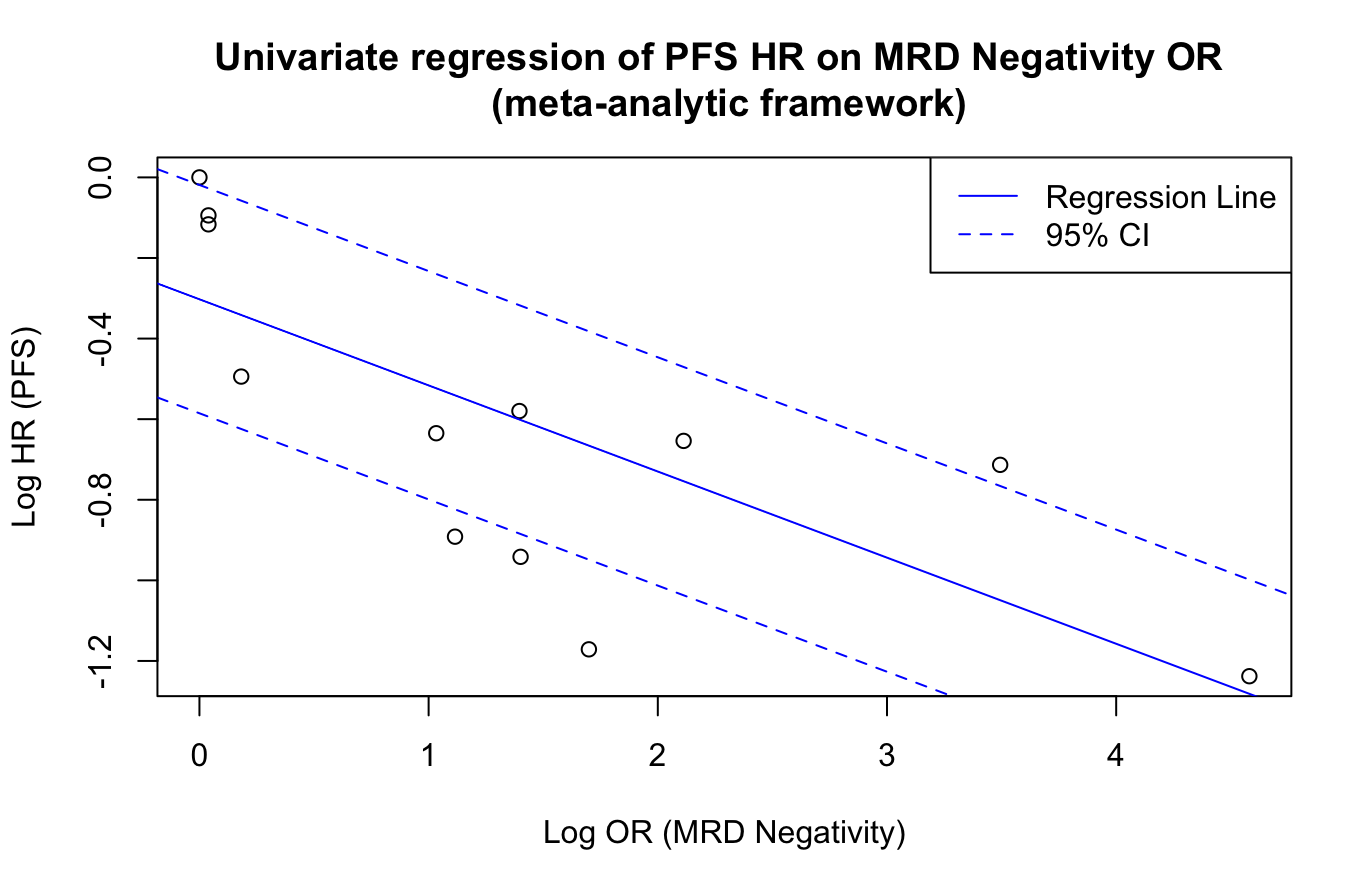
**

**Figure S45.** Funnel plot for MRD negativity ORs


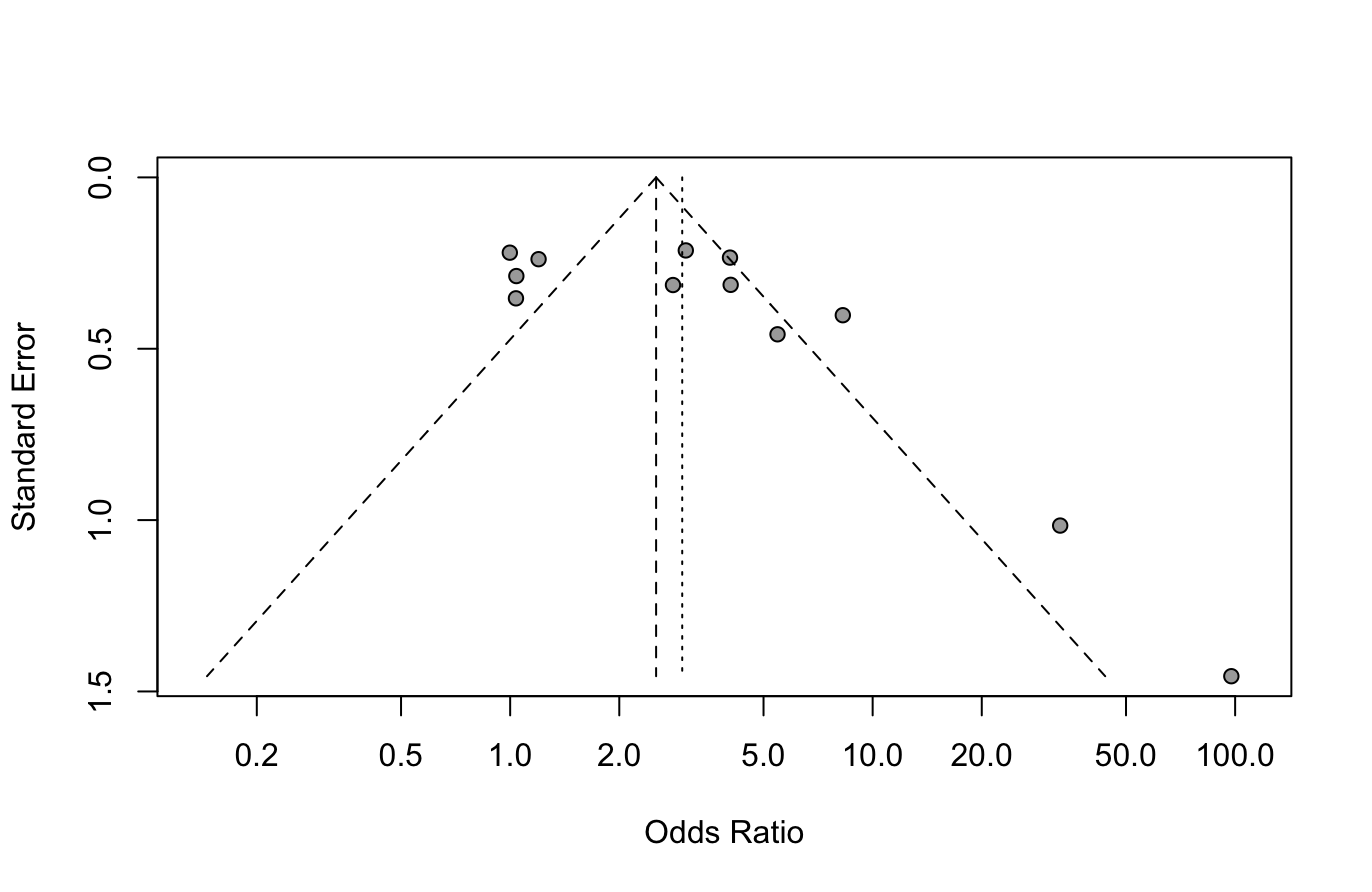


**Figure S46.** Funnel plot for PFS HRs


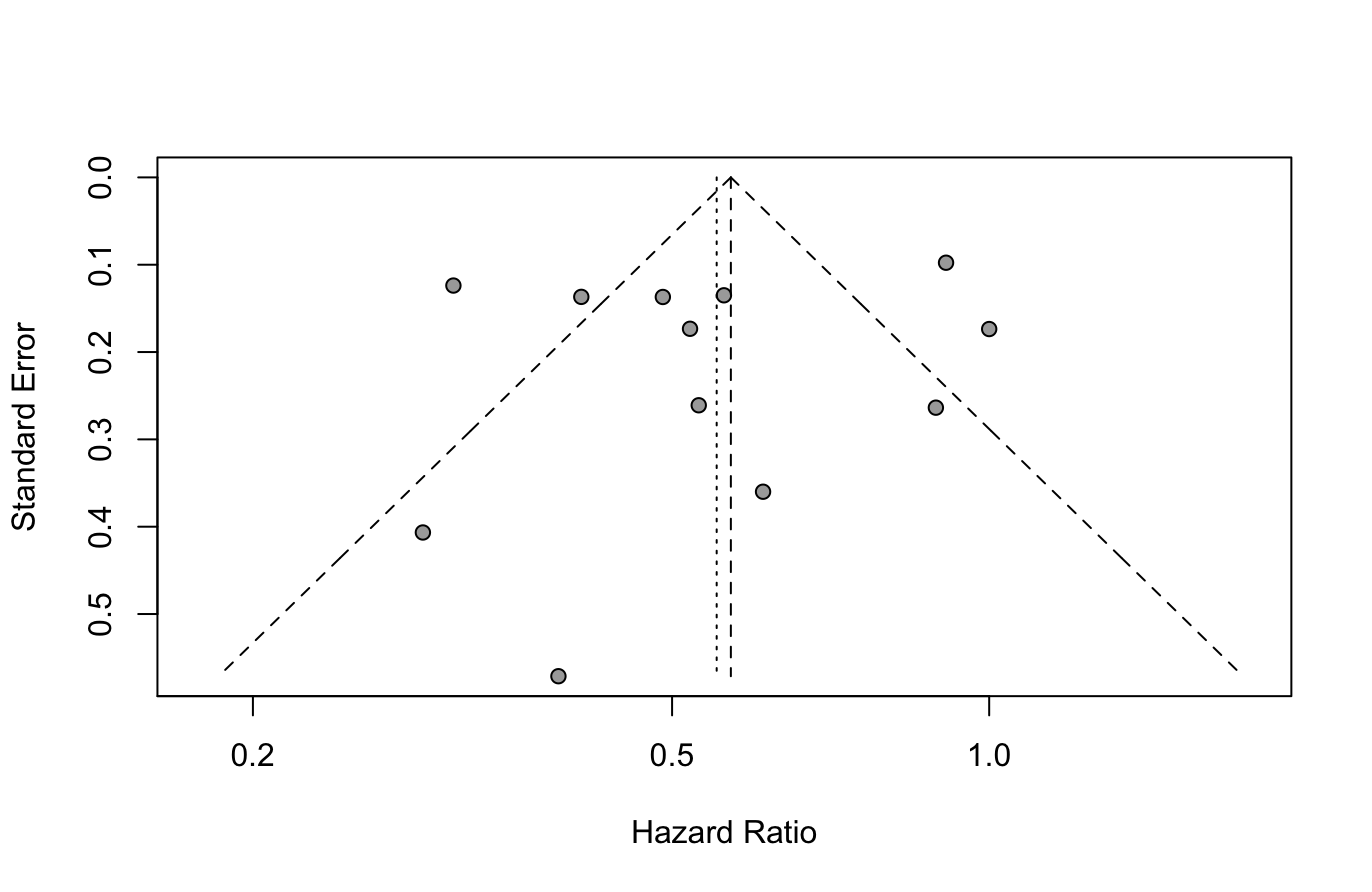


*3-year follow-up meta-analysis*

**Figure S47.** MRD negativity (OR) pooled-estimate for the base-case analysis (by setting)


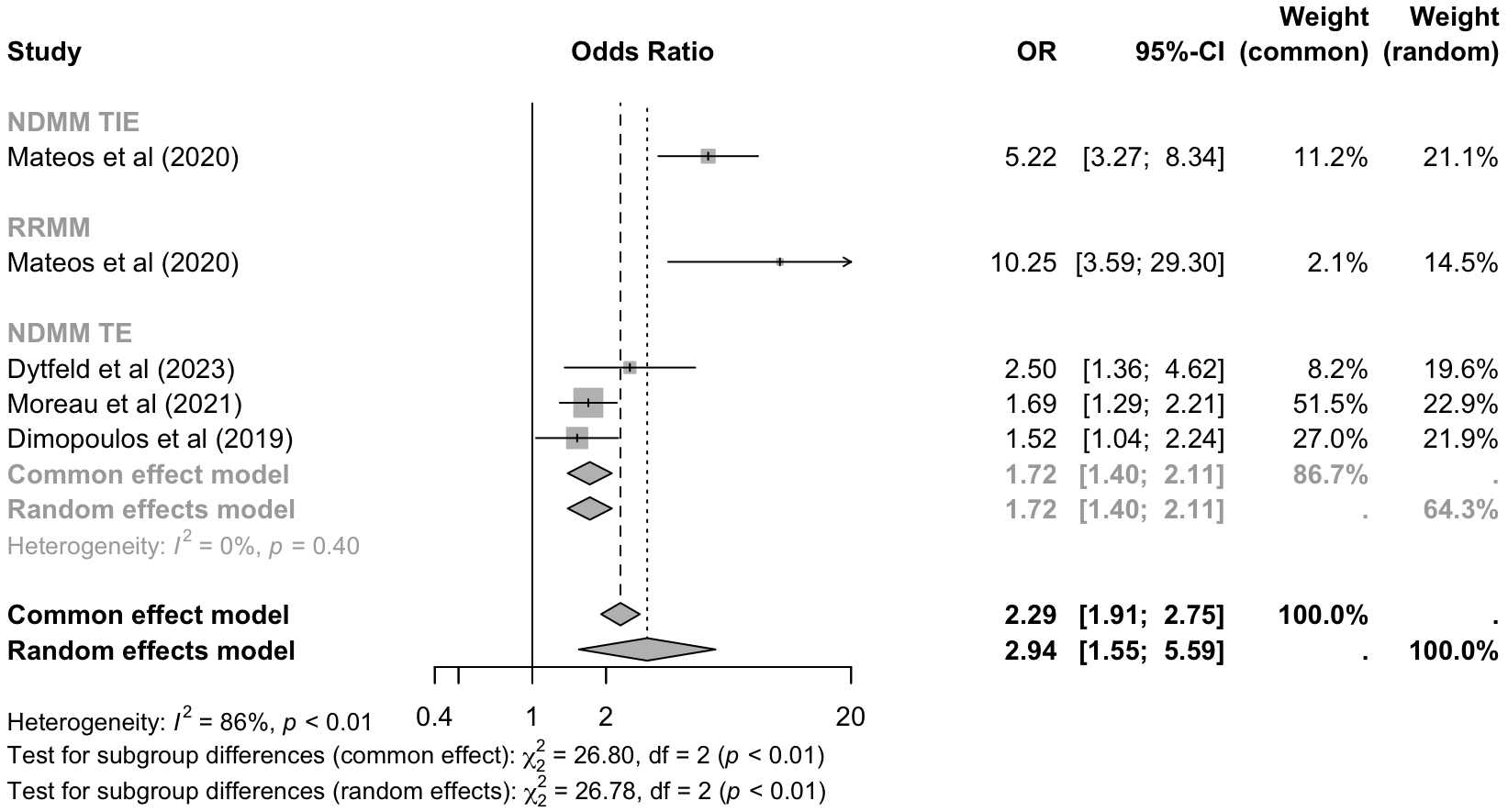


**Figure S48.** MRD negativity (OR) pooled-estimate for the base-case analysis (by region)


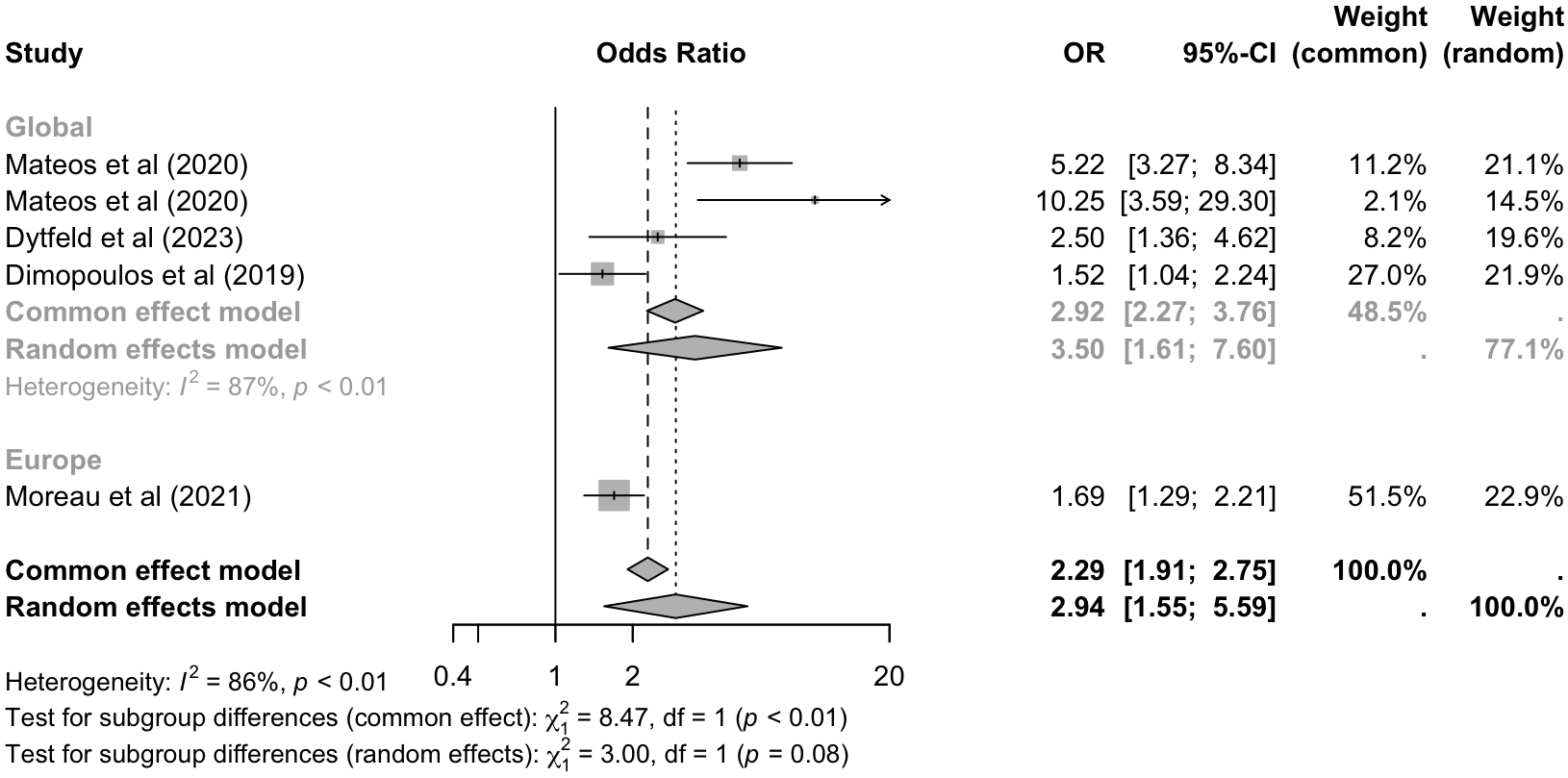


**Figure S49.** MRD negativity (OR) pooled-estimate for the base-case analysis (by treatment)

**
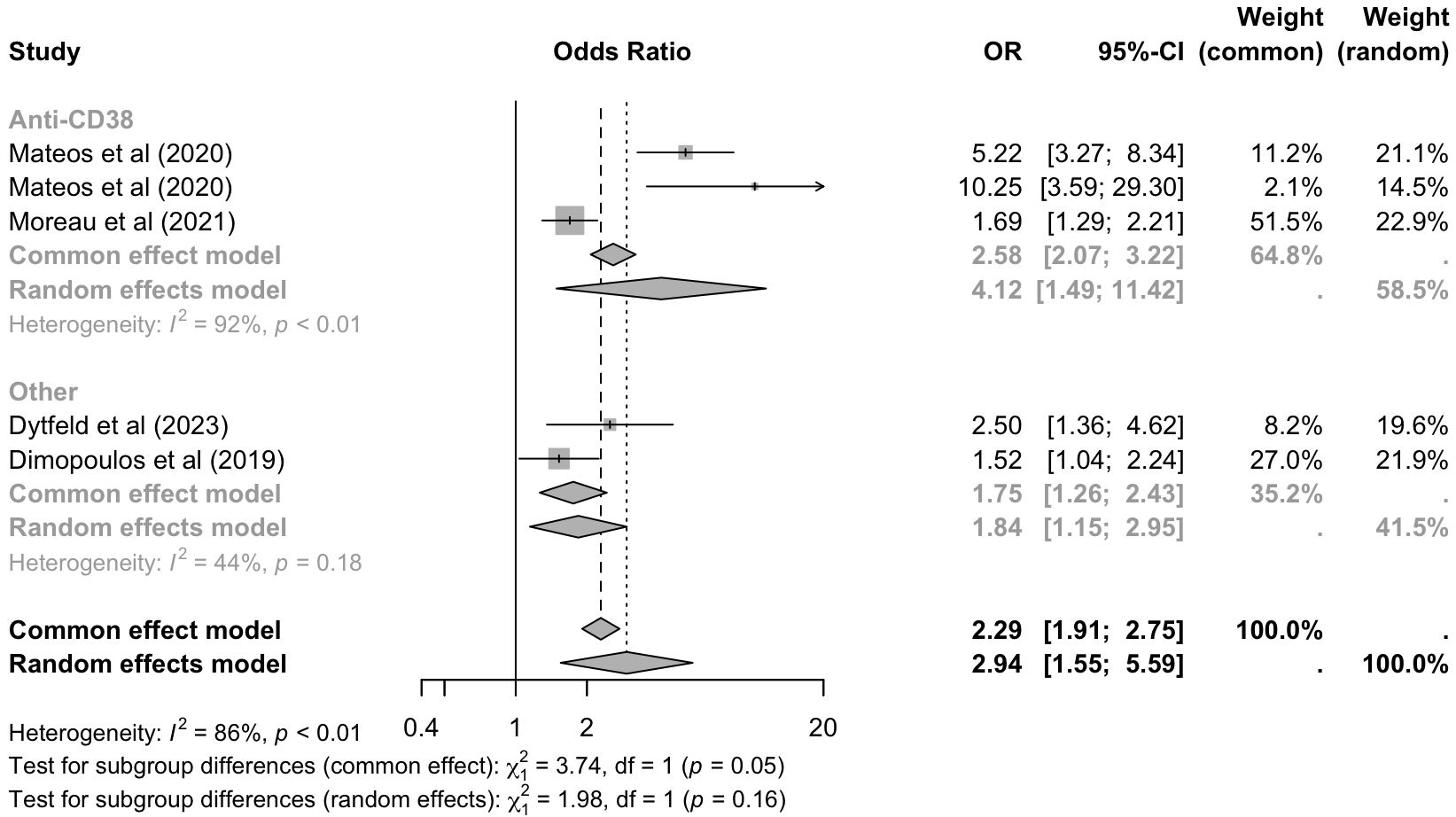
**

**Figure S50.** PFS (HR) pooled-estimate for the base-case analysis (by setting)


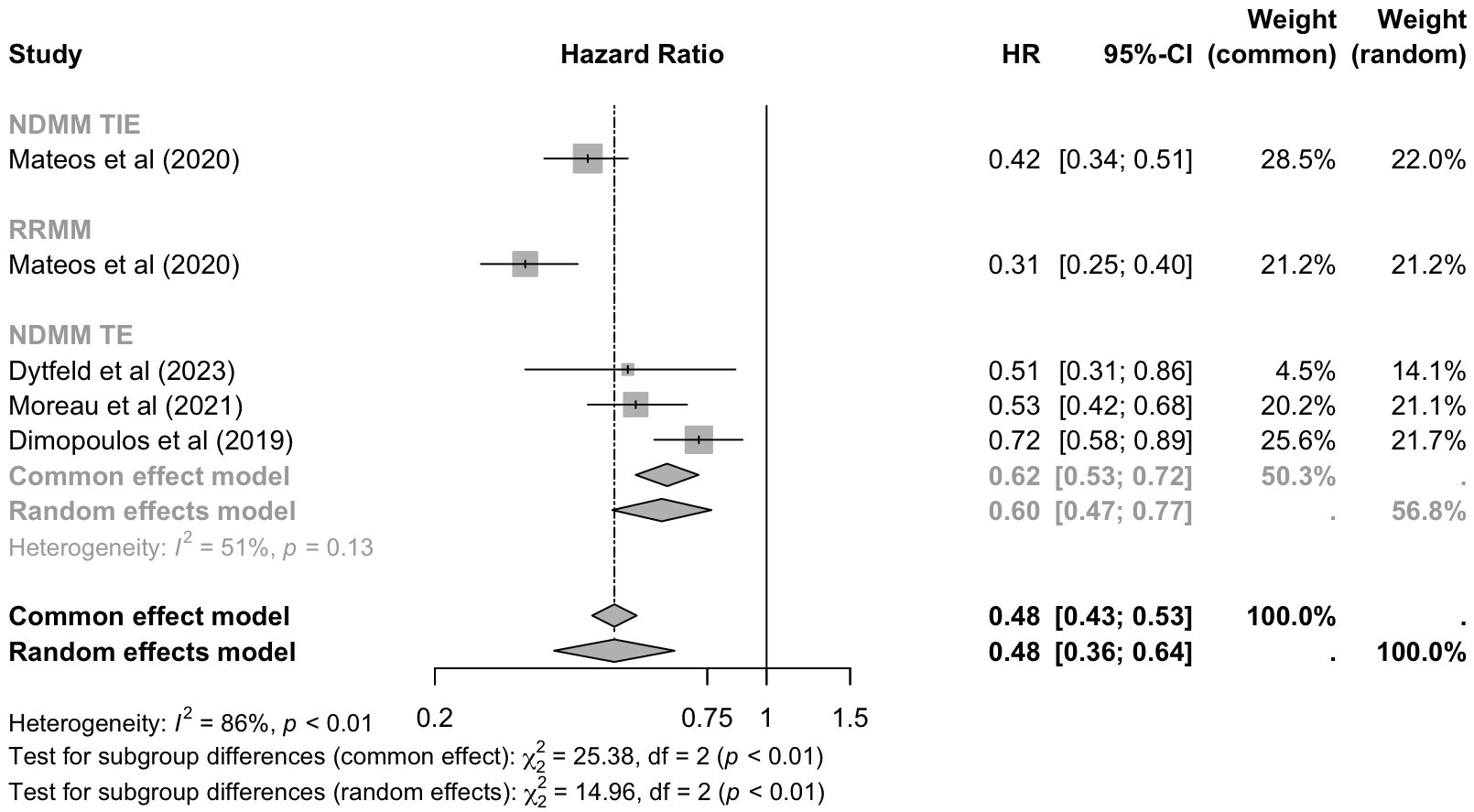


**Figure S51.** PFS (HR) pooled-estimate for the base-case analysis (by region)


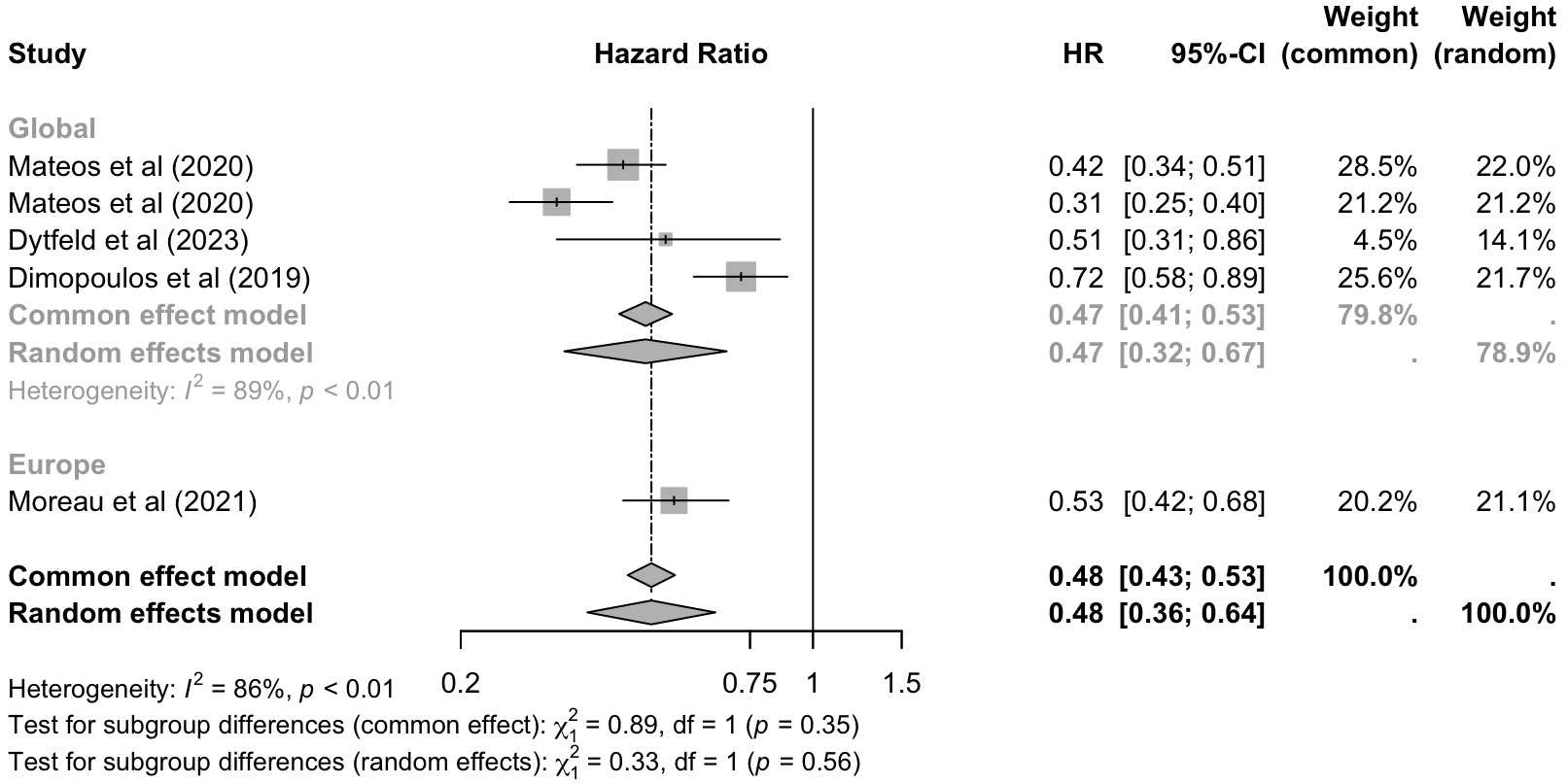


**Figure S52.** PFS (HR) pooled-estimate for the base-case analysis (by treatment)


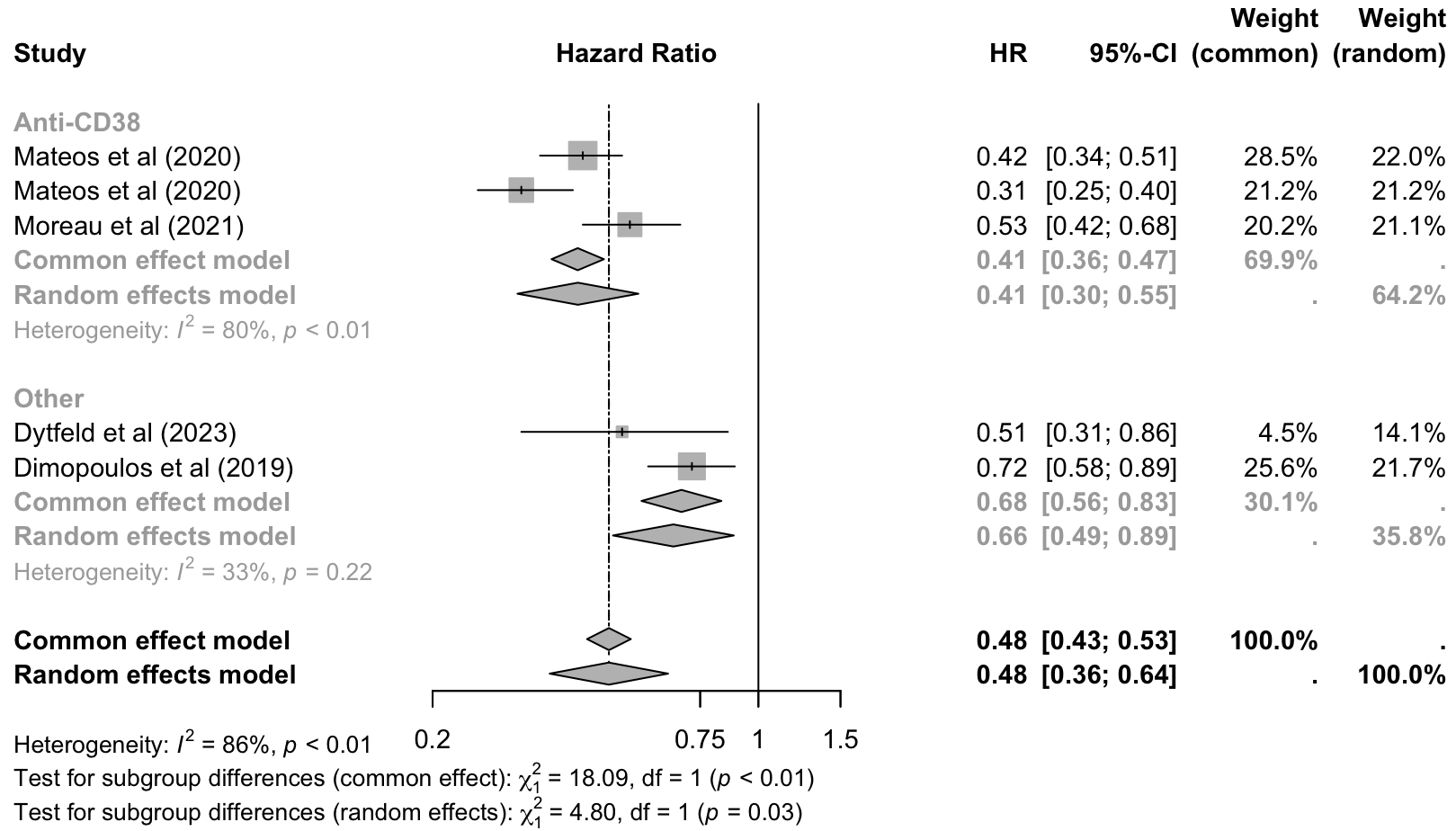


**Figure S53.** PFS (HR) pooled-estimate for the base-case analysis (by adjustment)


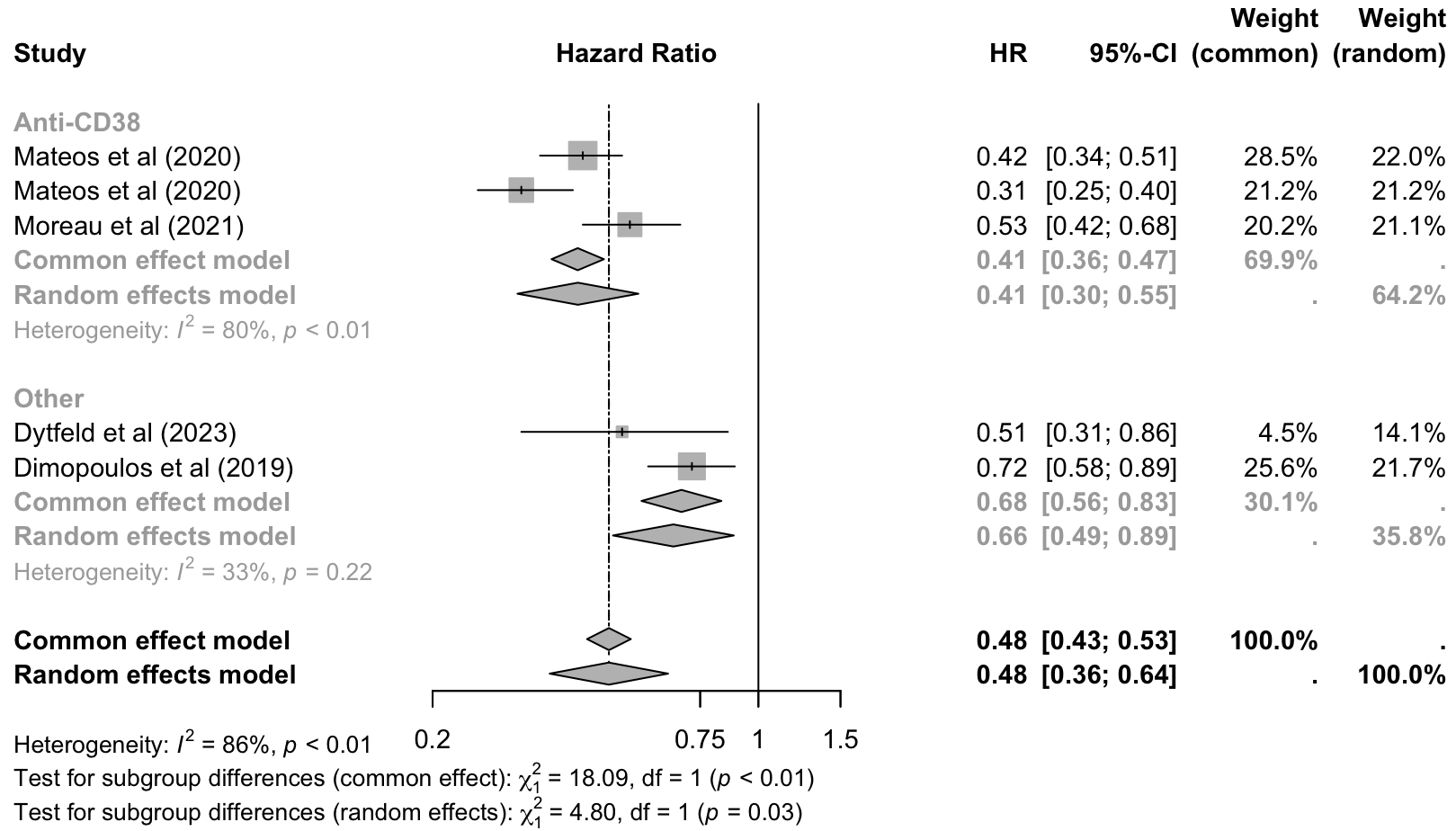


**Figure S54.** Funnel plot for MRD negativity ORs


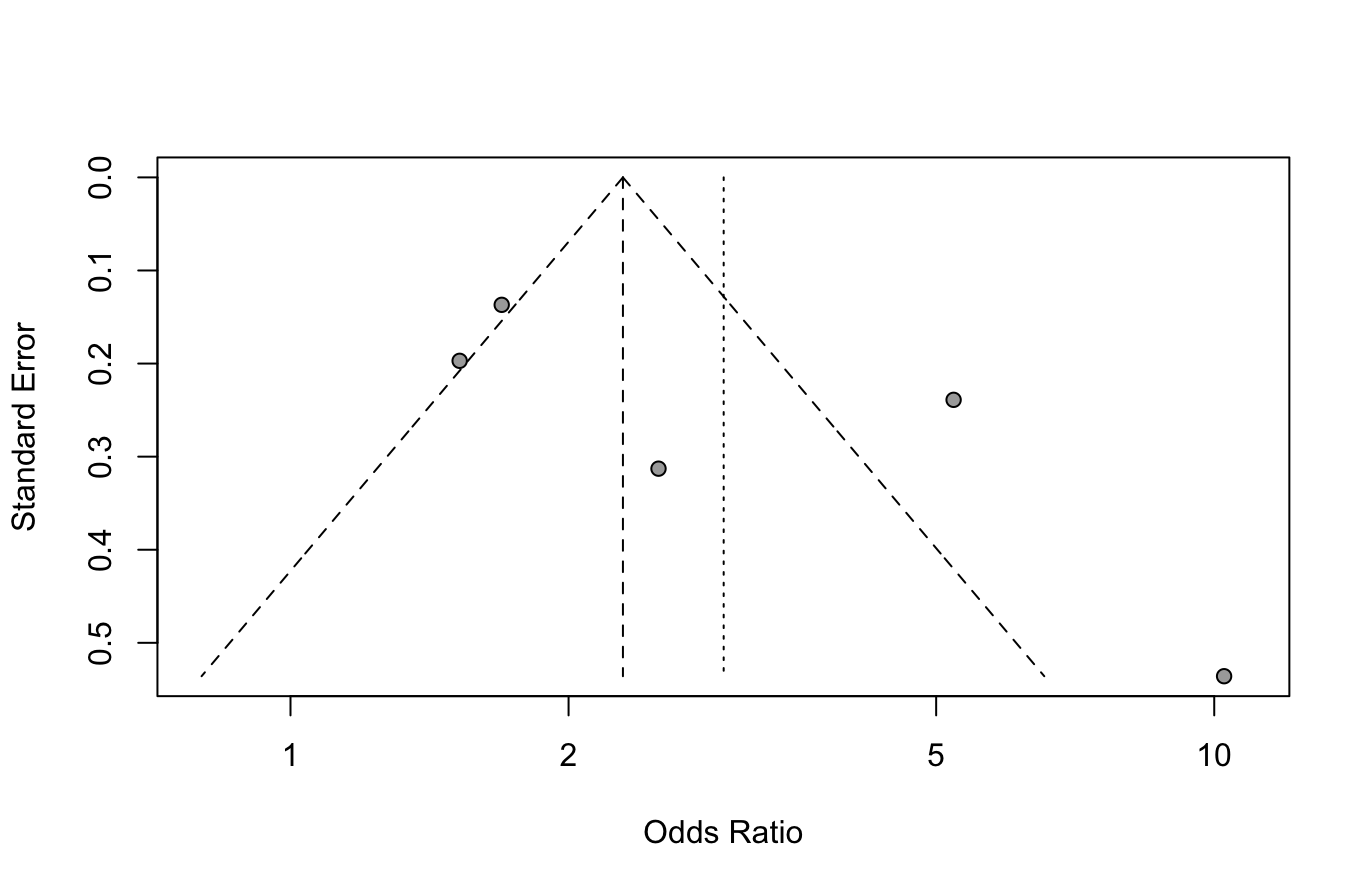


**Figure S55.** Funnel plot for PFS HRs


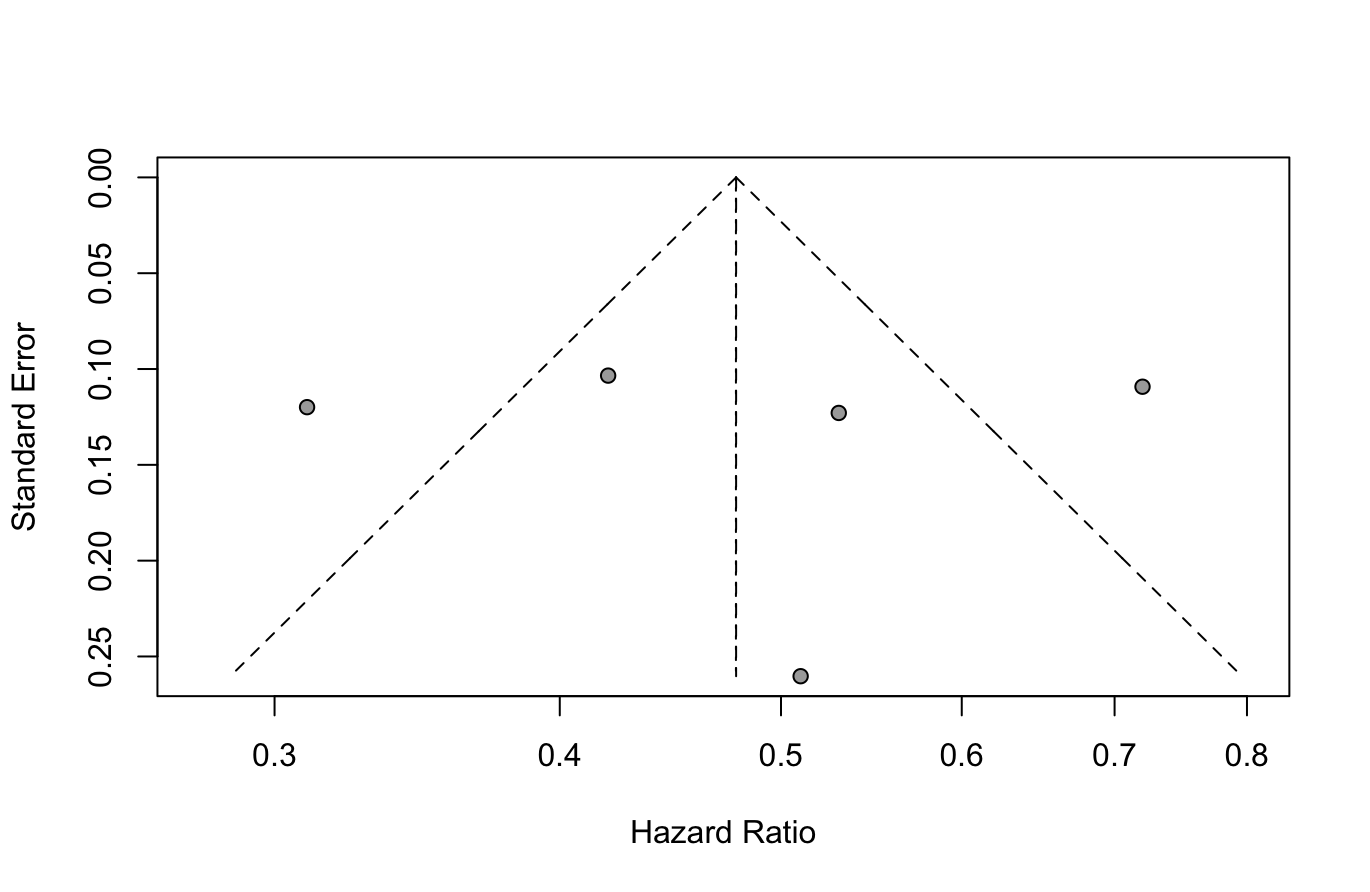


*4-year follow-up meta-analysis*

**Figure S56.** MRD negativity (OR) pooled-estimate for the base-case analysis (by setting)


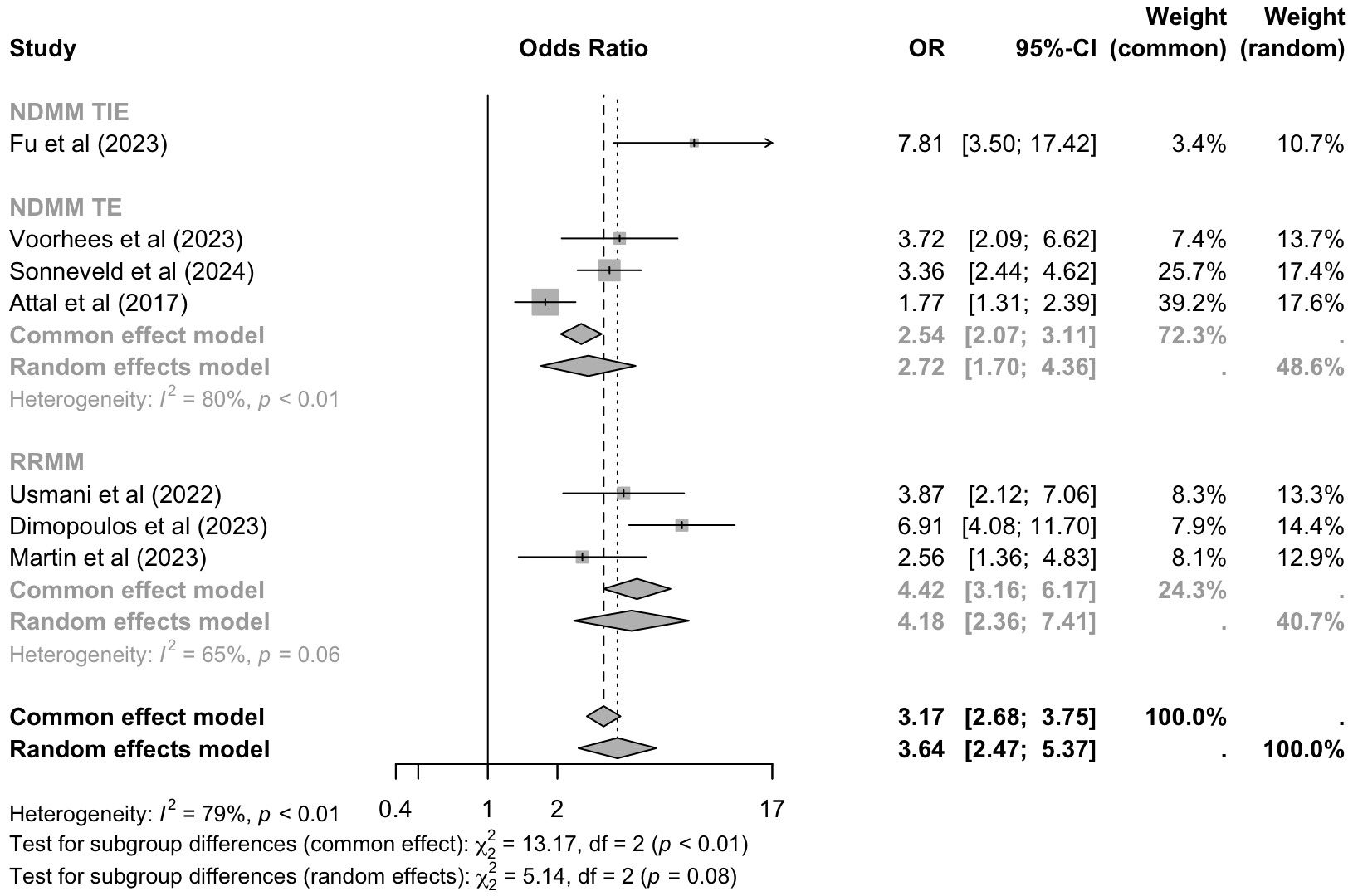


**Figure S57.** MRD negativity (OR) pooled-estimate for the base-case analysis (by region)


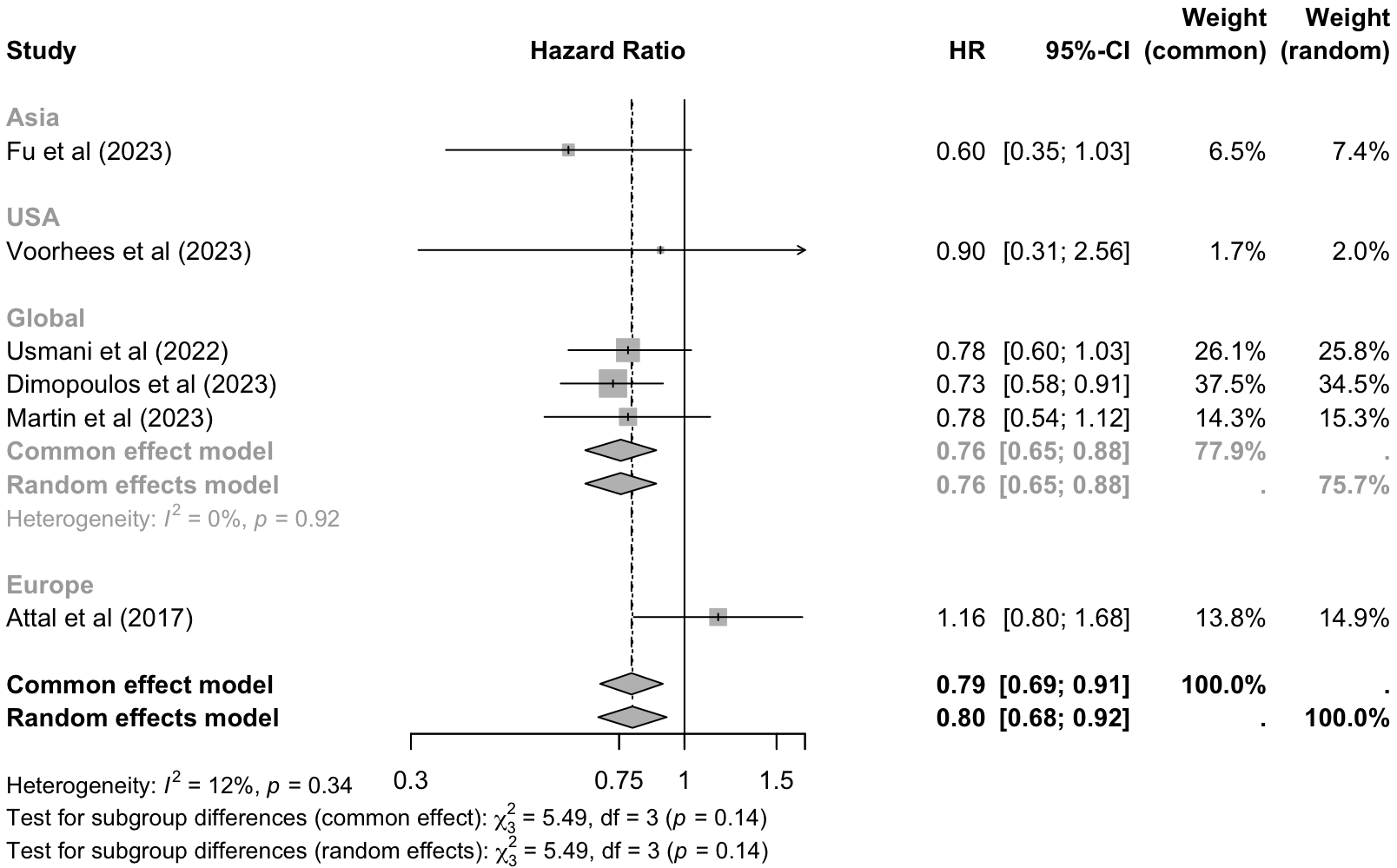


**Figure S58.** MRD negativity (OR) pooled-estimate for the base-case analysis (by treatment)


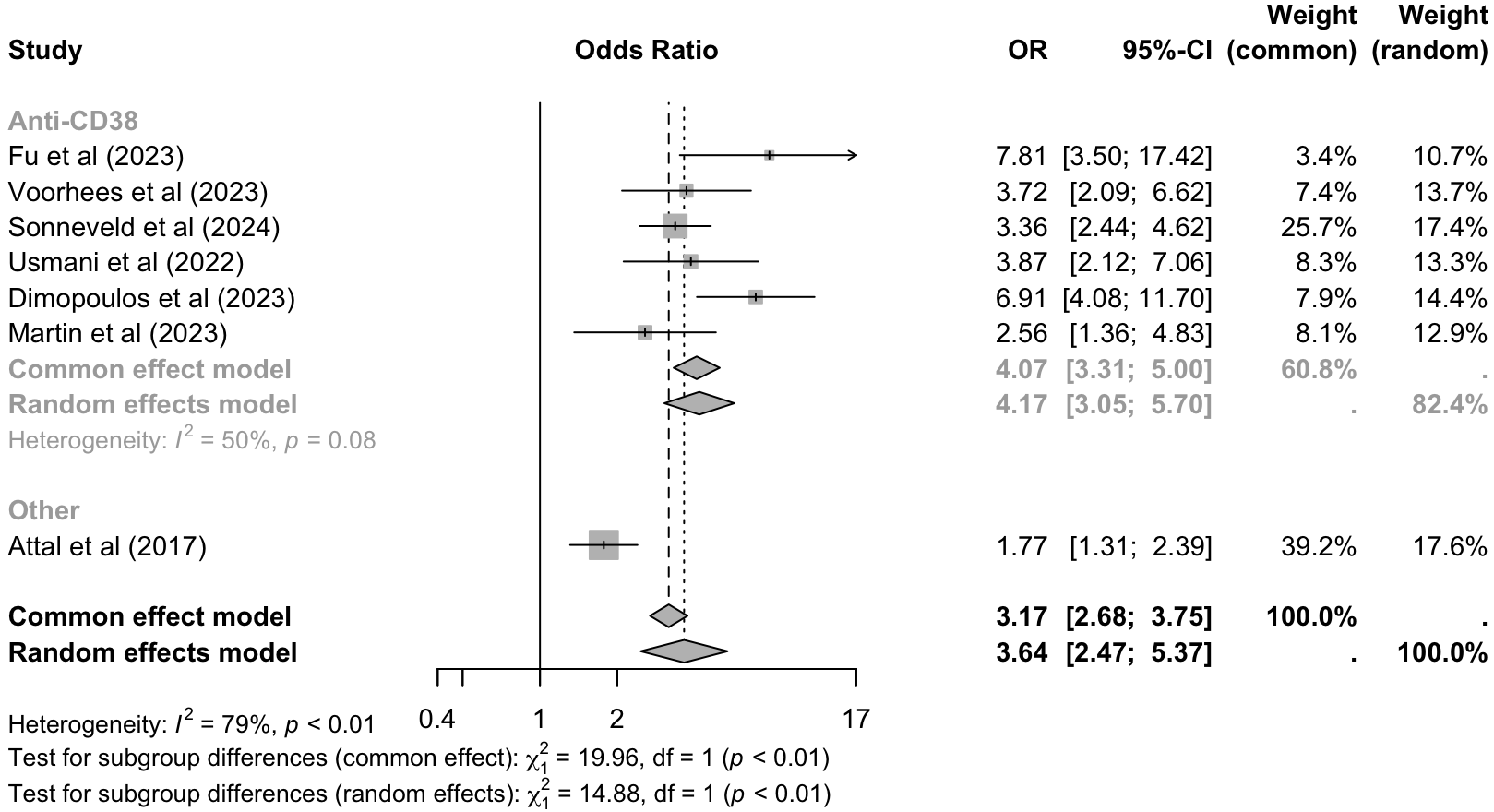


**Figure S59.** PFS (HR) pooled-estimate for the base-case analysis (by setting)


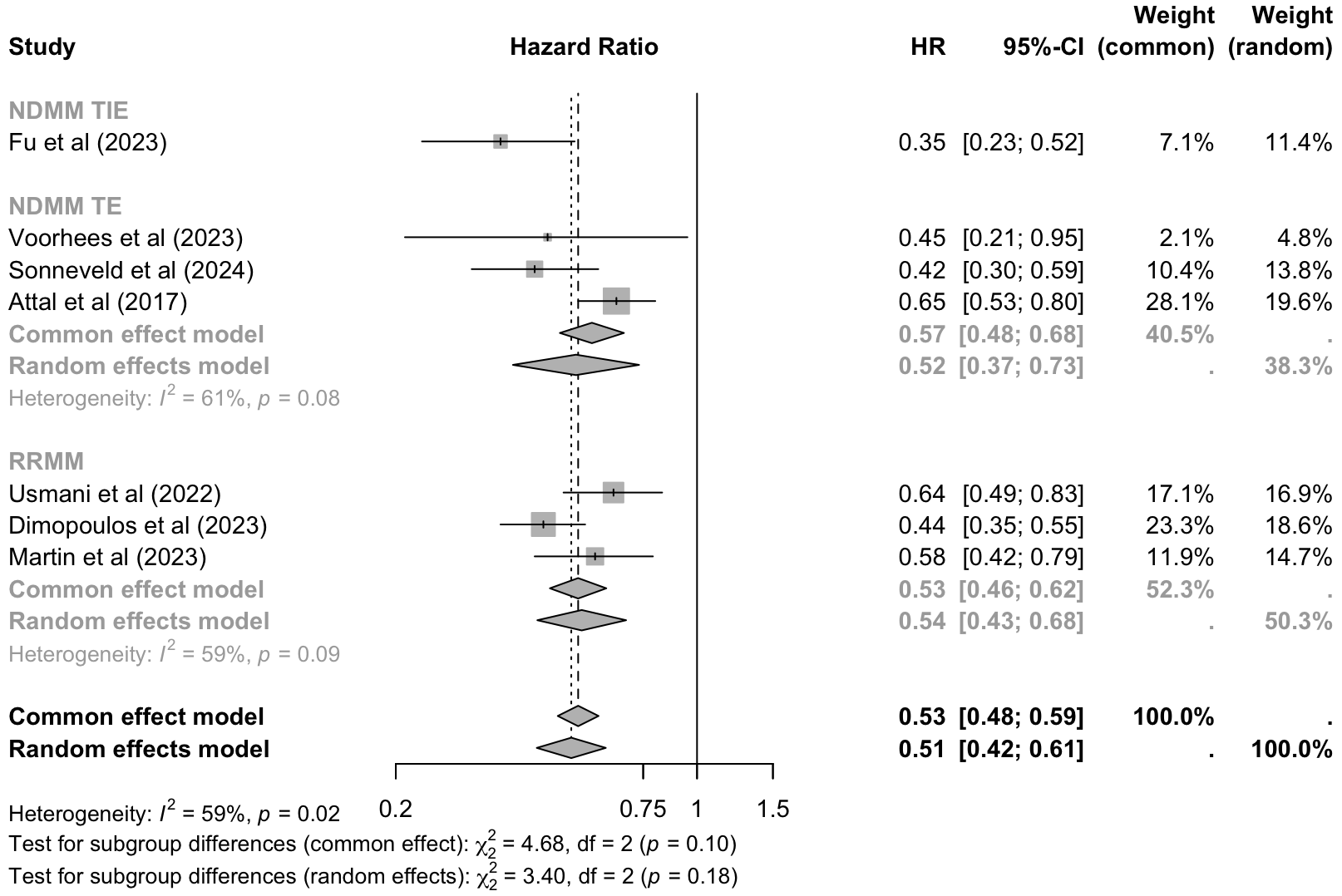


**Figure S60.** PFS (HR) pooled-estimate for the base-case analysis (by region)


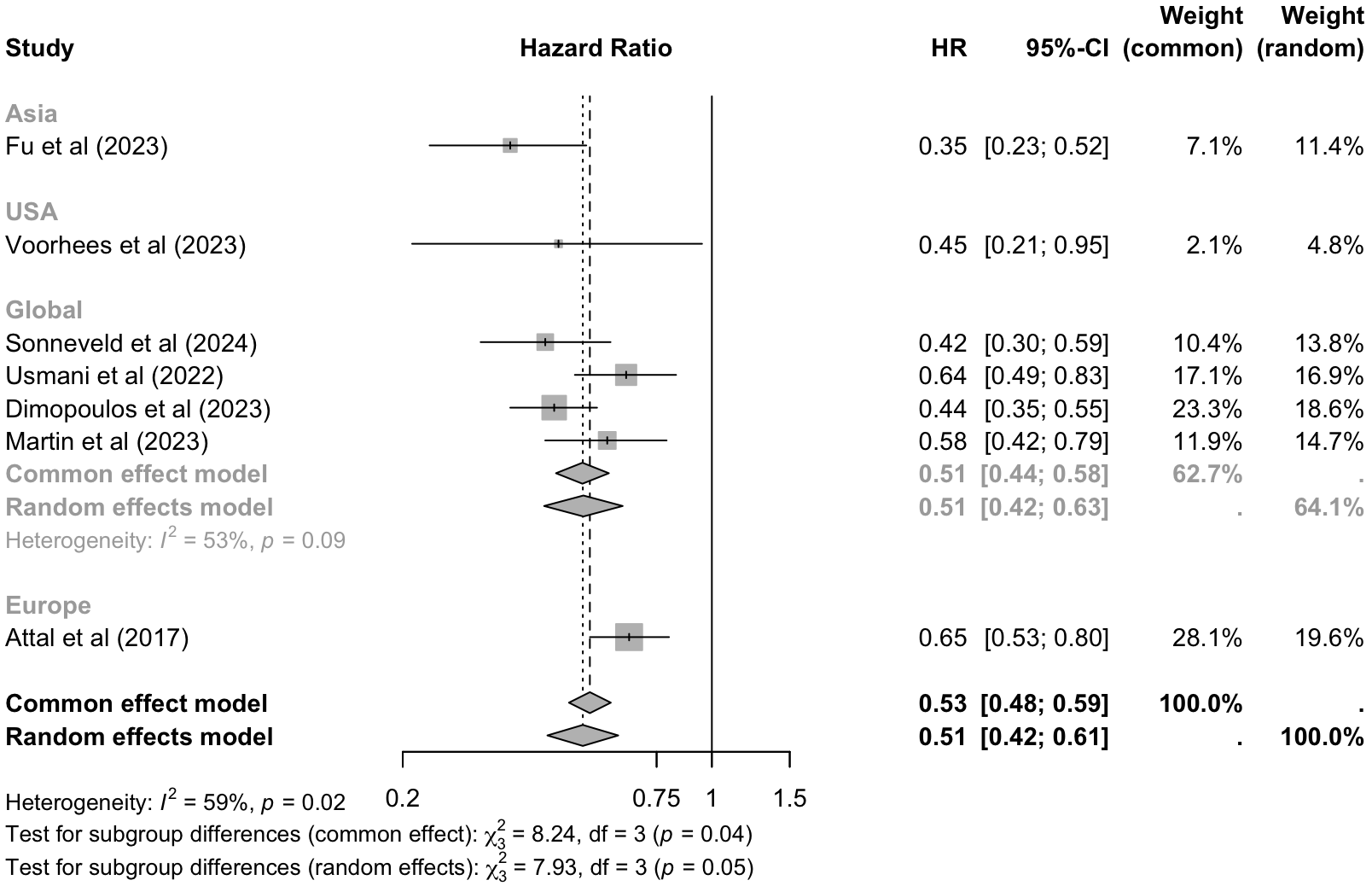


**Figure S61.** PFS (HR) pooled-estimate for the base-case analysis (by treatment)


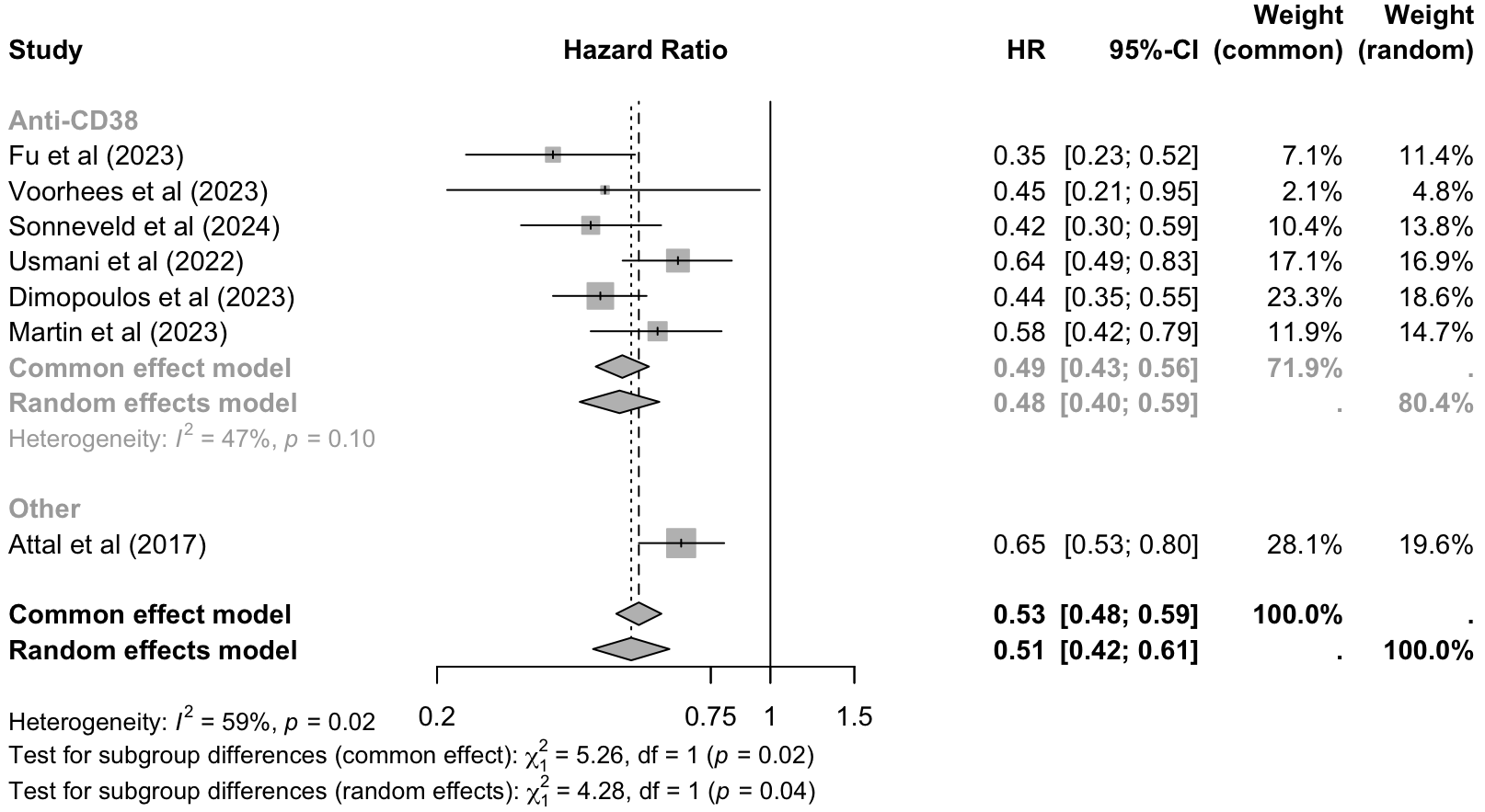


**Figure S62.** PFS (HR) pooled-estimate for the base case analysis (by adjustment)


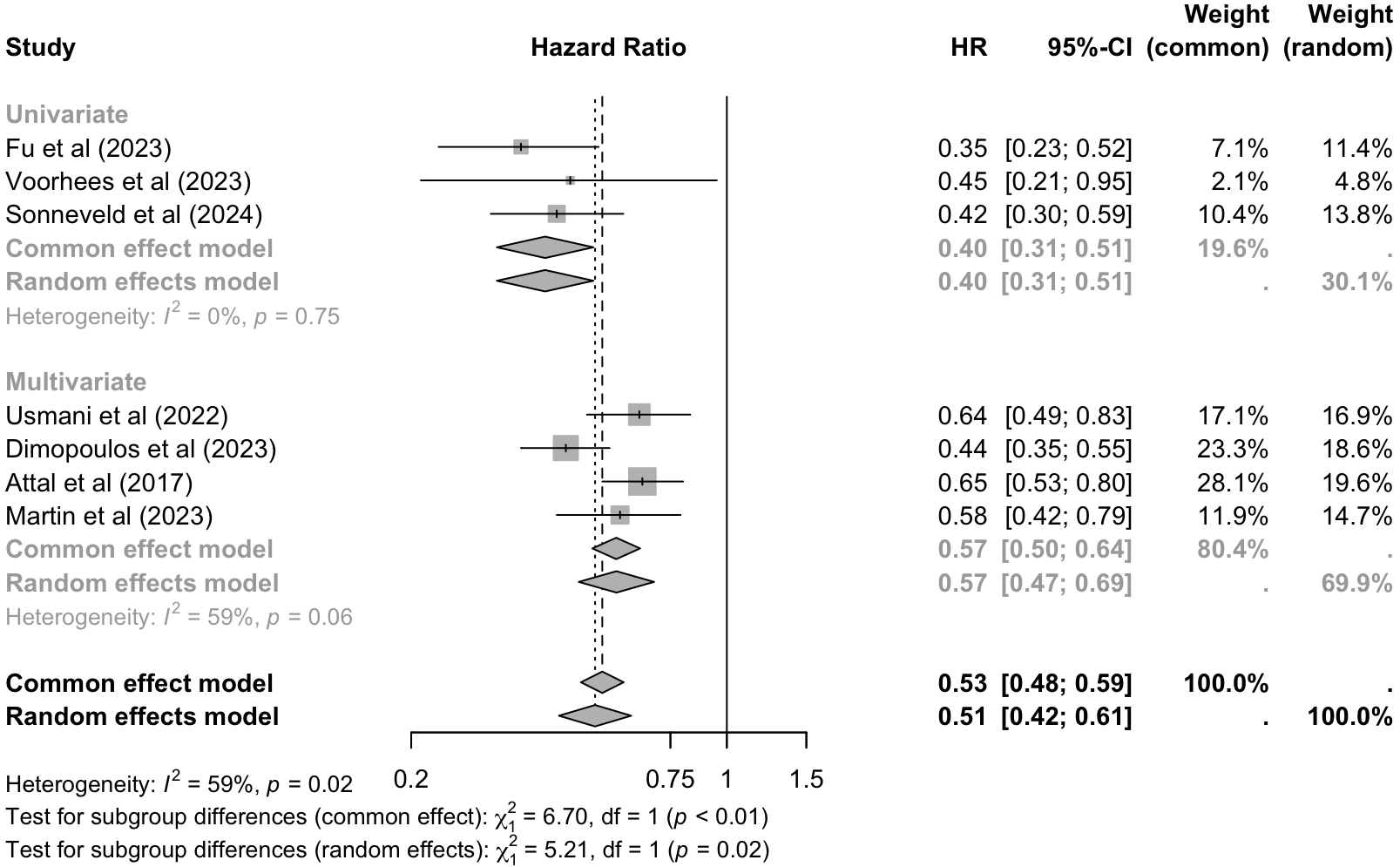


**Figure S63.** OS (HR) pooled-estimate for the base case analysis (by setting)


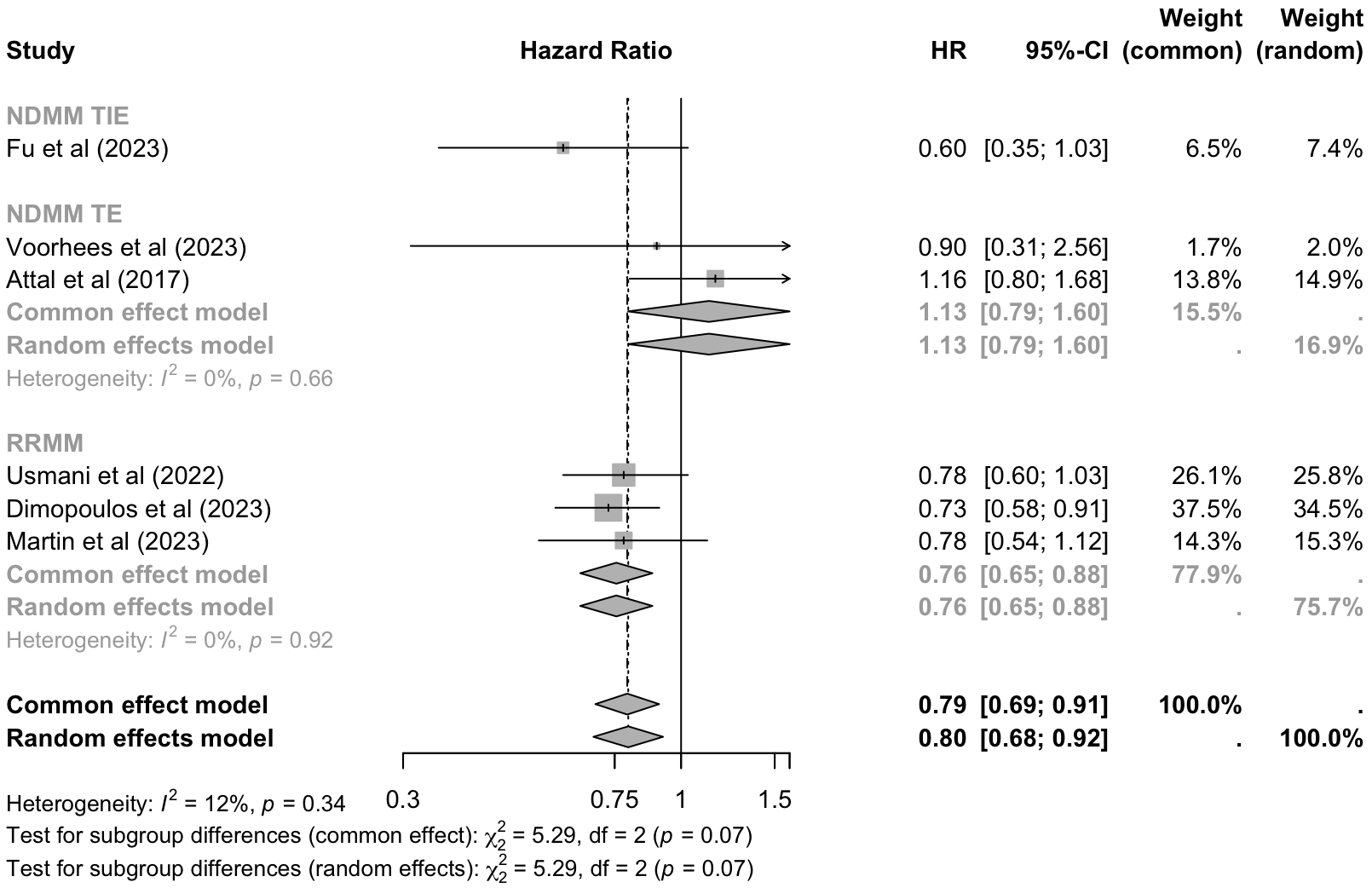


**Figure S64.** OS (HR) pooled-estimate for the base case analysis (by region)


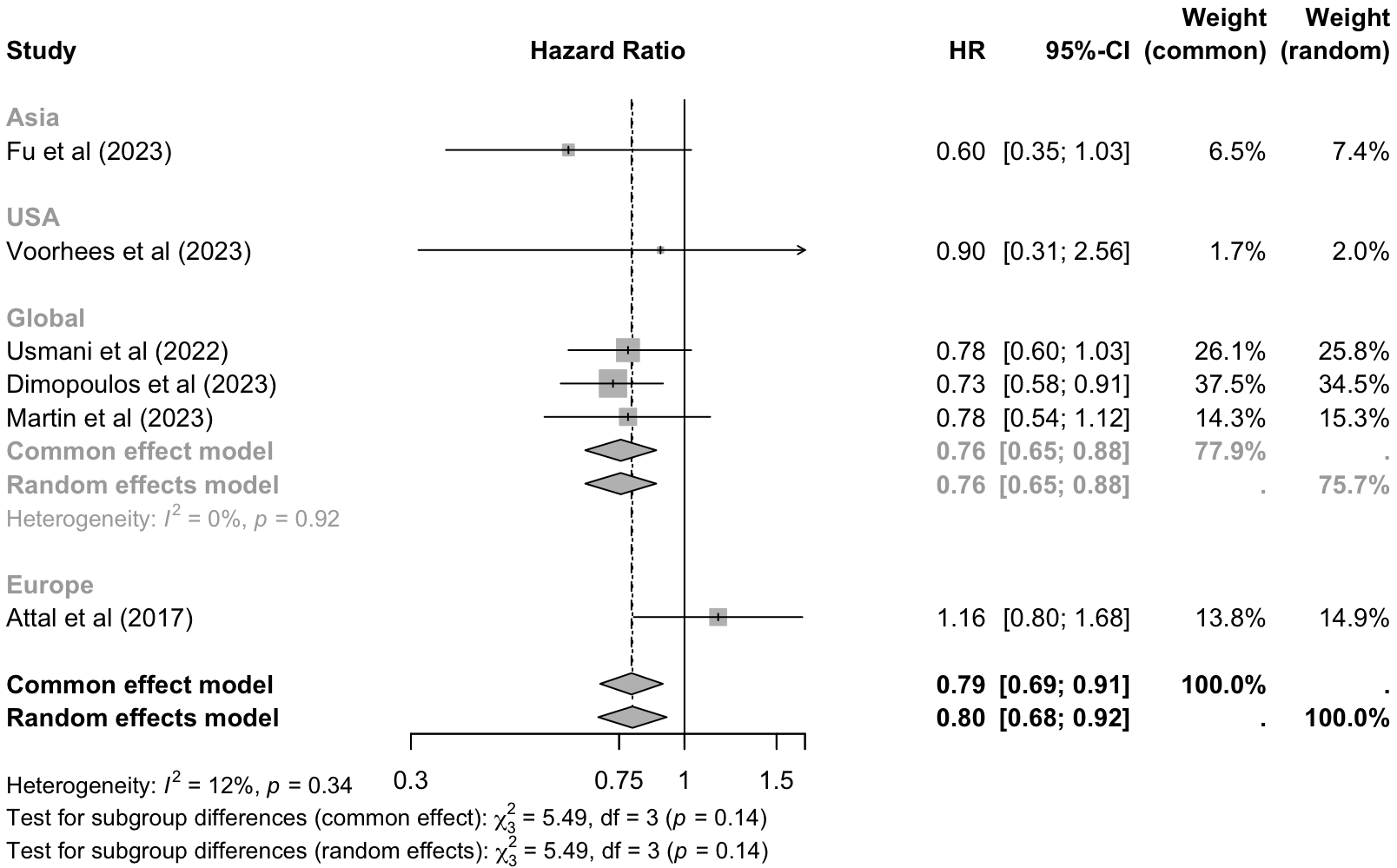


**Figure S65.** OS (HR) pooled-estimate for the base case analysis (by treatment)


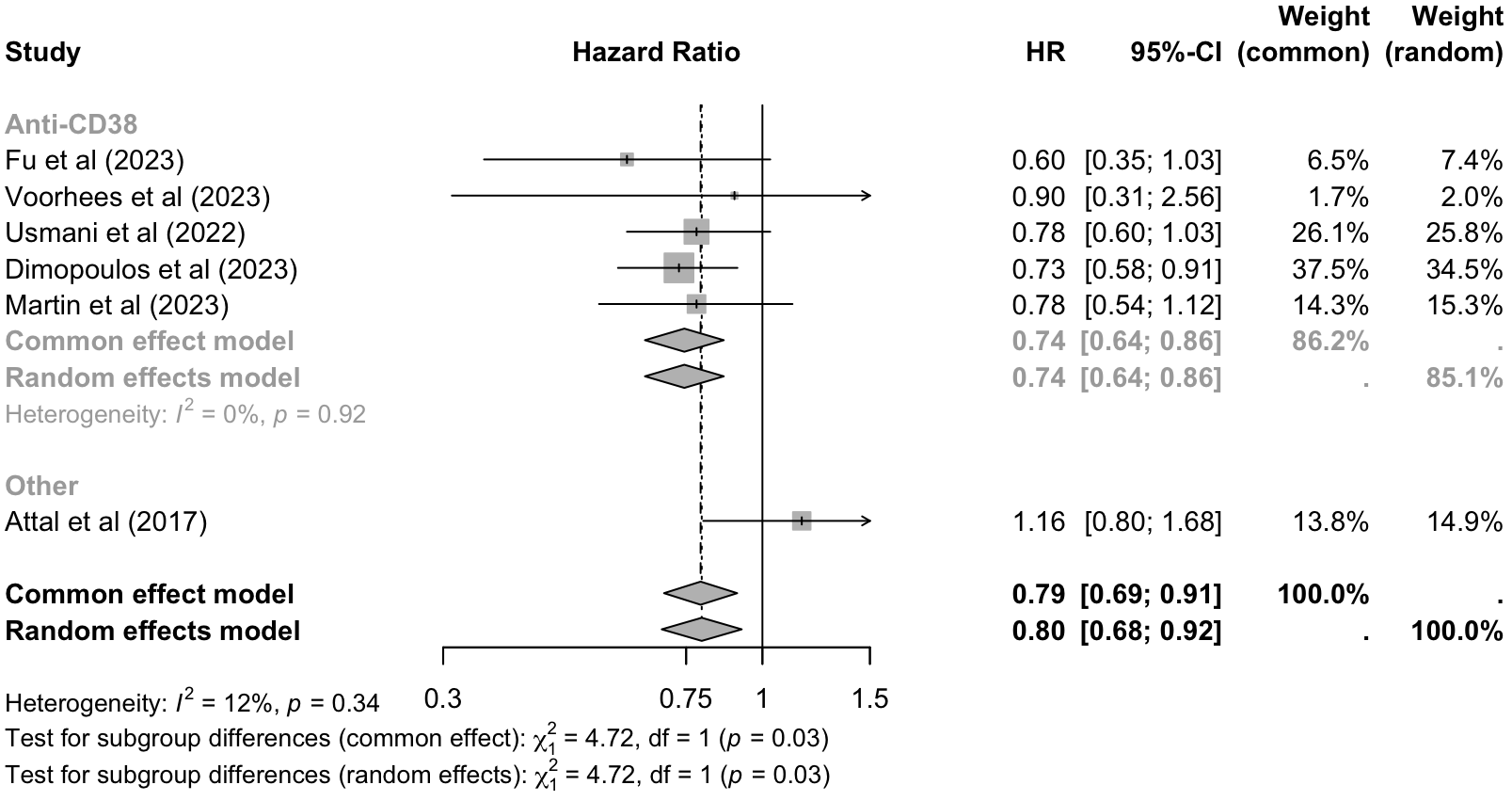


**Figure S66.** OS (HR) pooled-estimate for the base case analysis (by adjustment)


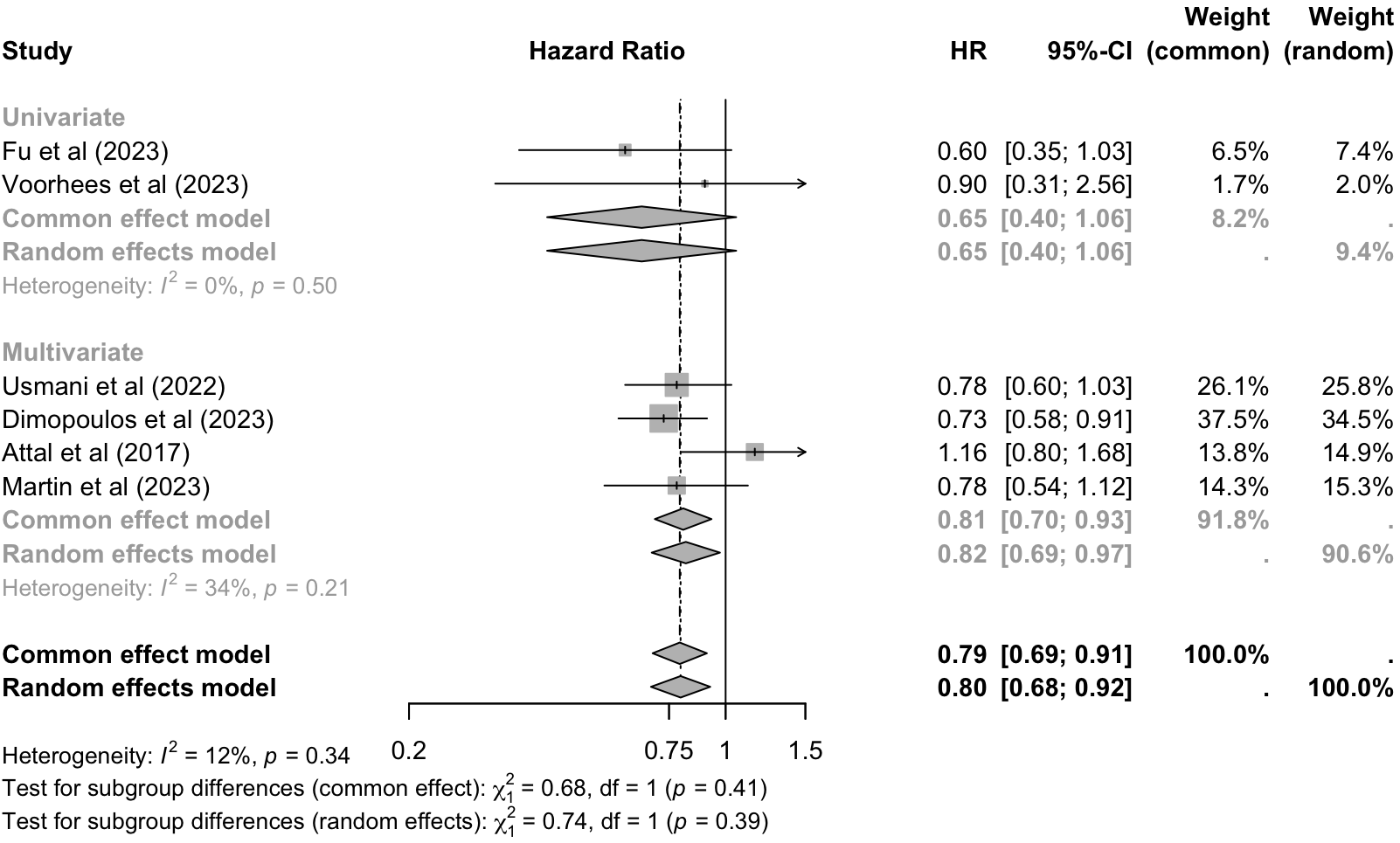


**Figure S67.** Funnel plot for MRD negativity ORs**
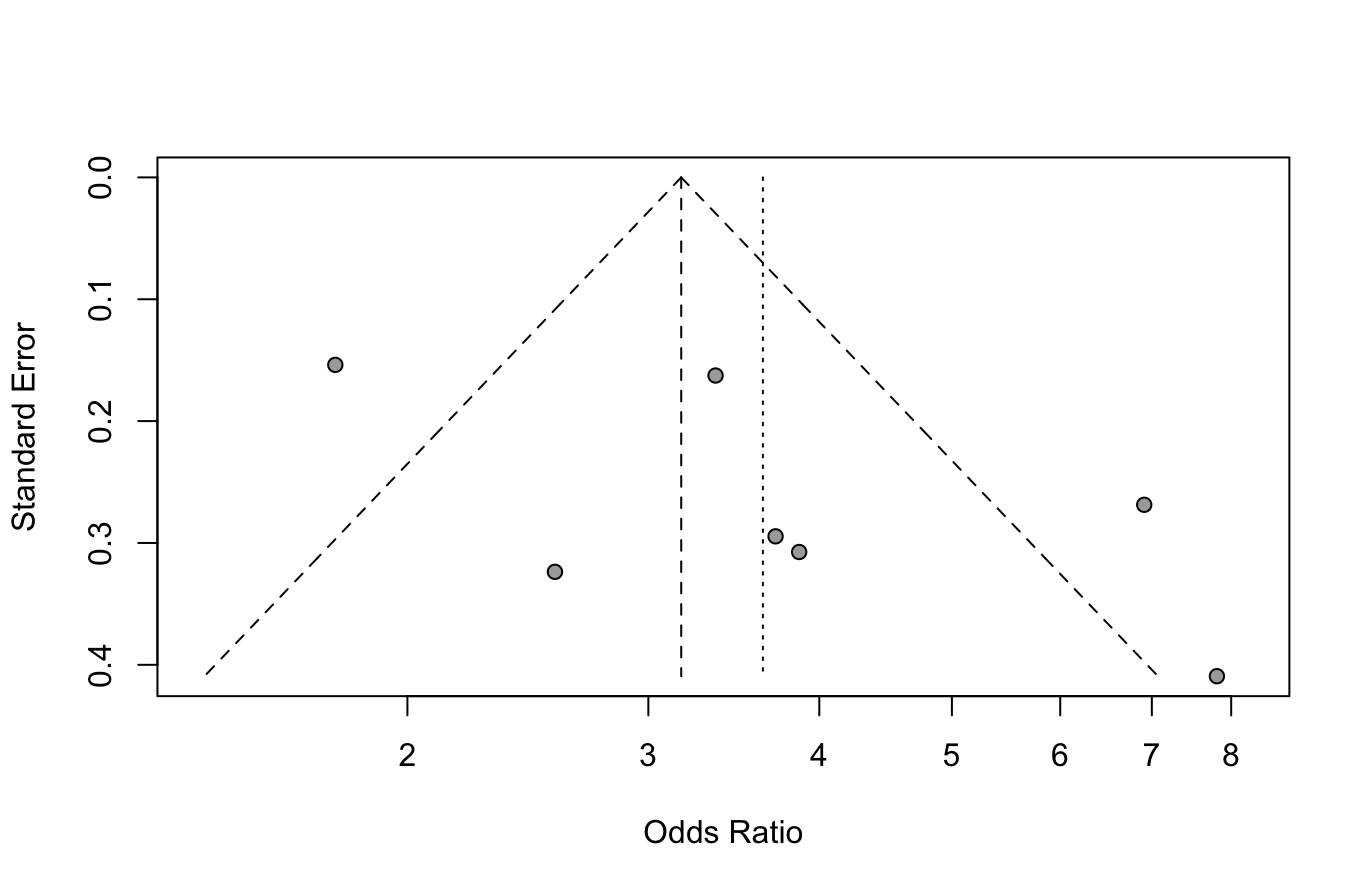
**

**Figure S68.** Funnel plot for PFS HRs


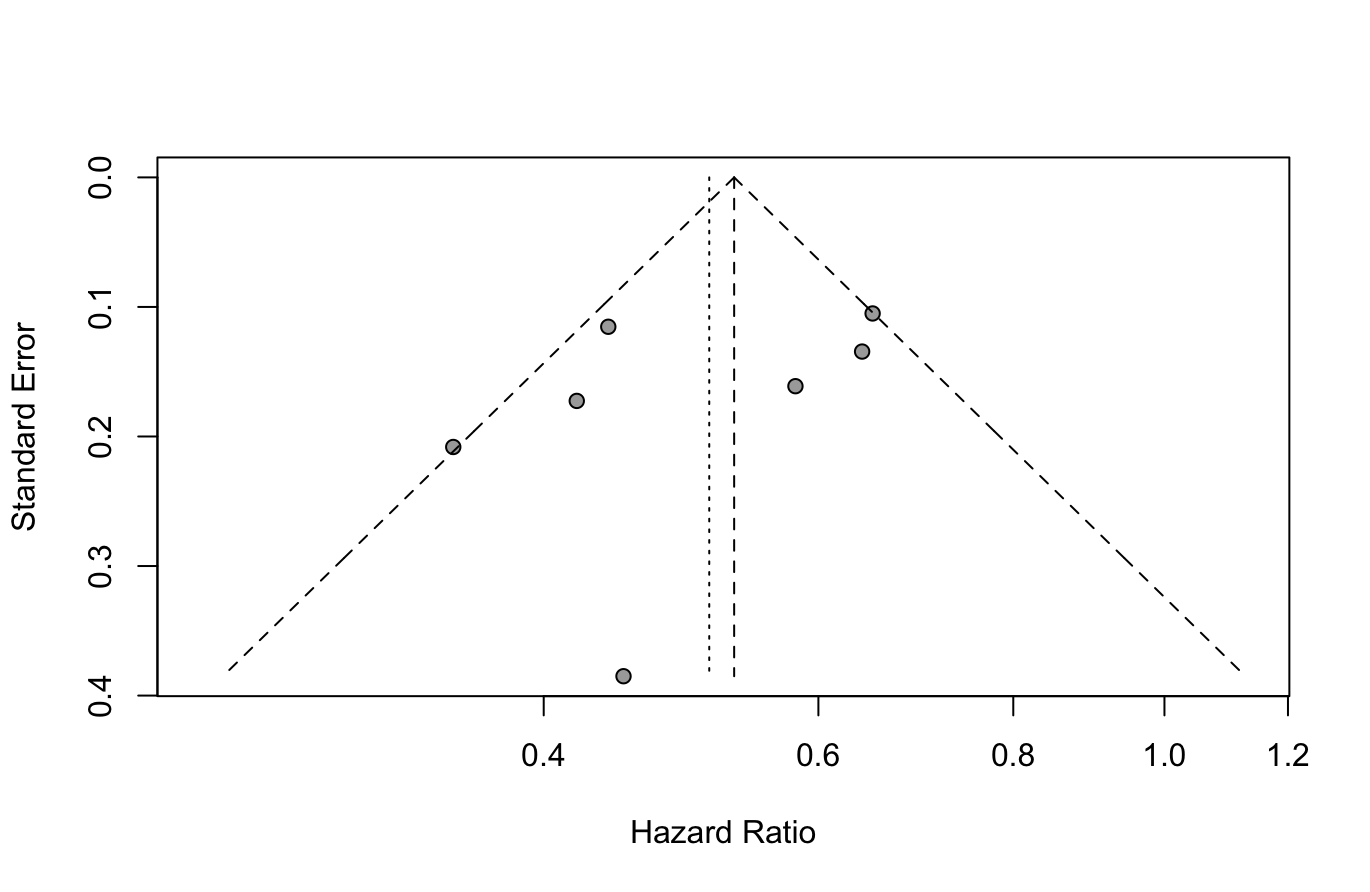


**Figure S69.** Funnel plot for OS HRs


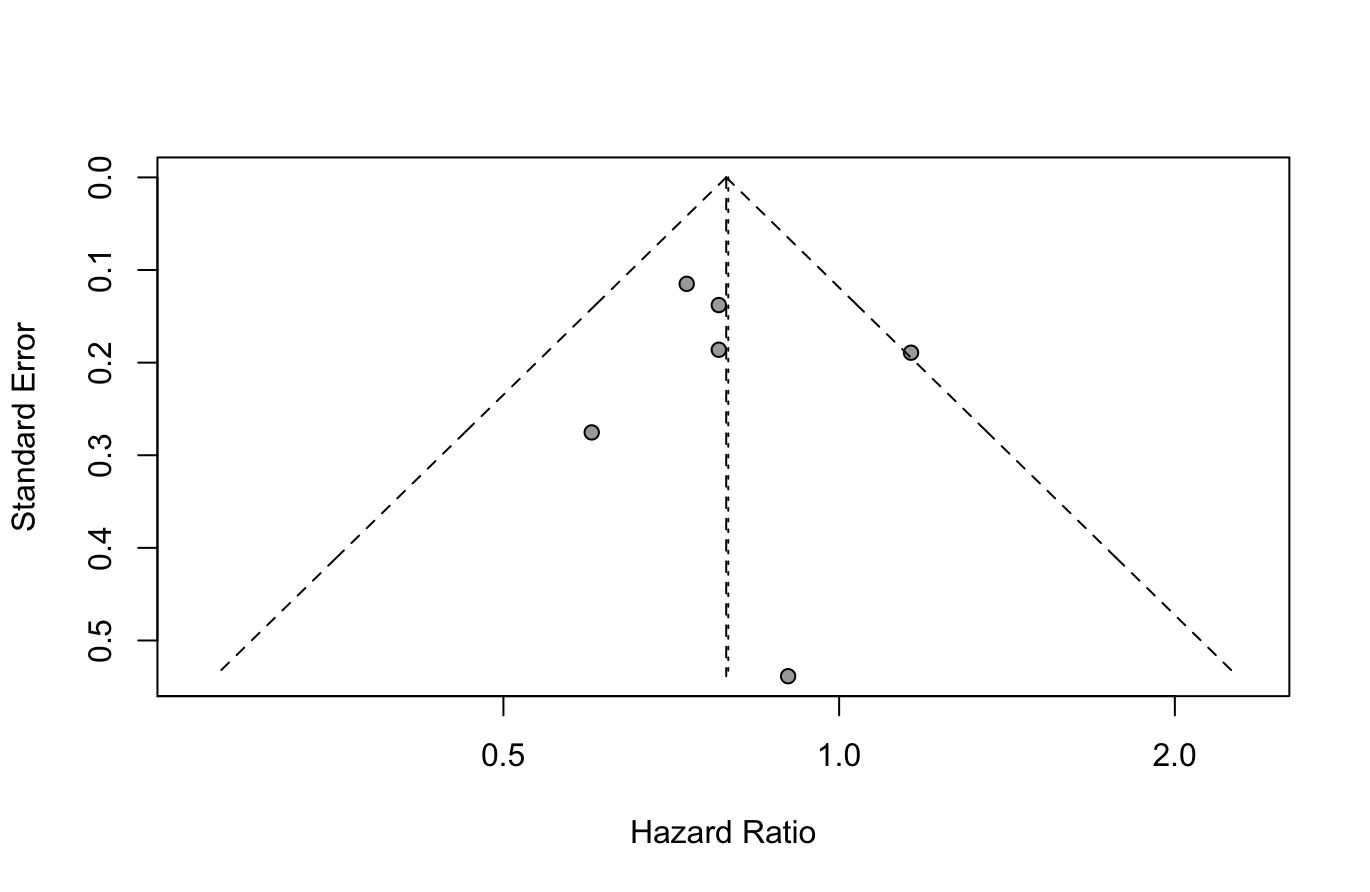


*Sustained MRD negativity for 12 months*

**Figure S70.** Sustained MRD negativity for 12 months (OR) pooled-estimate (by setting)


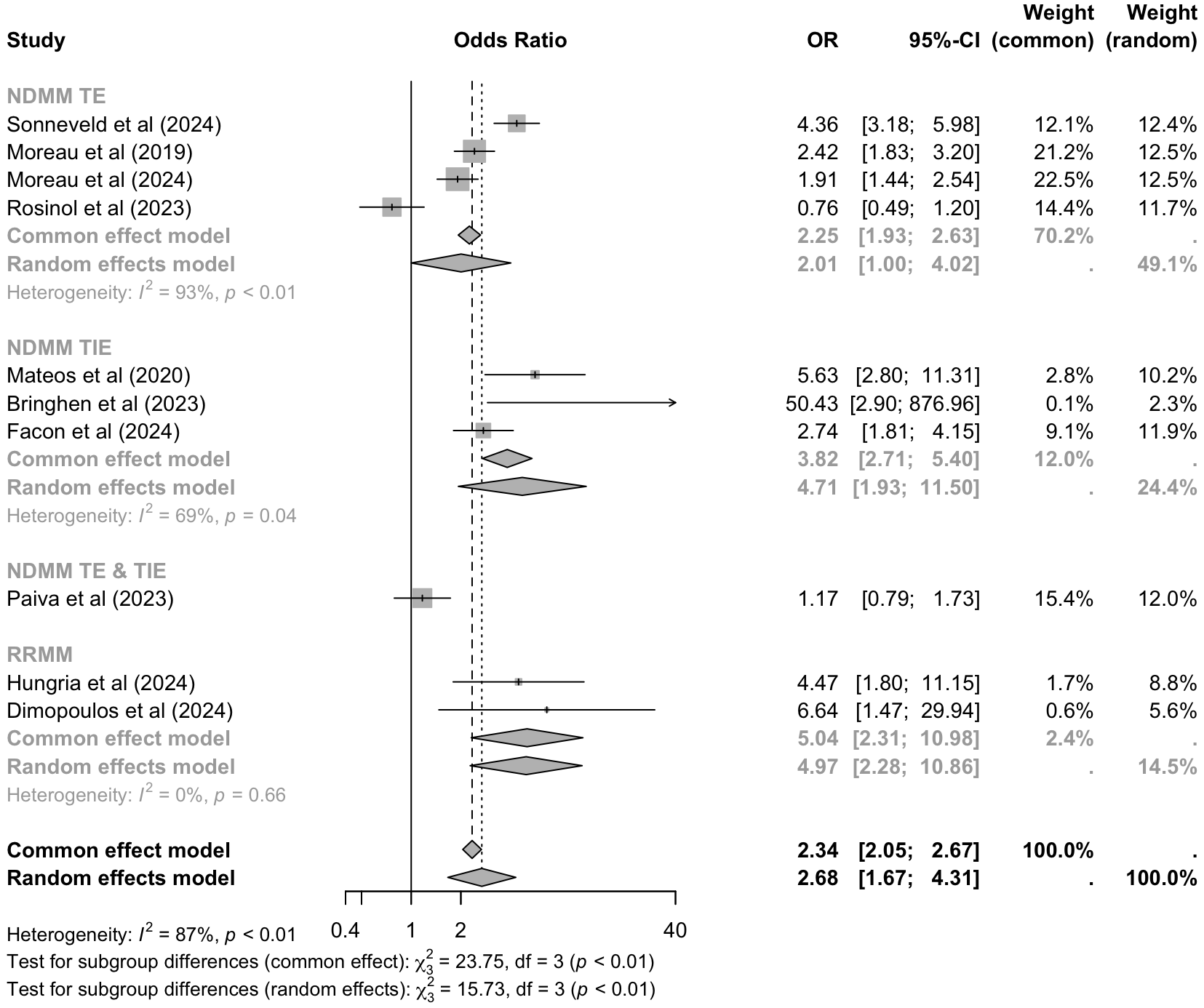


**Figure S71.** Sustained MRD negativity for 12 months (OR) pooled-estimate (by region)


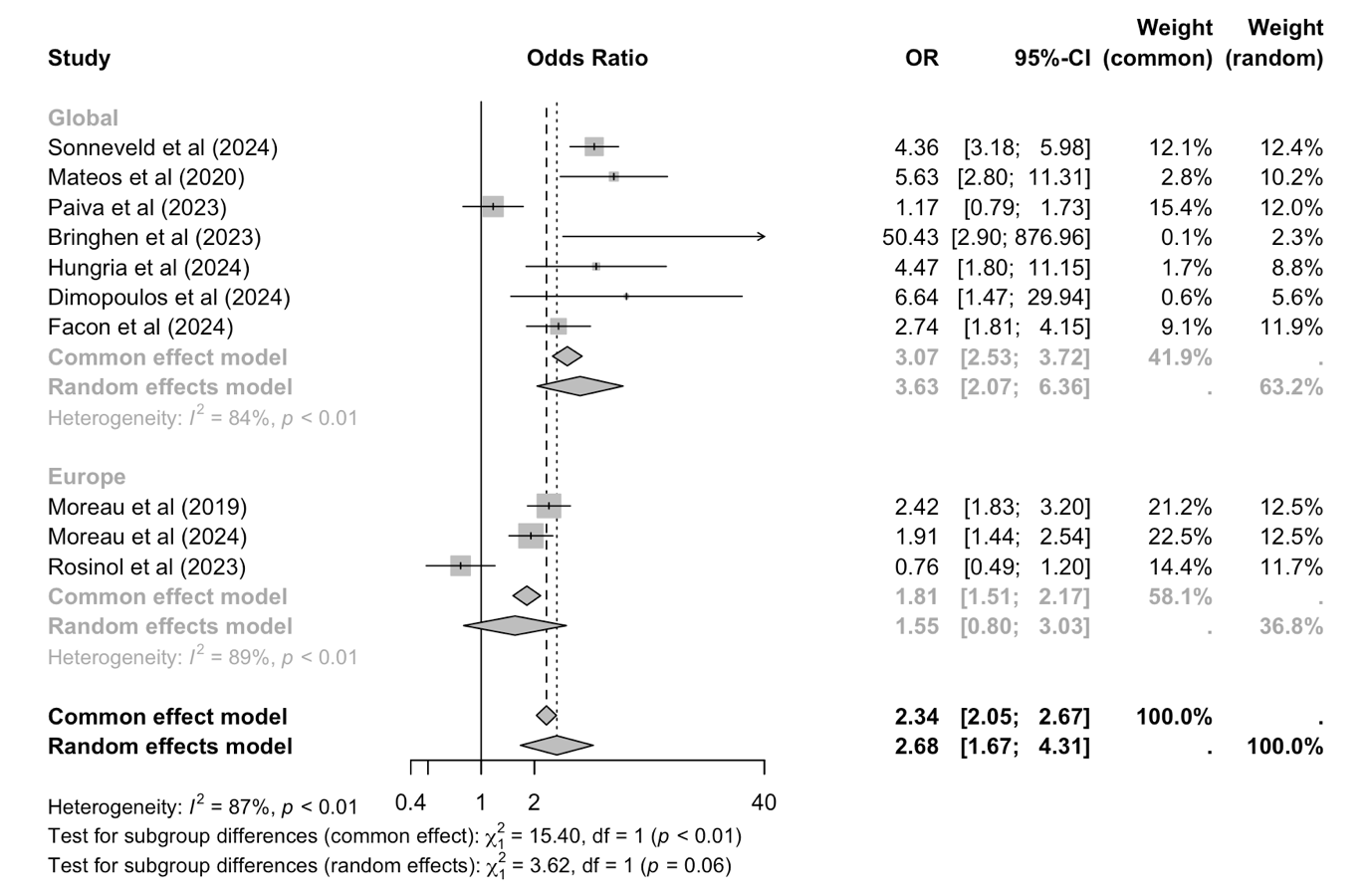


**Figure S72.** Sustained MRD negativity for 12 months (OR) pooled-estimate (by treatment)


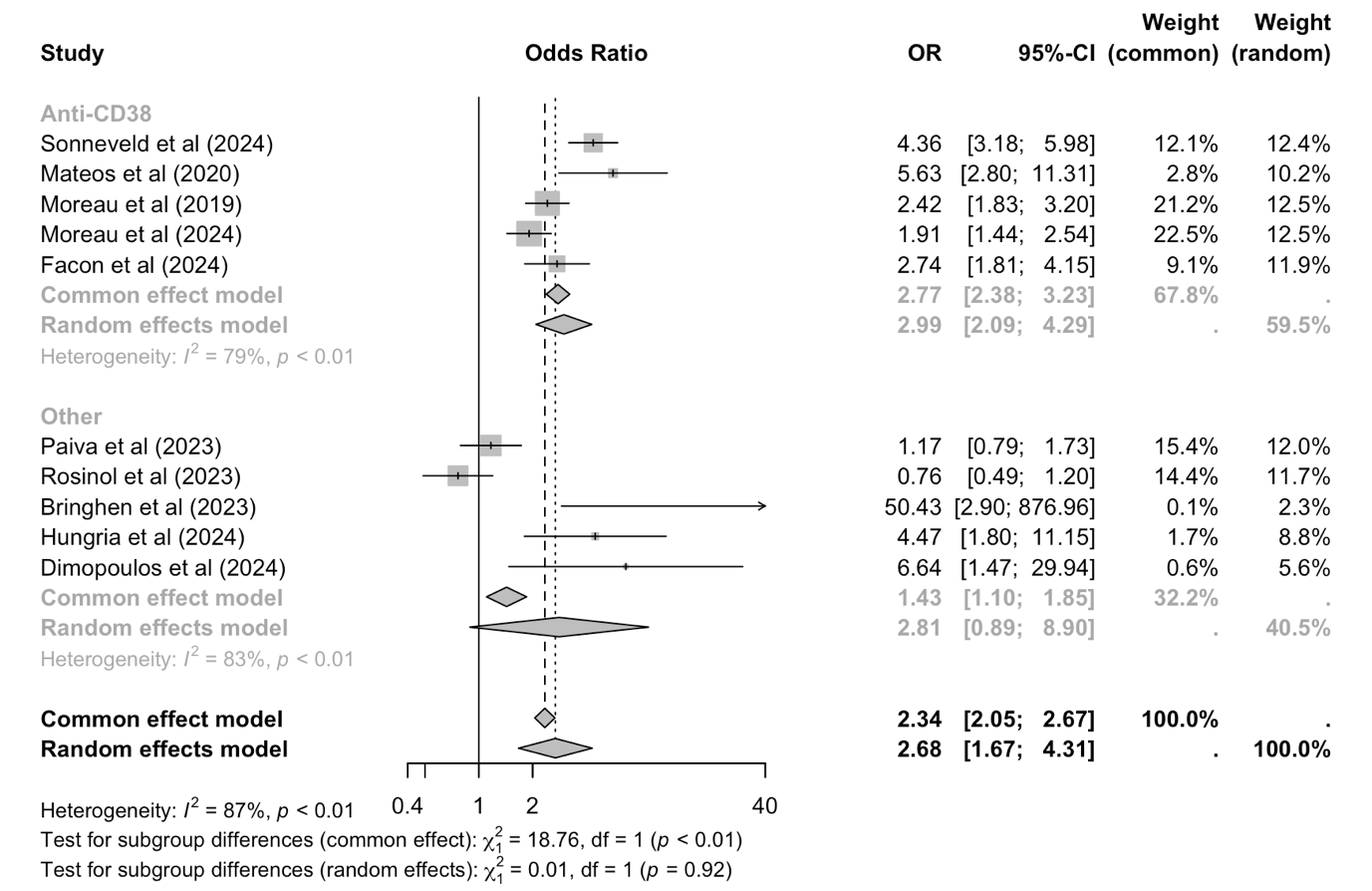


**Figure S73.** PFS (HR) pooled-estimate for the base-case analysis (by setting)


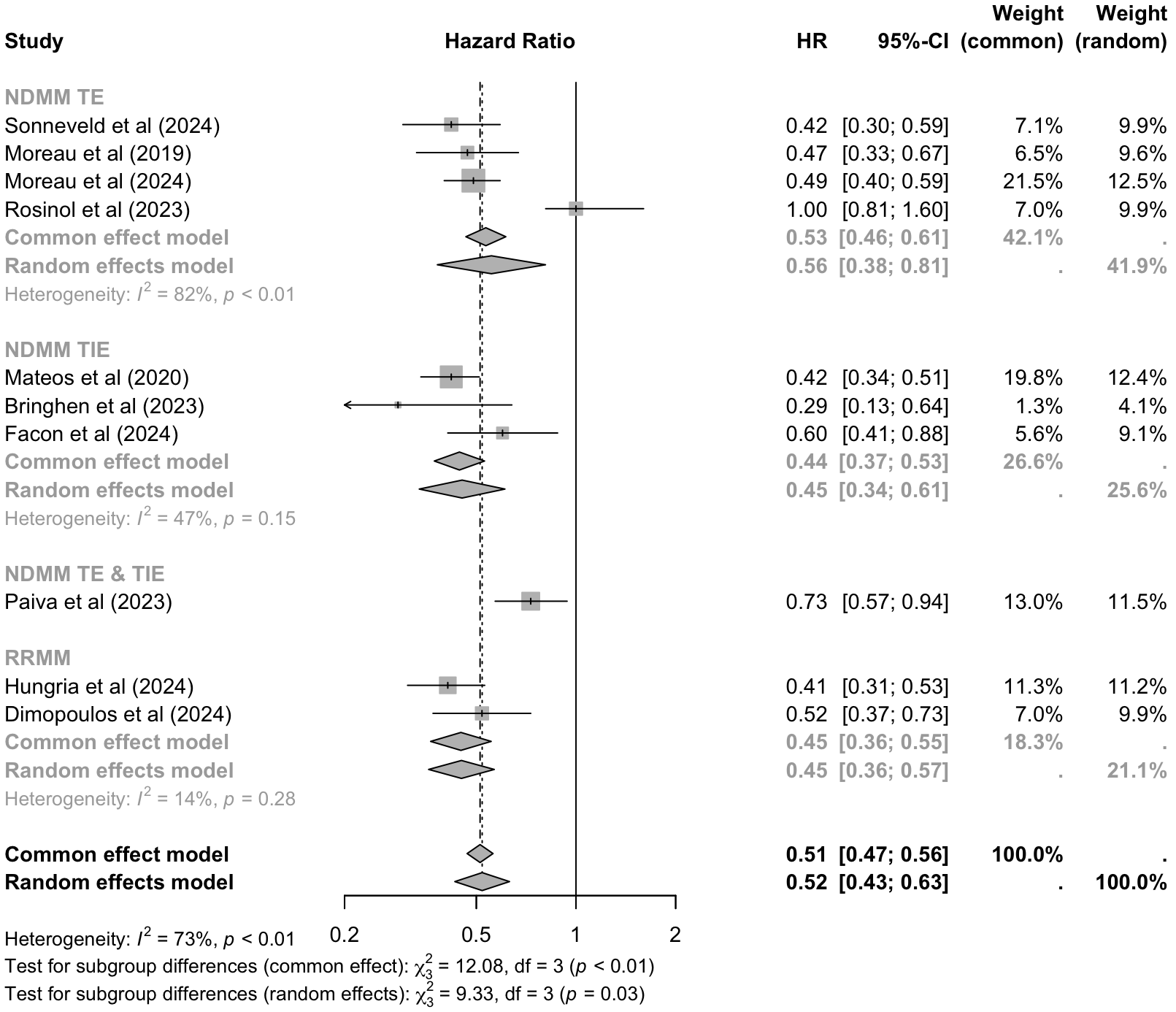


**Figure S74.** PFS (HR) pooled-estimate for the base-case analysis (by region)


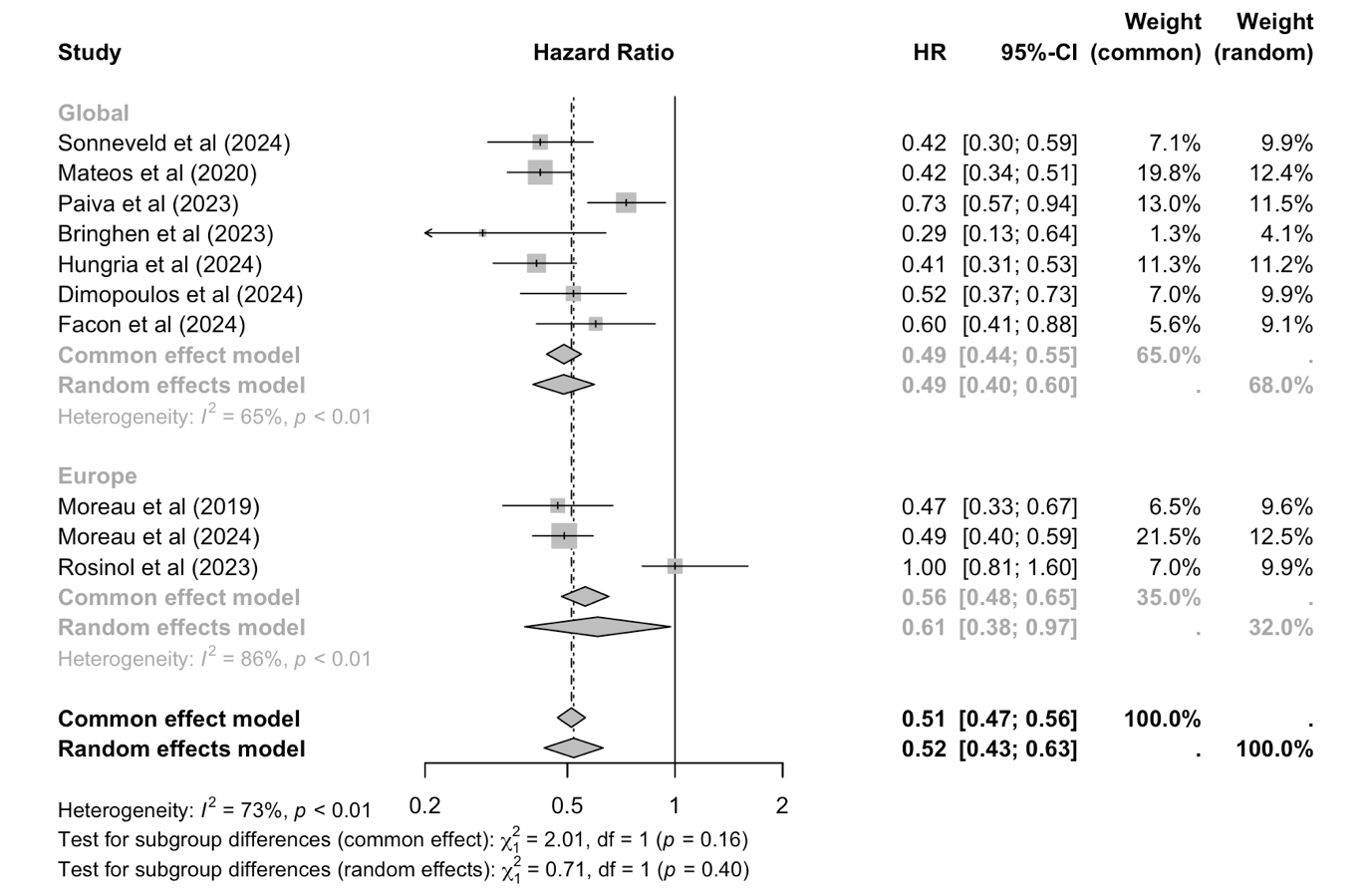


**Figure S75.** PFS (HR) pooled-estimate for the base-case analysis (by treatment)


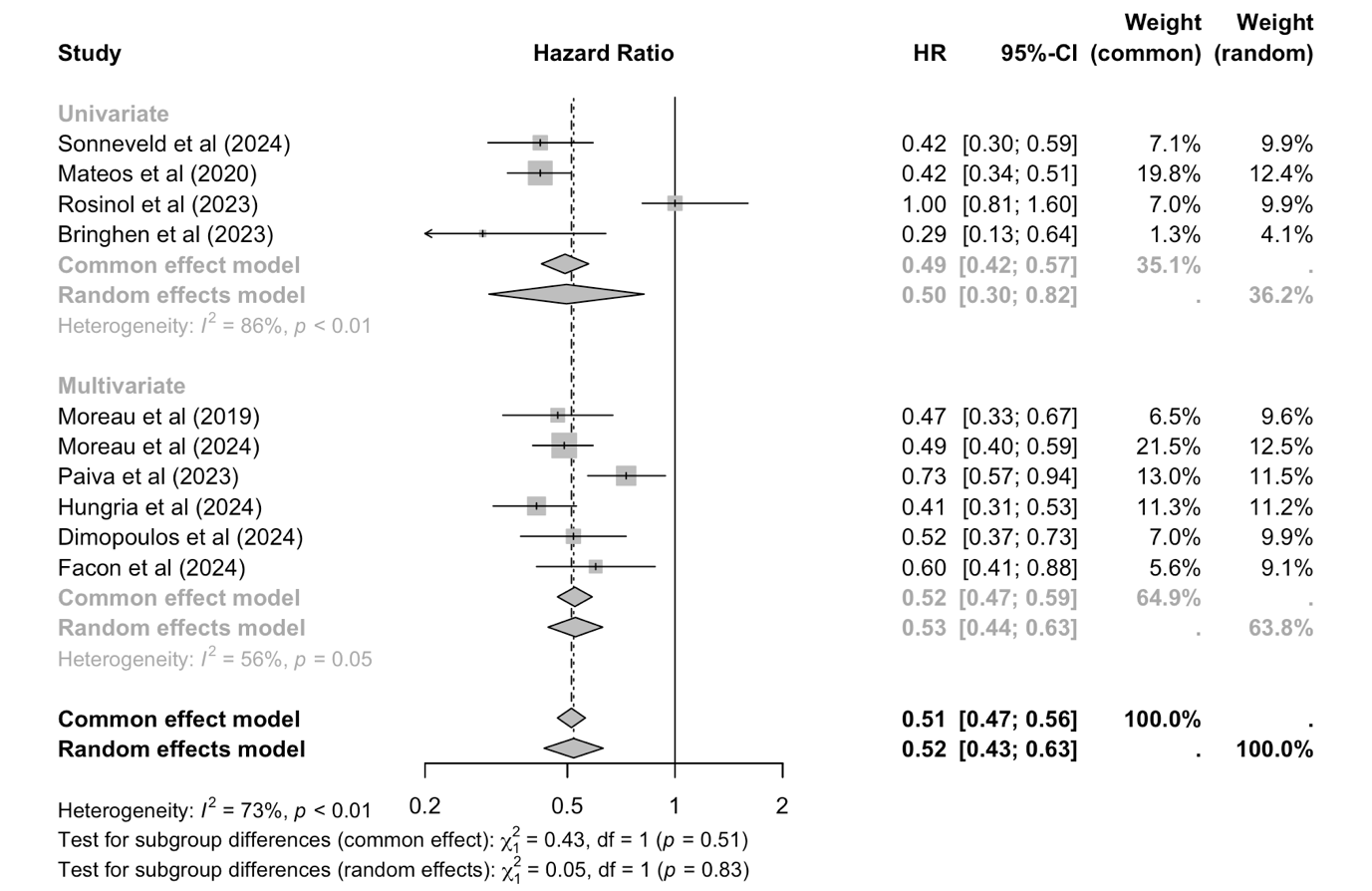


**Figure S76.** PFS (HR) pooled-estimate for the base-case analysis (by adjustment)


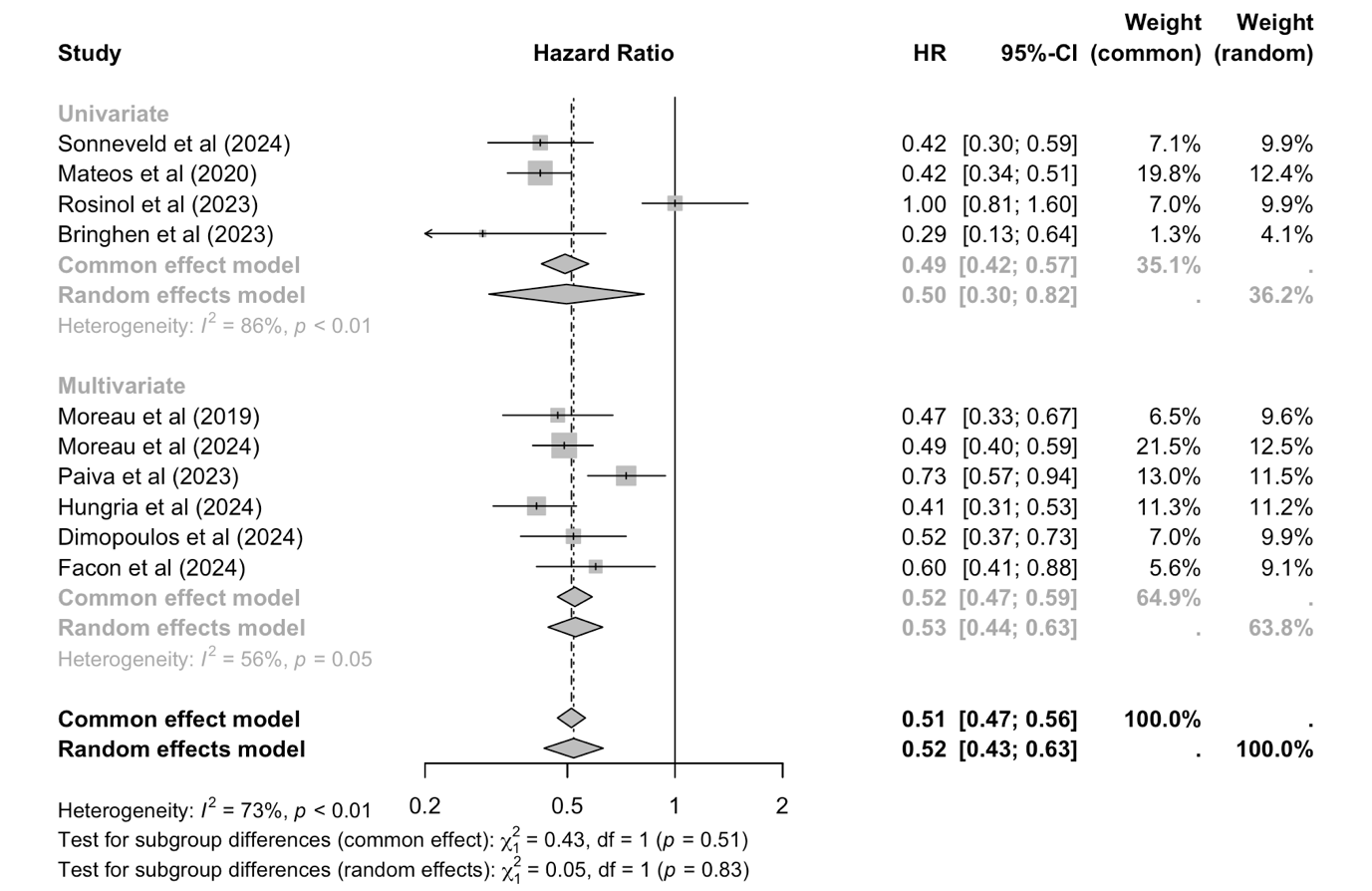


**Figure S77.**


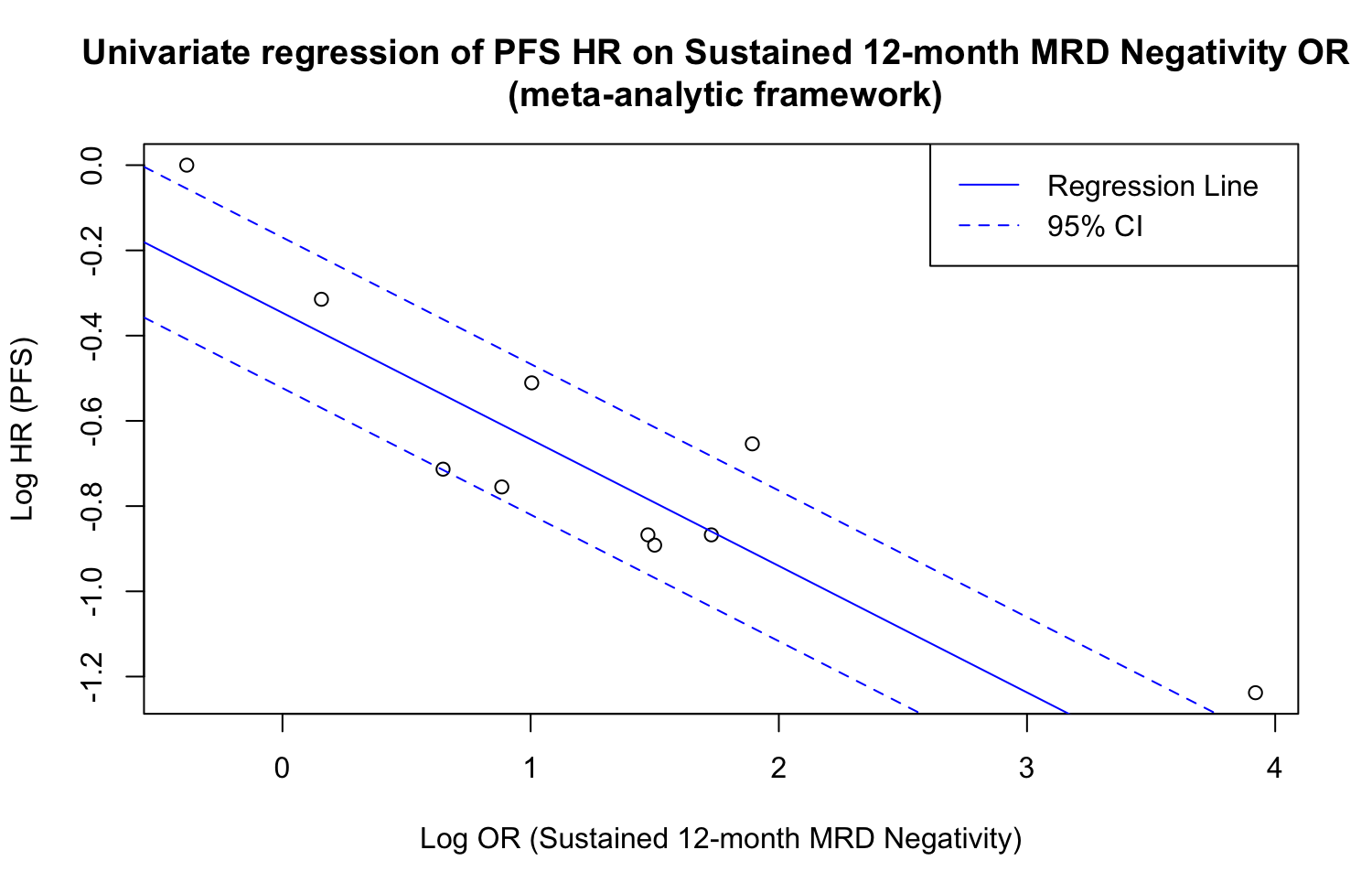


**Figure S78.** Funnel plot for sustained MRD negativity for 12-months ORs


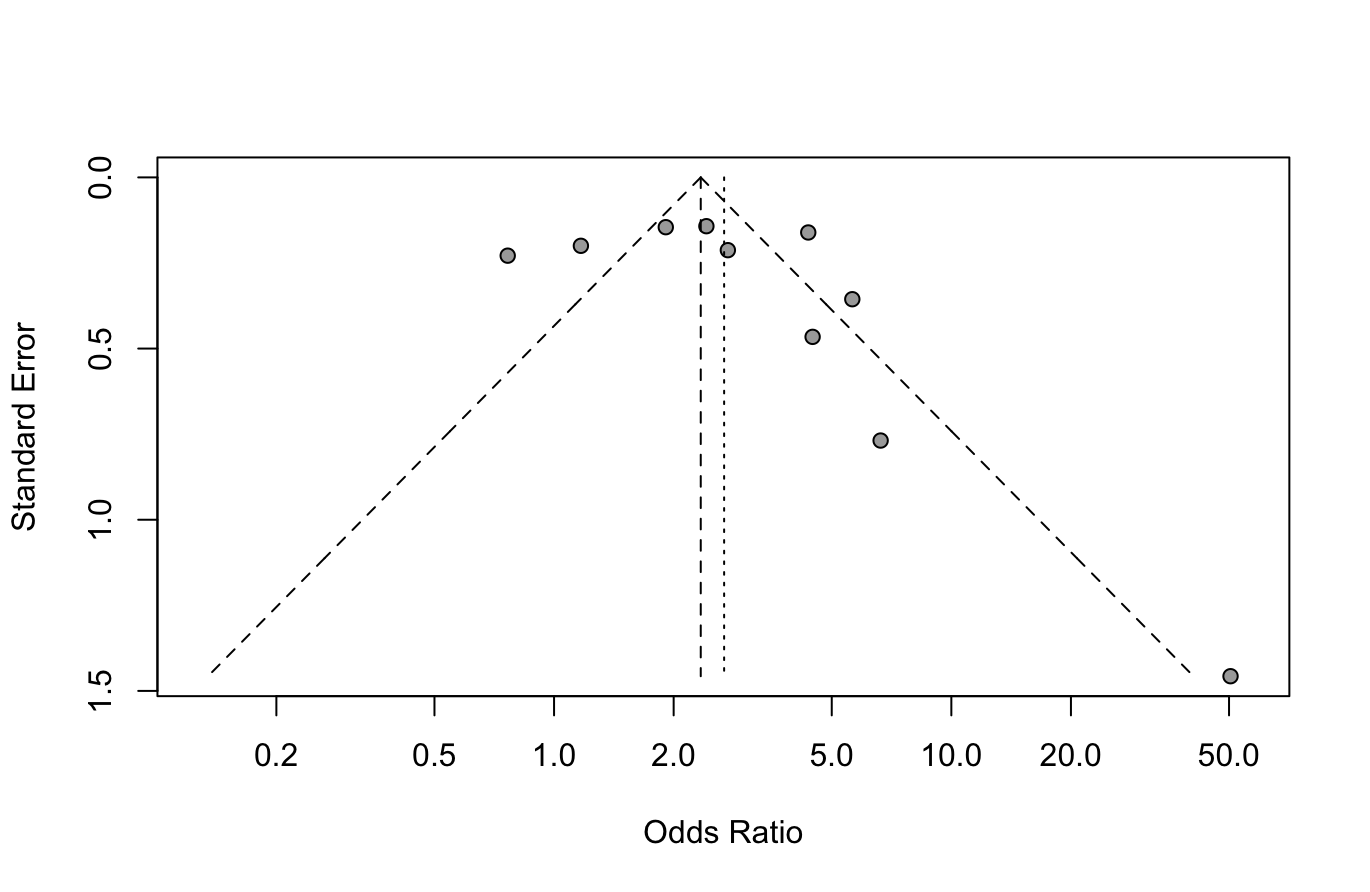


**Figure S79.** Funnel plot for PFS HRs


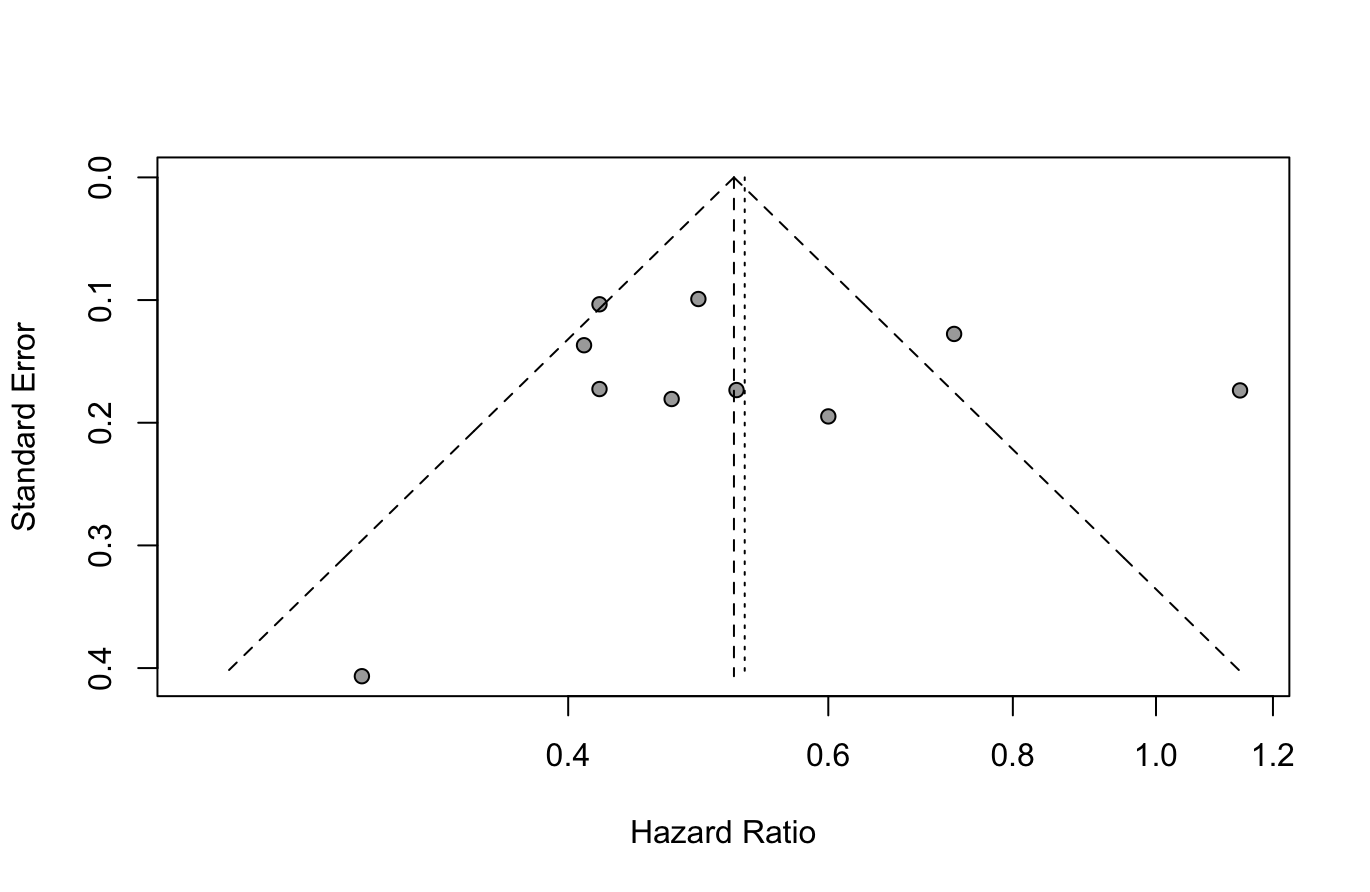


*TTNT vs. MRD negativity analysis*

**Figure S80.** MRD negativity (OR) pooled-estimate for the base-case analysis (by setting)


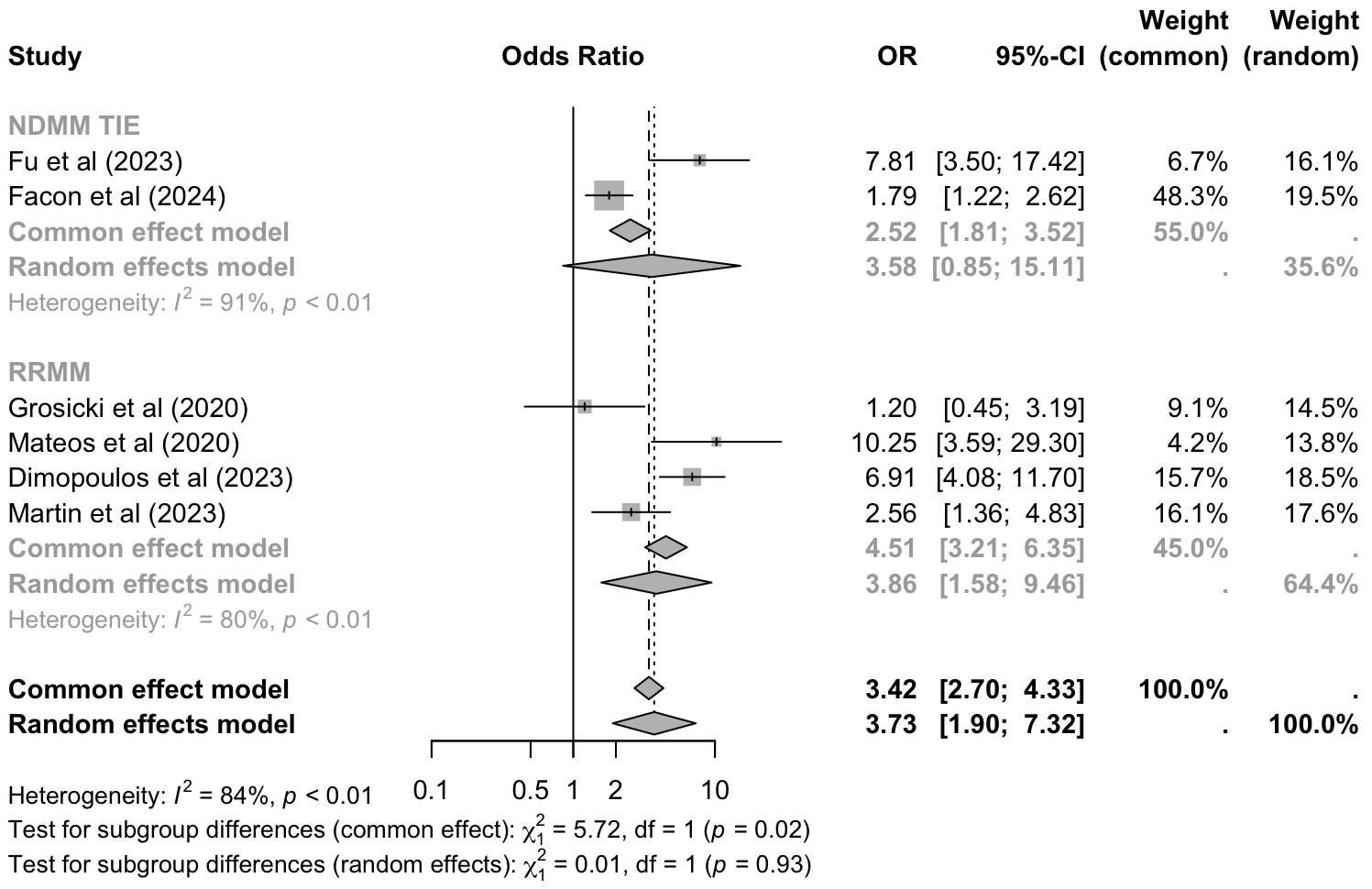


**Figure S81.** MRD negativity (OR) pooled-estimate for the base-case analysis (by region)


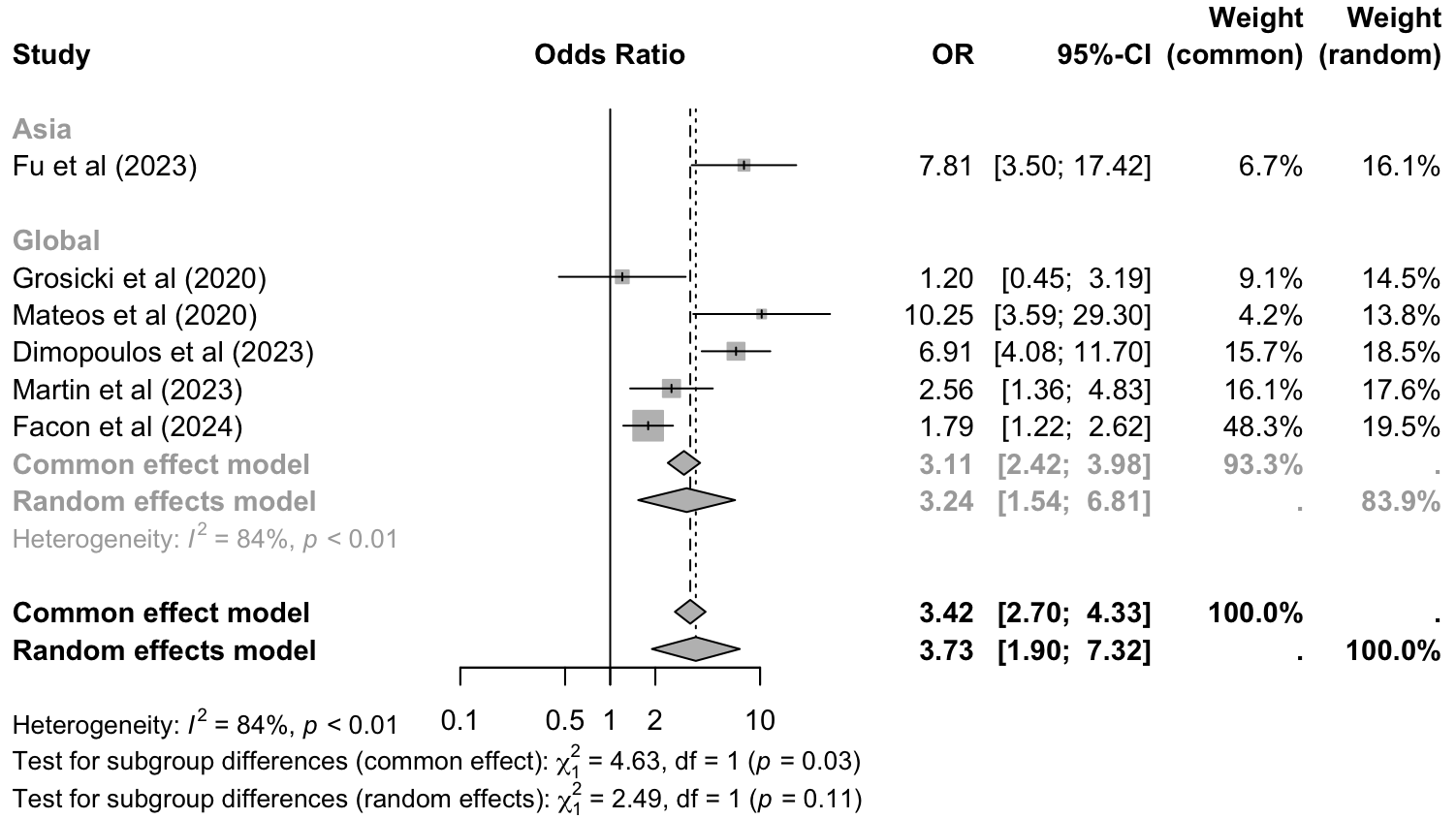


**Figure S82.** MRD negativity (OR) pooled-estimate for the base-case analysis (by treatment)


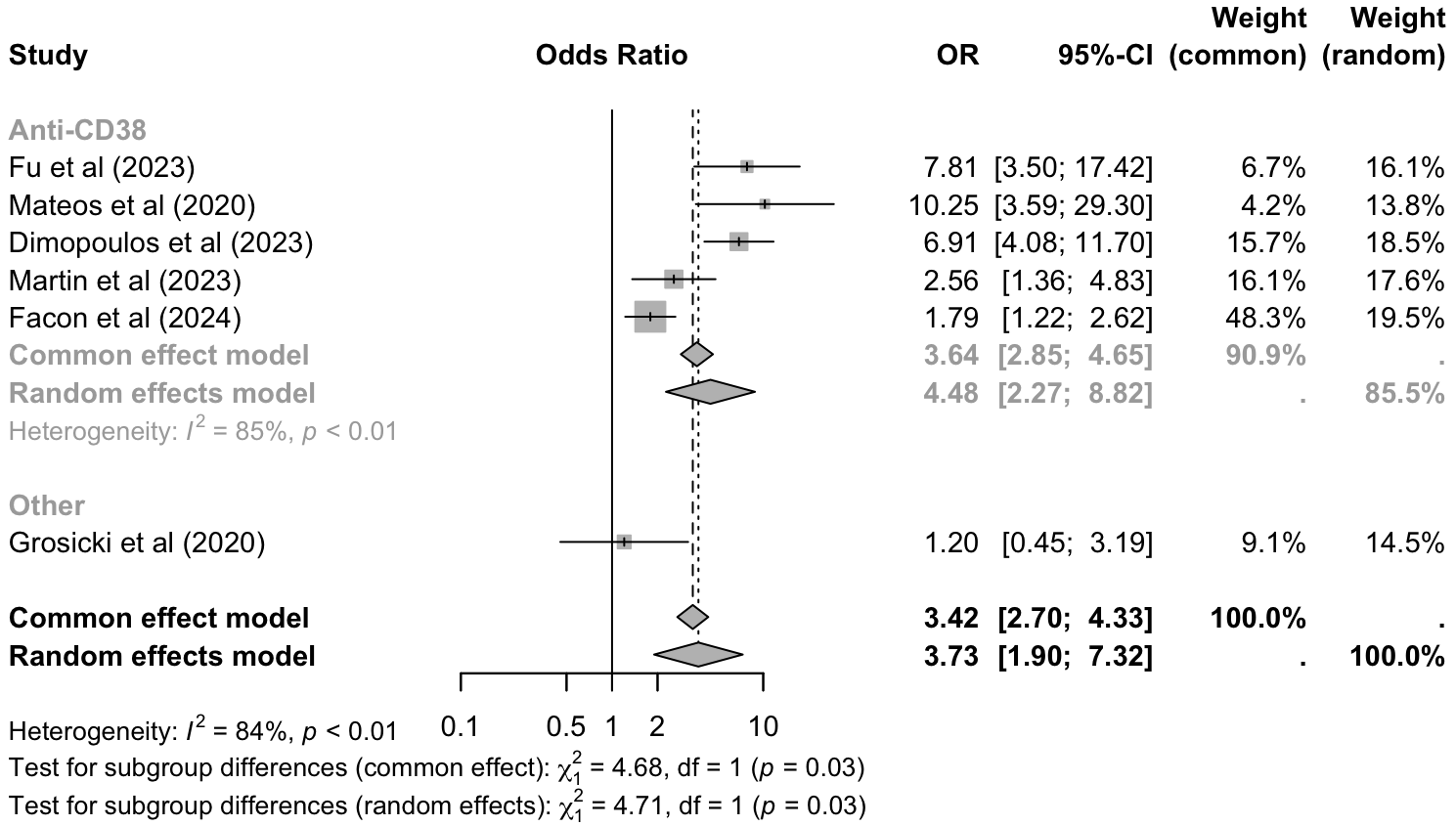


**Figure S83.** TTNT (HR) pooled-estimate for the base-case analysis (by setting)


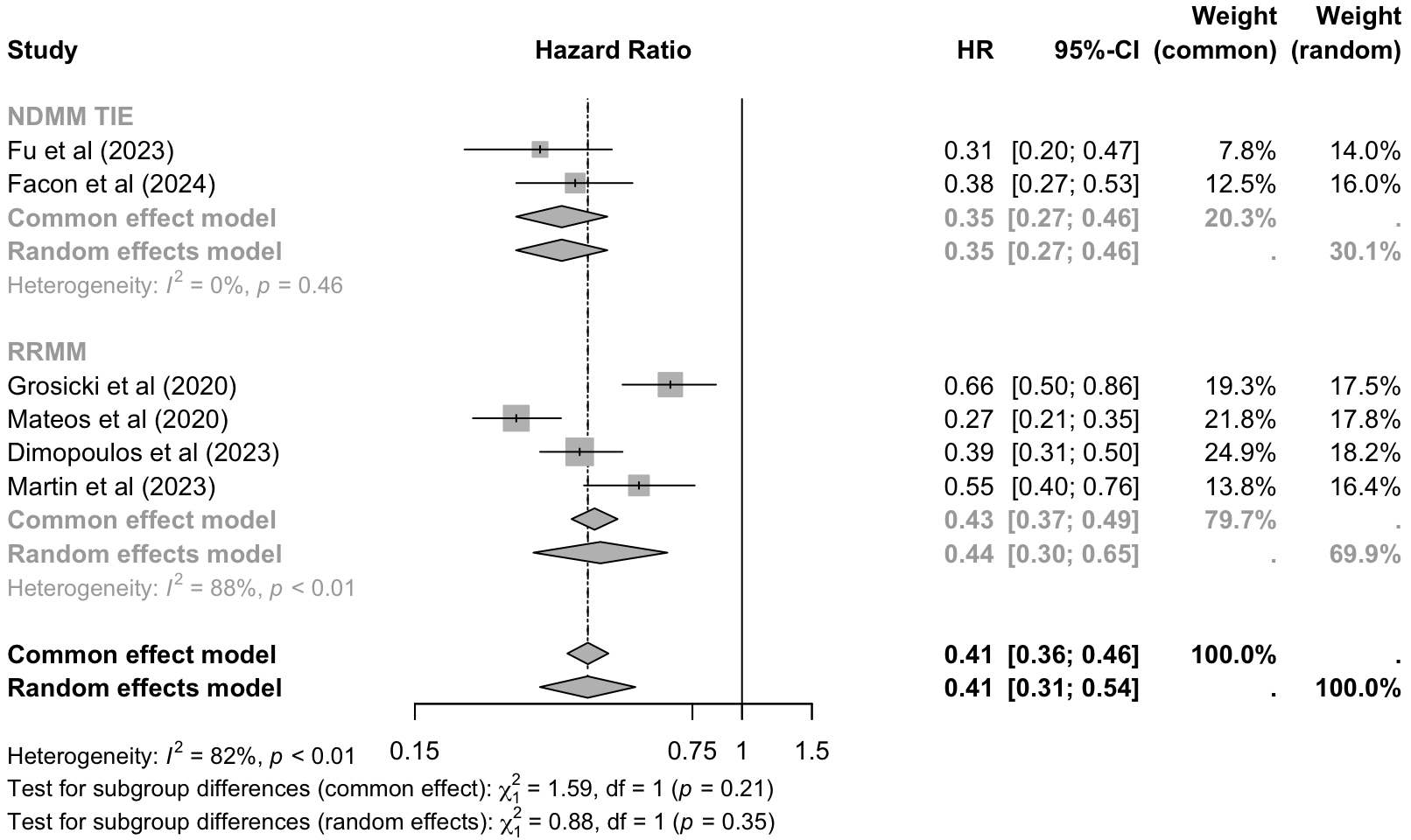


**Figure S84.** TTNT (HR) pooled-estimate for the base-case analysis (by region)


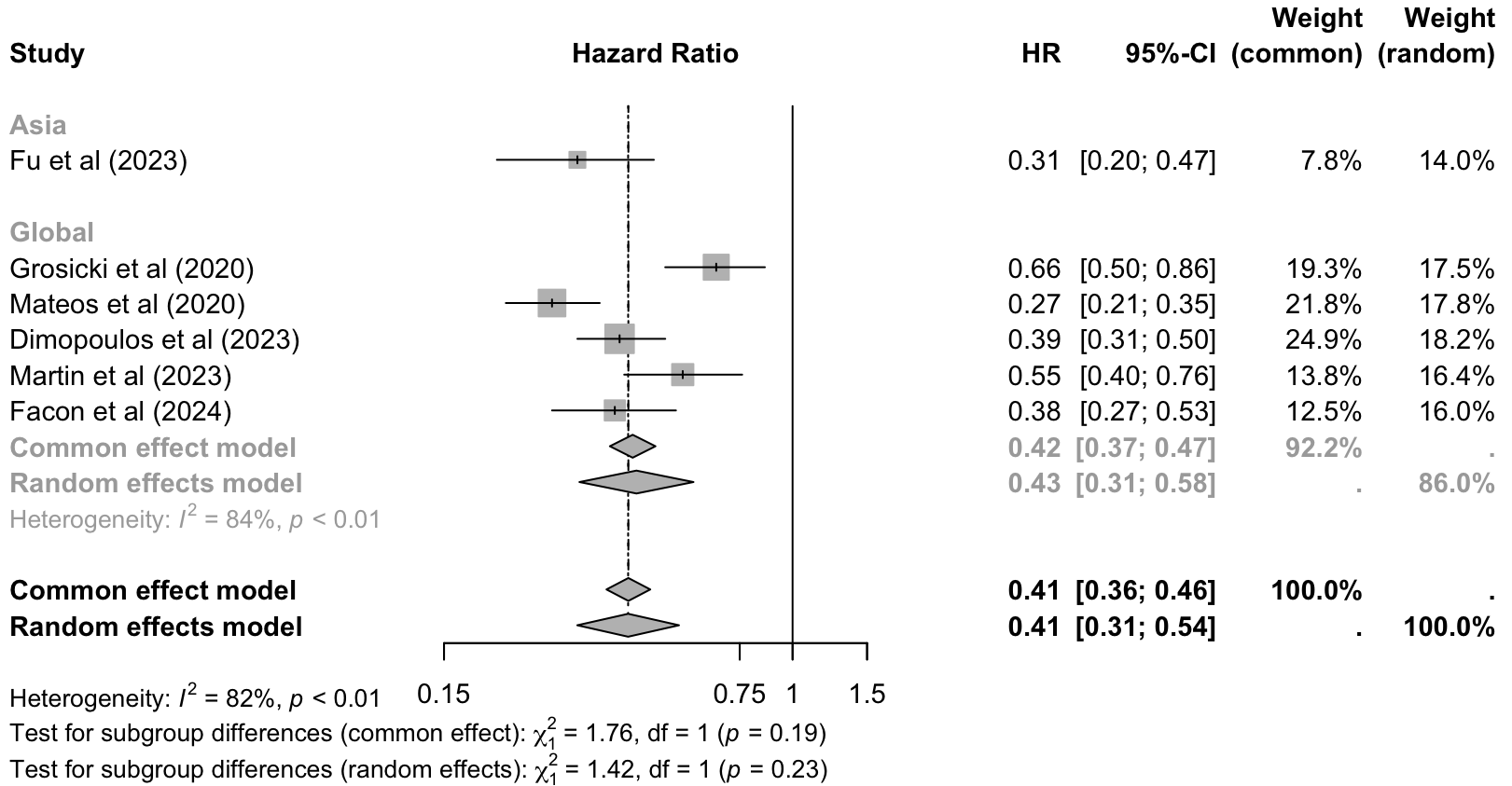


**Figure S85.** TTNT (HR) pooled-estimate for the base-case analysis (by treatment)


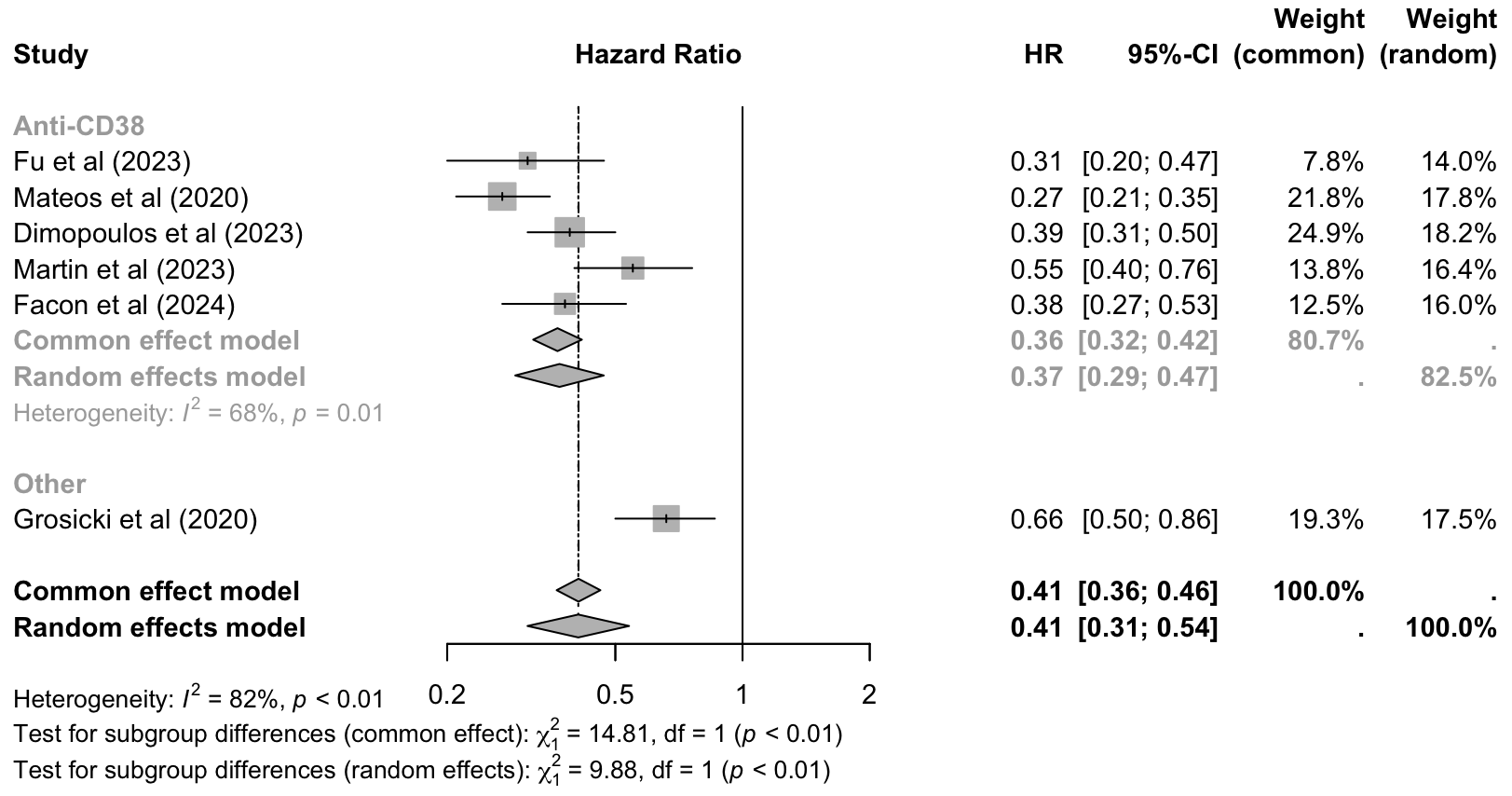


**Figure S86.** TTNT (HR) pooled-estimate for the base-case analysis (by adjustment)


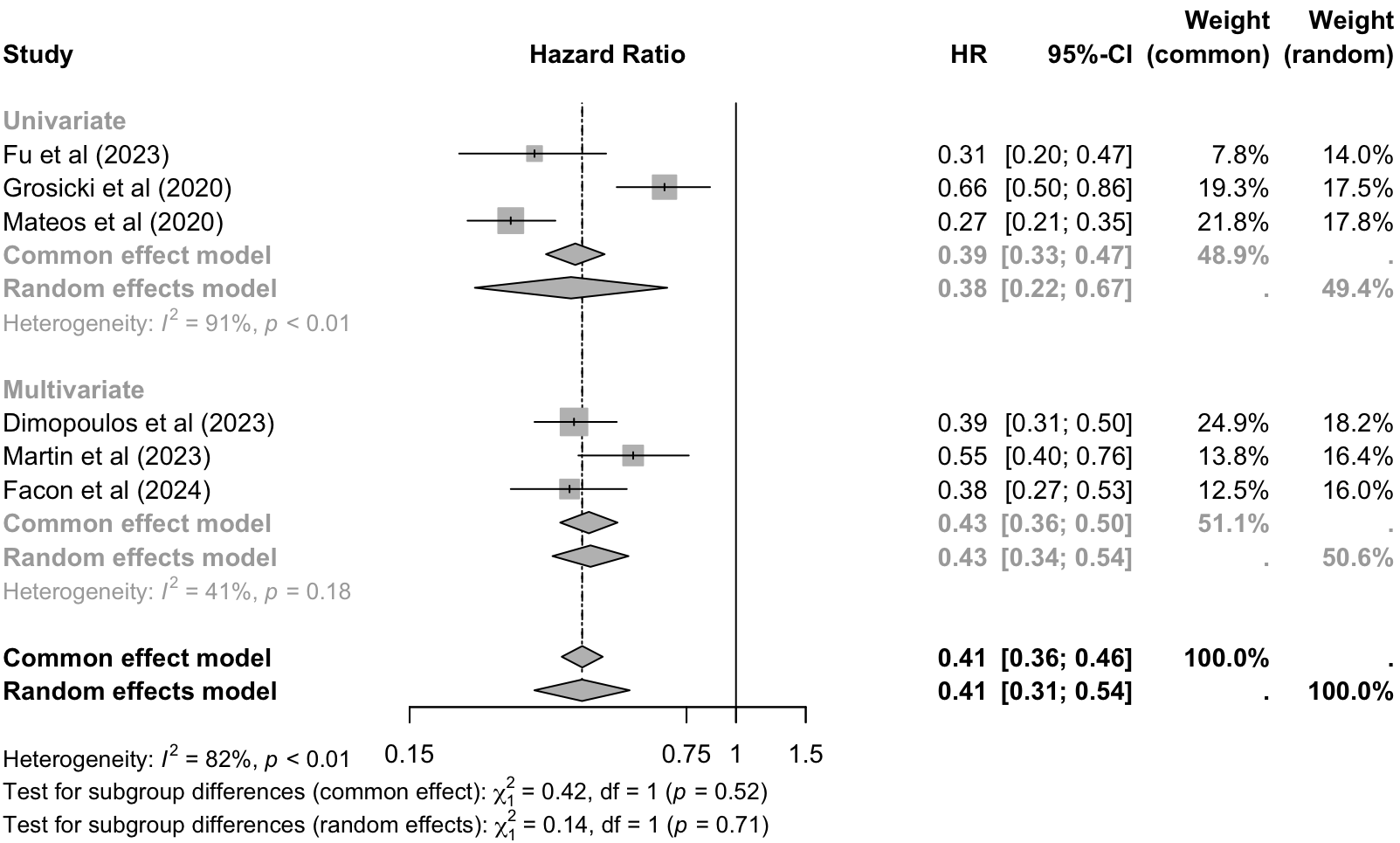


**Figure S87.** Funnel Plot for MRD ORs


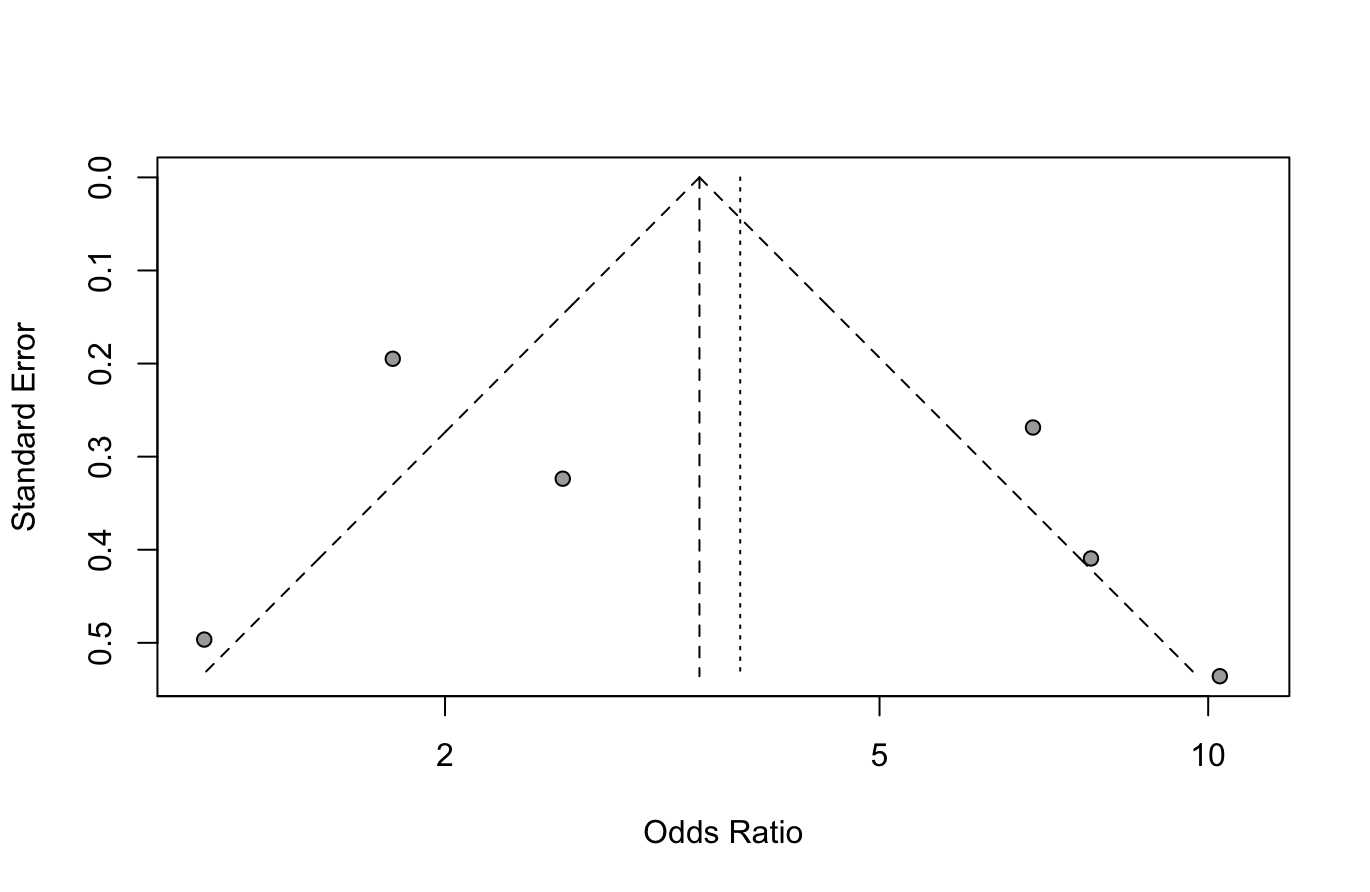


**Figure S88.** Funnel plot for PFS HRs


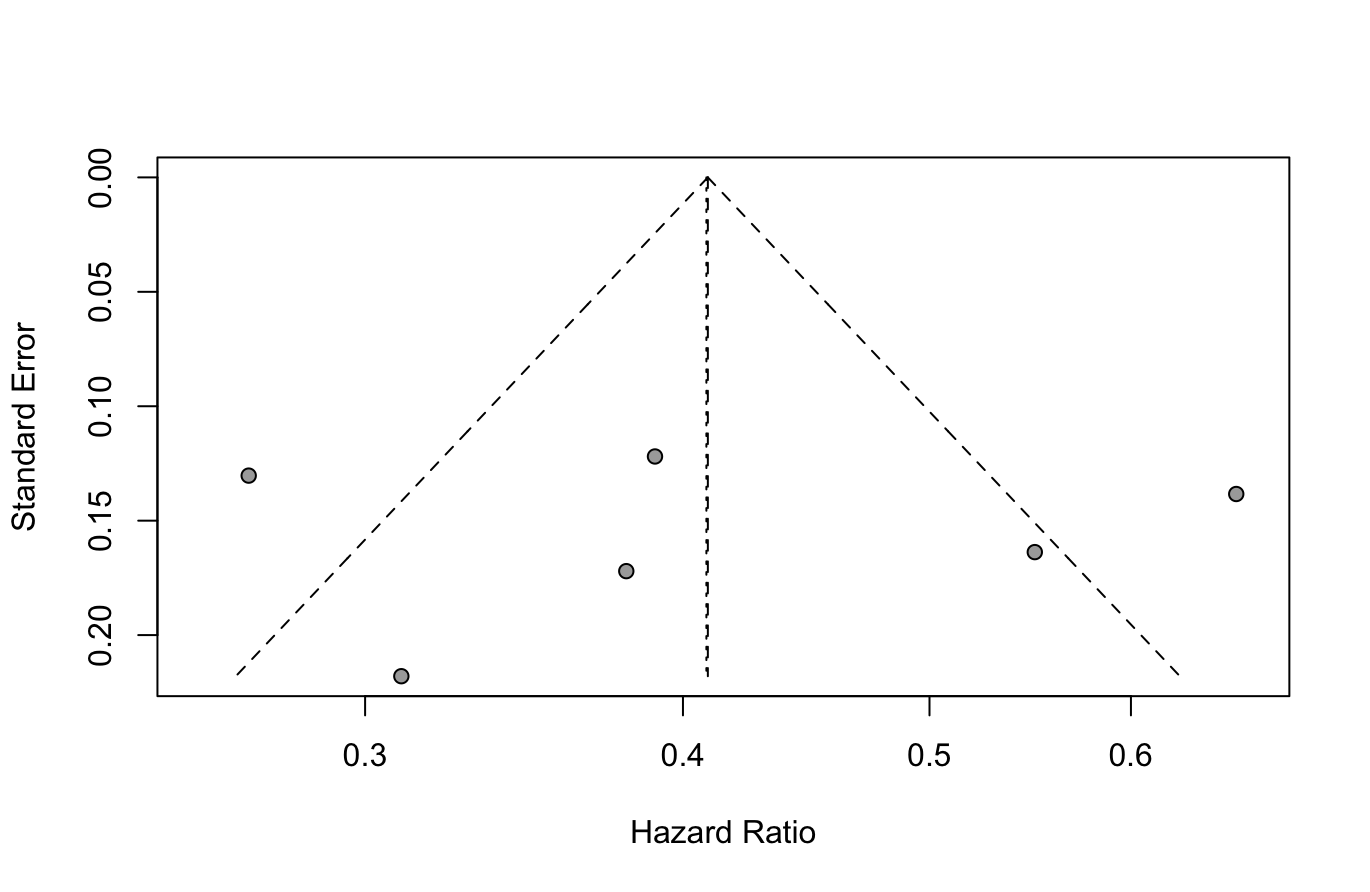


*PFS in MRD negative vs. MRD positive patients*

**Figure S89.** PFS (HR) pooled-estimate (by setting)


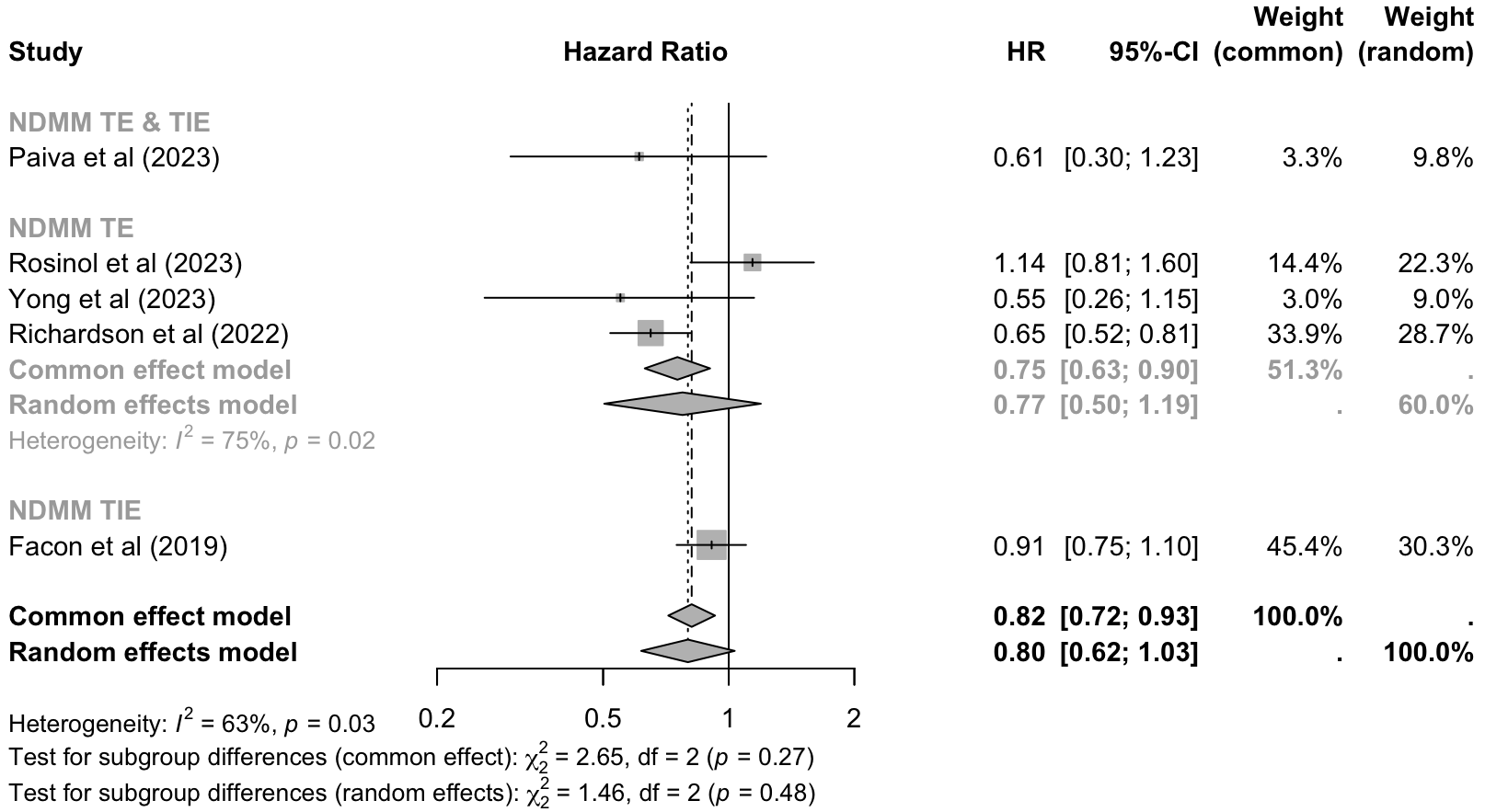


**Figure S90.** PFS (HR) pooled-estimate (by region)


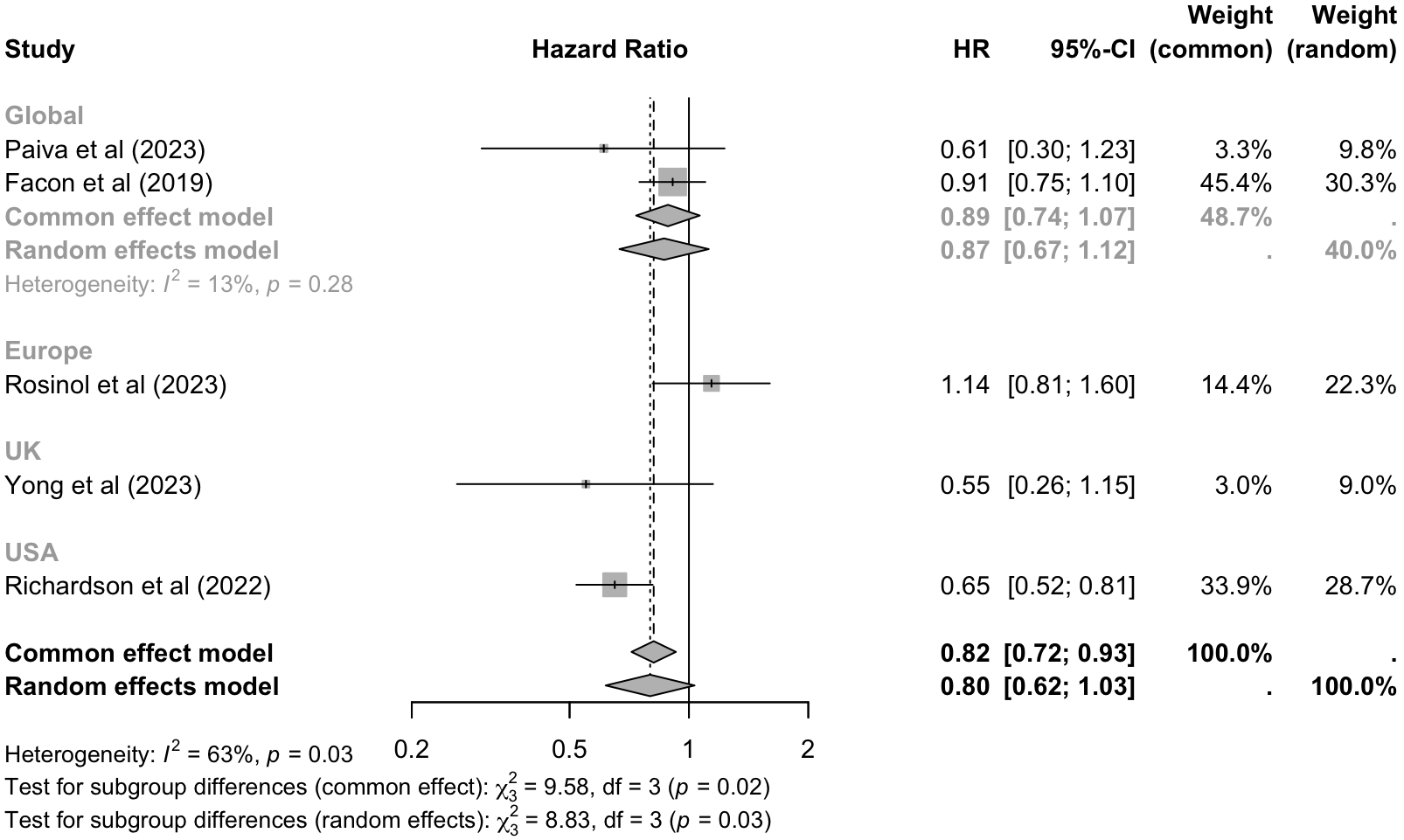


**Figure S91.** PFS (HR) pooled-estimate (by treatment)


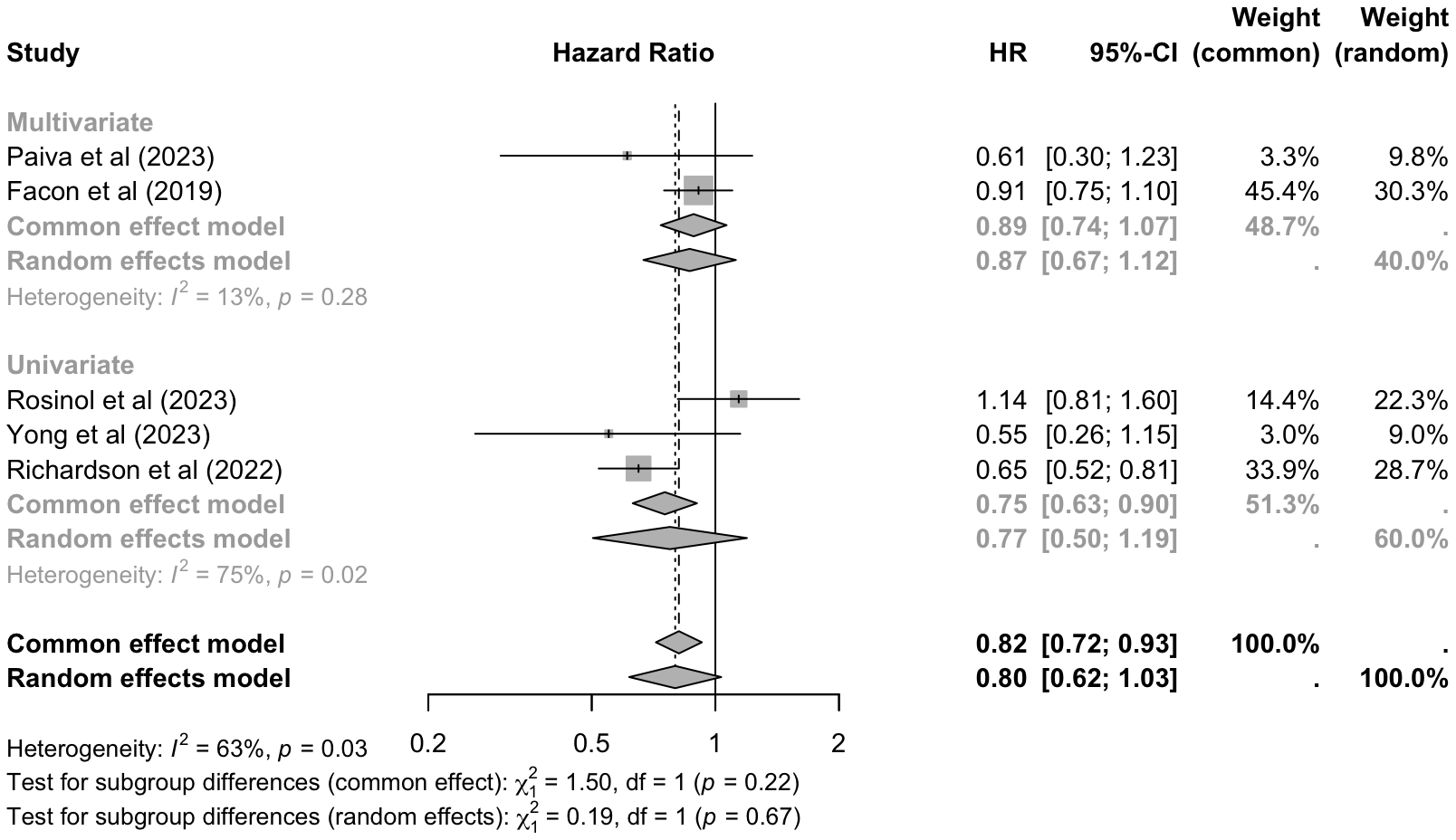


**Figure S92.** Funnel plot for PFS in MRD negative vs. MRD positive patients


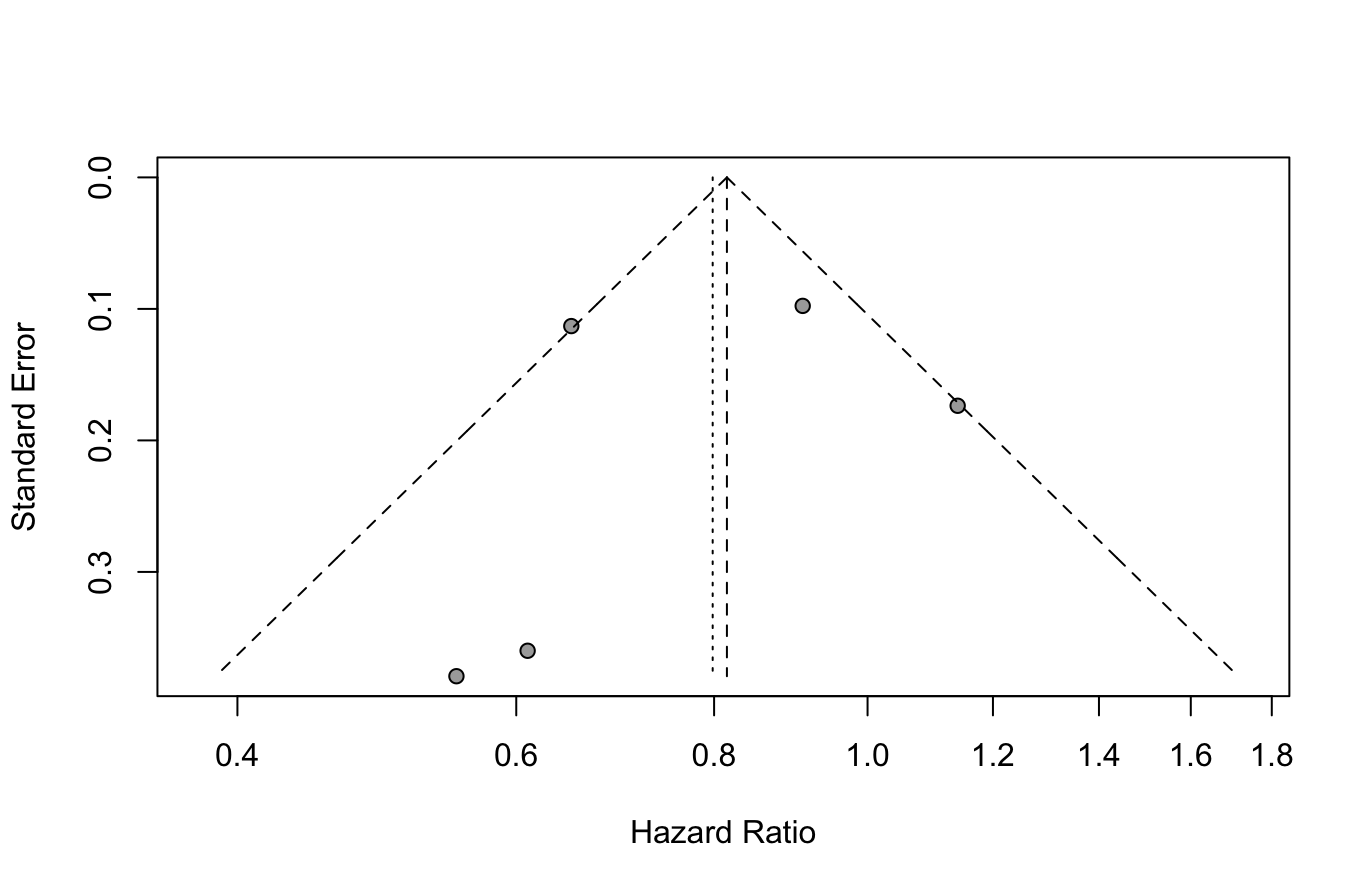


**Figure SR1.** Univariate regression of PFS HR on MRD Negativity OR for NDMM patients (all population)

**
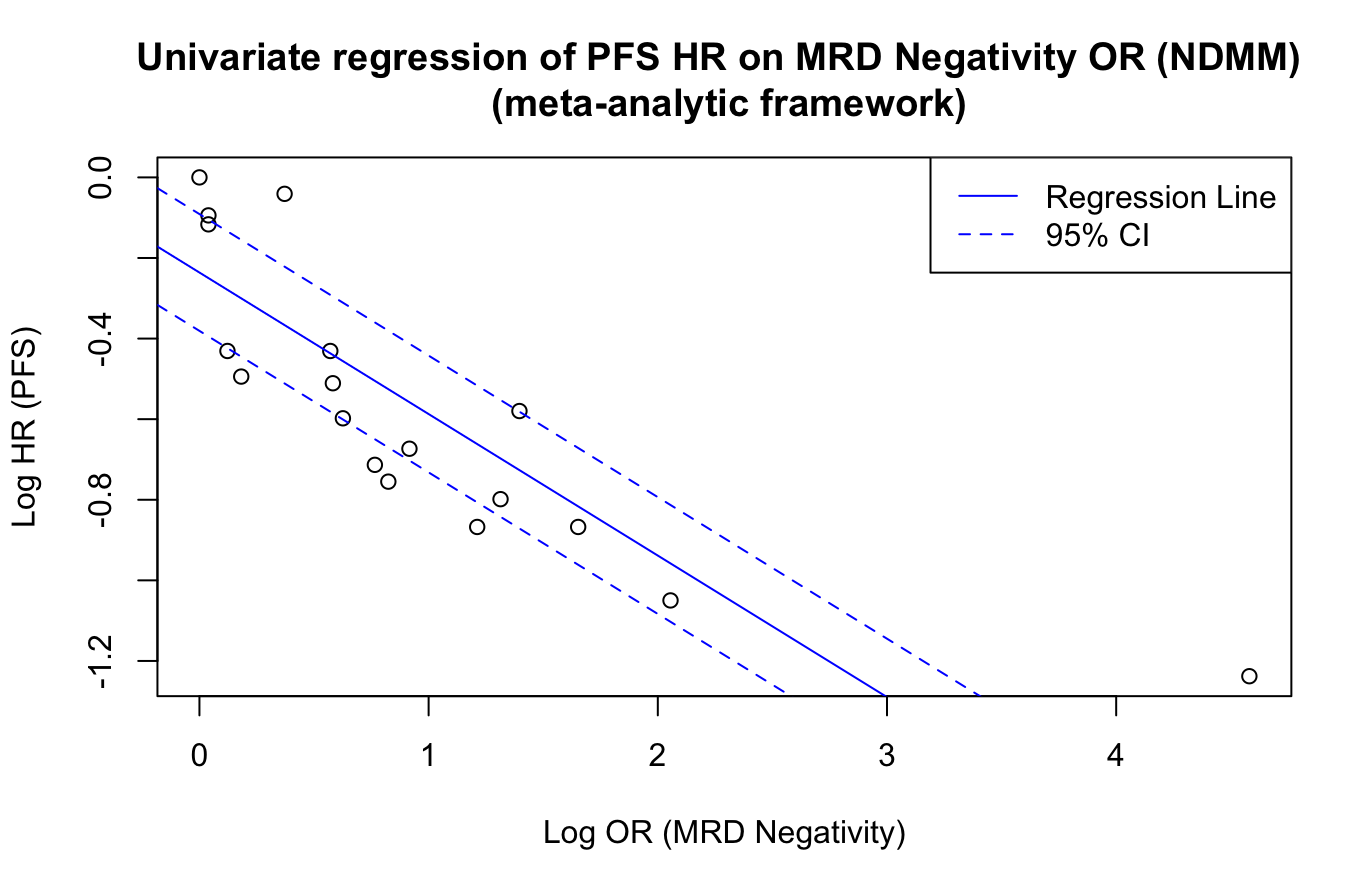
**

**Figure SR2.** Univariate regression of PFS HR on MRD Negativity OR for RRMM patients (all population)


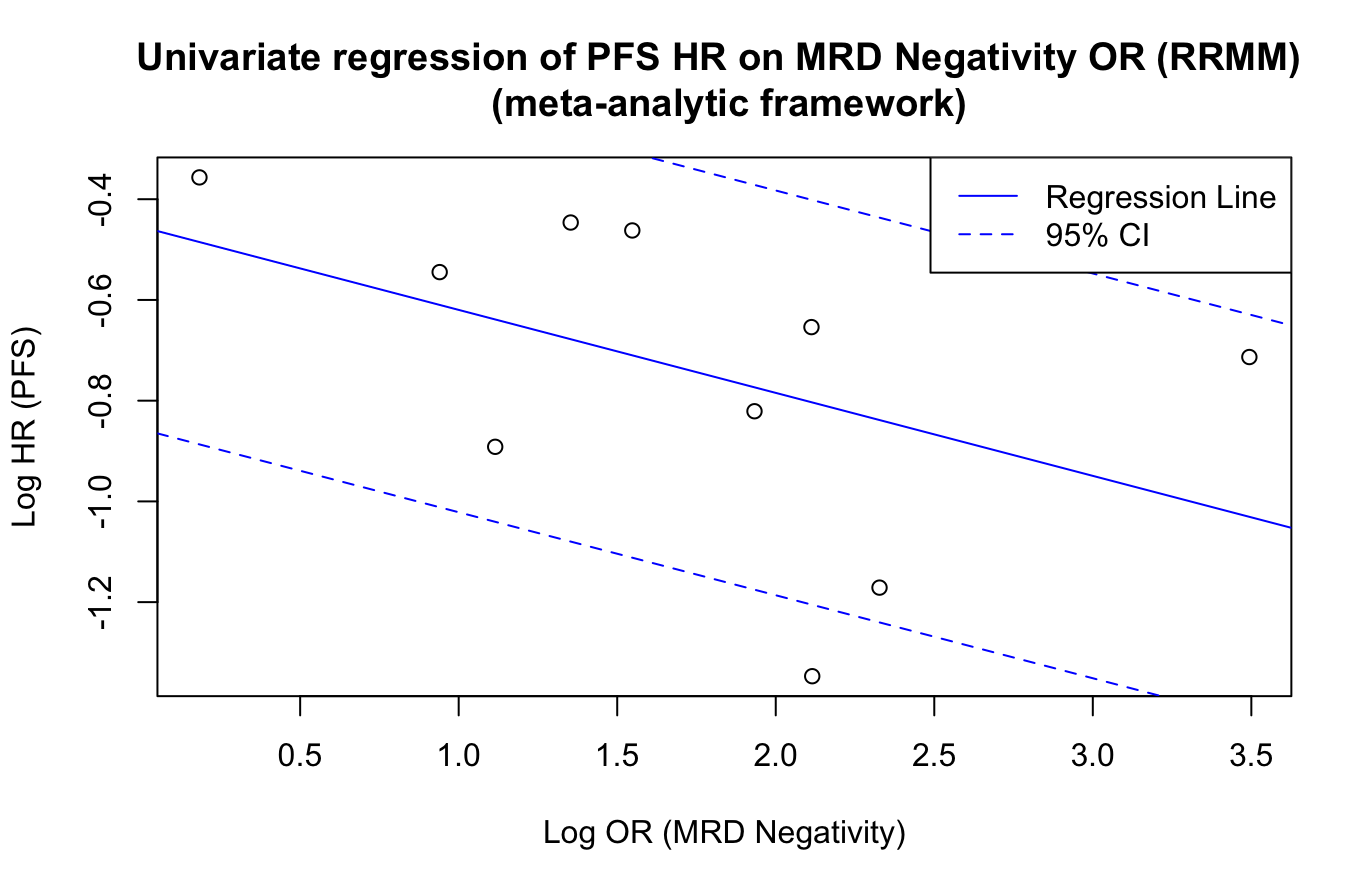


**Table S1.** Meta-regression of PFS HR on MRD Negativity OR with moderators (for base case analysis of longest follow-ups)

| **Variables** | **Coefficient** | **Significance (p-value)** |
| --- | --- | --- |
| Intercept | -0.36 | 0.043* |
| Log OR (MRD Negativity) | -0.17 | 0.017* |
| MM disease setting  NDMM TIE  RRMM | 0.06  0.20 | 0.721  0.234 |
| MRD assessment tech. = NGS | 0.36 | 0.057 |
| MRD sensitivity = 10^-6^ | 0.29 | 0.311 |

**Table S2.** Meta-regression of OS HR on MRD Negativity OR with moderators (for base case analysis of longest follow-up

| **Variables** | **Coefficient** | **Significance (p-value)** |
| --- | --- | --- |
| Intercept | 0.11 | 0.479 |
| Log OR (MRD Negativity) | -0.06 | 0.373 |
| MM disease setting  NDMM TIE  RRMM | -0.21  0.110 | 0.211  0.503 |
| MRD assessment tech. = NGS | -0.47 | 0.003** |
| MRD sensitivity = 10^-6^ | 0.23 | 0.634 |

**Table S3.** Meta-regression of PFS HR on MRD Negativity OR with moderators (for 1-year follow-up analysis)

| **Variables** | **Coefficient** | **Significance (p-value)** |
| --- | --- | --- |
| Intercept | -0.65 | 0.263 |
| Log OR (MRD Negativity) | -0.13 | 0.586 |
| MM disease setting  NDMM TIE  RRMM | 0.17  0.24 | 0.815  0.697 |
| MRD assessment tech. = NGS | -0.361 | 0.363 |
| MRD sensitivity = 10^-6^ | NA | NA |

**Table S4.** Meta-regression of PFS HR on MRD Negativity OR with moderators (for 2-year follow-up analysis)

| **Variables** | **Coefficient** | **Significance (p-value)** |
| --- | --- | --- |
| Intercept | -0.17 | 0.573 |
| Log OR (MRD Negativity) | -0.16 | 0.048* |
| MM disease setting  NDMM TIE  RRMM | 0.11  -0.11 | 0.753  0.776 |
| MRD assessment tech. = NGS | -0.361 | 0.363 |
| MRD sensitivity = 10^-6^ | 0.169 | 0.674 |

**Table S5.** Meta-regression of PFS HR on 12-month sustained MRD Negativity OR with MM disease setting moderator (for the 12-month sustained MRD negativity analysis)

| **Variables** | **Coefficient** | **Significance (p-value)** |
| --- | --- | --- |
| Intercept | -0.38 | 0.001** |
| Log OR (MRD Negativity) | -0.35 | 0.001** |
| MM disease setting  NDMM TE & TIE  NDMM TIE  RRMM | 0.12  0.18  0.18 | 0.545  0.331  0.367 |

**Table S6.** Risk of bias assessment

| **Author (year)** | **Randomization process** | **Deviations from intended interventions** | **Missing outcome data** | **Measurement of the outcome** | **Selection of the reported result** | **Overall bias** |
| --- | --- | --- | --- | --- | --- | --- |
| Horvath et al (2019) | Low | Low | Low | Low | Low | Low |
| Voorhees et al (2020) | Low | Low | Low | Low | Low | Low |
| Voorhees et al (2023) | Low | Low | Low | Low | Low | Low |
| Sonneveld et al (2024) | Low | Low | Low | Low | Low | Low |
| Dytfeld et al (2023) | Low | Low | Low | Low | Low | Low |
| Moreau et al (2019) | Low | Low | Low | Low | Low | Low |
| Moreau et al (2021) | Low | Low | Low | Low | Low | Low |
| Moreau et al (2024) | Low | Low | Low | Low | Low | Low |
| Attal et al (2017) | Low | Low | Low | Low | Low | Low |
| Dimopoulos et al (2019) | Low | Low | Low | Low | Low | Low |
| Rosinol et al (2023) | Low | Low | Low | Low | Low | Low |
| Yong et al (2023) | Low | Low | Low | Low | Low | Low |
| Richardson et al (2022) | Low | Low | Low | Low | Low | Low |
| Paiva et al (2023) | Low | Low | Low | Low | Low | Low |
| Paiva et al (2023) | Low | Low | Low | Low | Low | Low |
| Fu et al (2023) | Low | Low | Low | Low | Low | Low |
| Fu et al (2023) | Low | Low | Low | Low | Low | Low |
| Kumar et al (2020) | Low | Low | Low | Low | Low | Low |
| Facon et al (2021) | Low | Low | Low | Low | Low | Low |
| Mateos et al (2018) | Low | Low | Low | Low | Low | Low |
| Mateos et al (2020) | Low | Low | Low | Low | Low | Low |
| Facon et al (2019) | Low | Low | Low | Low | Low | Low |
| Bringhen et al (2023) | Low | Some concerns | Some concerns | Some concerns | Low | Some concerns |
| Facon et al (2024) | Low | Low | Low | Low | Low | Low |
| Dimopoulos et al (2021) | Low | Low | Low | Low | Low | Low |
| Grosicki et al (2020) | Low | Low | Low | Low | Low | Low |
| Usmani et al (2022) | Low | Low | Low | Low | Low | Low |
| Spencer et al (2018) | Low | Low | Low | Low | Low | Low |
| Mateos et al (2020) | Low | Low | Low | Low | Low | Low |
| Dimopoulos et al (2016) | Low | Low | Low | Low | Low | Low |
| Dimopoulos et al (2023)a | Low | Low | Low | Low | Low | Low |
| Moreau et al (2021) | Low | Low | Low | Low | Low | Low |
| Martin et al (2023) | Low | Low | Low | Low | Low | Low |
| Hungria et al (2024) | Low | Low | Low | Low | Low | Low |
| Dimopoulos et al (2024) | Low | Low | Low | Low | Low | Low |
| Rodriguez-Otero et al (2023) | Low | Low | Low | Low | Low | Low |
| San-Miguel et al (2023) | Low | Low | Low | Low | Low | Low |
| Dimopoulos et al (2023)b | Low | Low | Low | Low | Low | Low |
